# De novo mutation rates at the single-mutation resolution in a human *HBB* gene region associated with adaptation and genetic disease

**DOI:** 10.1101/gr.276103.121

**Published:** 2022-03

**Authors:** Daniel Melamed, Yuval Nov, Assaf Malik, Michael B. Yakass, Evgeni Bolotin, Revital Shemer, Edem K. Hiadzi, Karl L. Skorecki, Adi Livnat

**Affiliations:** 1 Department of Evolutionary and Environmental Biology, University of Haifa, Haifa 3498838, Israel;; 2 Institute of Evolution, University of Haifa, Haifa 3498838, Israel;; 3 Department of Statistics, University of Haifa, Haifa 3498838, Israel;; 4 Bioinformatics Unit, Faculty of Natural Sciences, University of Haifa, Haifa 3498838, Israel;; 5 West African Centre for Cell Biology of Infectious Pathogens (WACCBIP), Department of Biochemistry, Cell and Molecular Biology, University of Ghana, Legon-Accra 00233, Ghana;; 6 Assisted Conception Unit, Lister Hospital and Fertility Centre, Accra CT966, Ghana;; 7 The Ruth and Bruce Rappaport Faculty of Medicine and Research Institute, Technion—Israel Institute of Technology, Haifa 3525433, Israel;; 8 The Azrieli Faculty of Medicine, Bar-Ilan University, Safed 1311502, Israel

## Abstract

Although it is known that the mutation rate varies across the genome, previous estimates were based on averaging across various numbers of positions. Here, we describe a method to measure the origination rates of target mutations at target base positions and apply it to a 6-bp region in the human hemoglobin subunit beta (*HBB*) gene and to the identical, paralogous hemoglobin subunit delta (*HBD*) region in sperm cells from both African and European donors. The *HBB* region of interest (ROI) includes the site of the hemoglobin S (HbS) mutation, which protects against malaria, is common in Africa, and has served as a classic example of adaptation by random mutation and natural selection. We found a significant correspondence between de novo mutation rates and past observations of alleles in carriers, showing that mutation rates vary substantially in a mutation-specific manner that contributes to the site frequency spectrum. We also found that the overall point mutation rate is significantly higher in Africans than in Europeans in the *HBB* region studied. Finally, the rate of the 20A→T mutation, called the “HbS mutation” when it appears in *HBB*, is significantly higher than expected from the genome-wide average for this mutation type. Nine instances were observed in the African *HBB* ROI, where it is of adaptive significance, representing at least three independent originations; no instances were observed elsewhere. Further studies will be needed to examine mutation rates at the single-mutation resolution across these and other loci and organisms and to uncover the molecular mechanisms responsible.

It is widely known that mutation rates vary across the genome at multiple scales (Hodgkinson and Eyre-Walker 2011; Rahbari et al. 2016; Carlson et al. 2018) and are affected by multiple factors, from the mutation type (Gojobori et al. 1982; Bulmer 1986), to the local genetic context (Gojobori et al. 1982; Bulmer 1986; Blake et al. 1992; Hwang and Green 2004; Rahbari et al. 2016; Carlson et al. 2018), to the general location in the genome (Wolfe et al. 1989; Matassi et al. 1999; Lercher et al. 2001; Ellegren et al. 2003). Although this knowledge is highly advanced now compared with what was known a mere decade ago (Campbell et al. 2012; Michaelson et al. 2012; Francioli et al. 2015; Rahbari et al. 2016; Carlson et al. 2018), it could be enhanced further. In particular, rate measurements to date all have been based on averages of various kinds, such as an average across the genome (Nachman and Crowell 2000; Rahbari et al. 2016), or across the instances of any particular motif (Hwang and Green 2004; Carlson et al. 2018), or in certain cases, across the entire stretch of a gene (Haldane 1949; Vogel and Motulsky 1997; Kondrashov 2003). In contrast, technological limitations have precluded measuring mutation rates at particular base positions and of particular mutations at such positions. However, such high-resolution knowledge of the mutation rate variation would bear on multiple open questions in genetics and evolution—from the relative importance of mutation rate variation to the site frequency spectrum (SFS) (Harpak et al. 2016; Lek et al. 2016; Mathieson and Reich 2017), to its importance for adaptive evolution and parallelism (Inoue et al. 2001; Crow et al. 2009; Dumas et al. 2012; Losos 2017; Kratochwil et al. 2019; Kratochwil and Meyer 2019; Lind 2019; Xie et al. 2019), to its contribution to recurrent genetic disease and cancer (Lupski 1998; McClellan and King 2010; Veltman and Brunner 2012; Shendure and Akey 2015).

The most precise way of measuring mutation rates, free of biases attributable to past natural selection or random genetic drift events, is offered by de novo mutations—mutations that appeared for the first time in their carrier (Goldmann et al. 2016; Rahbari et al. 2016). These mutations are usually detected by studies comparing the genomes of children to those of their parents, also known as “trio studies” (Roach et al. 2010; Conrad et al. 2011). However, because each individual carries only a small number (e.g., several dozen in humans) of de novo mutations scattered across the genome, the chance of encountering any particular target mutation of interest is miniscule, rendering it impractical to measure rates of target mutations using such studies.

To overcome this barrier, we have developed a method that enables identifying and counting, with high accuracy, ultrarare genetic variants of choice in extremely narrow regions of interest (ROIs) within large populations of cells, such as a single target mutant in 100 million genomes. Because this method has both an error rate lower than the human mutation rate and sufficient yield for the purpose, it enables measuring the frequencies of target mutations of choice in human sperm samples by counting their de novo instances at a single-digit resolution. For variants that are not expected to affect sperm fertility and viability (as in the case below), this frequency is the evolutionarily relevant mutation rate in males. Note that aside from this evolutionary application, ultra-accurate methods of mutation-detection are sought after for early detection of cancer, noninvasive prenatal testing, early identification of virus within host, and more (Salk et al. 2018).

As a first target for this method, we chose two sites: a 6-bp region spanning three codons within the human hemoglobin subunit beta (*HBB*) gene that is of great importance for adaptation and hematologic disease, and the identical, paralogous region within the hemoglobin subunit delta (*HBD*) gene. The former region includes, among others, the site of the hemoglobin S (HbS) mutation. The most iconic balanced polymorphism mutation (Pauling et al. 1949; Allison 1954; Ingram 1957; Cavalli-Sforza and Feldman 2003; Feng et al. 2004; Hartl and Clark 2007), the HbS mutation is an A to T transversion (GAG→GTG, Glu→Val) in codon 6 of *HBB* causing sickle-cell anemia in homozygotes (Pauling et al. 1949) and providing substantial protection against severe malaria in heterozygotes (Allison 1954; Flint et al. 1998; Kwiatkowski 2005; Piel et al. 2010). Malaria, in turn, has been a leading cause of human morbidity and mortality, often causing more than a million deaths per year in the recent past, with Africa bearing the brunt of the disease burden (Carter and Mendis 2002), and thus has been possibly the strongest known agent of selection in humans in recent history (Kwiatkowski 2005). Besides the HbS mutation, many other mutations, both point mutations and indels, are also known at this site, many of which are involved in hematologic illness (Hardison et al. 2002; Hardison and Miller 2002). In contrast to *HBB*, mutations in *HBD* have a more limited effect and are not thought to confer resistance to malaria, because the *HBD*’s lower expression levels make it account for <3% of the circulating red blood cell hemoglobin in adults (Steinberg and Adams 1991). Although the population prevalence of the *HBB* mutations, whether beneficial or detrimental, is normally attributed to natural selection, so far it has not been possible to examine to what degree, if at all, mutational phenomena may also be relevant to their prevalence. To address this gap, we sought to characterize the rates of mutations, including the HbS mutation, in the *HBB* and *HBD* ROIs in sperm samples of both African and European donors.

## Results

**Figure 1.**
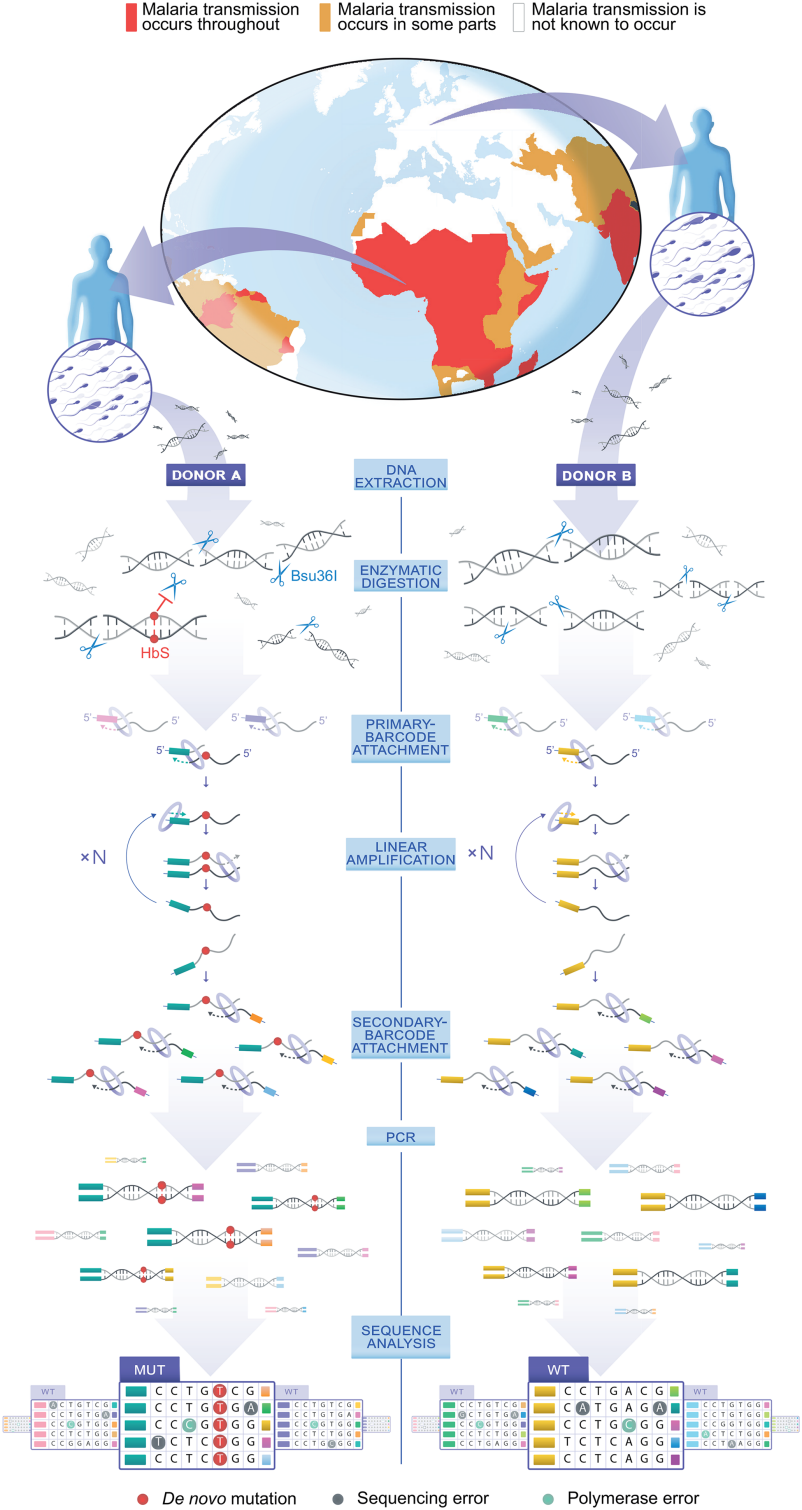
Experiment overview. Sperm samples are obtained from world regions with high or low malaria infection burden (malaria impact map adjusted from the CDC map) (CDC Division of Parasitic Diseases and Malaria 2019). Whole-genome DNA is extracted and an amount equivalent to 60–80 million sperm cells per donor is subjected to Bsu36I digestion. Bsu36I cleaves the DNA at multiple sites, including the *HBB* and *HBD* ROIs, which carry a specific recognition sequence. The HbS mutation blocks Bsu36I digestion and is thus enriched over the wild-type (WT). A primary barcode is added directly to each antisense DNA strand that carries the *HBB* or *HBD* ROI via a DNA polymerase–assisted fill-in reaction. Because each barcode consists of a random sequence of nucleotides, each of the numerous target fragments has its own unique barcode, illustrated by a unique color on the *left* end of the representation of each barcoded fragment. Multiple single-strand copies are each generated directly from each uniquely barcoded target fragment by linear amplification. A secondary barcode composed of a random sequence of nucleotides is added to the other end of each of these copies by a single primer extension reaction, illustrated by a unique color on the *right* end of each barcoded fragment. Thus, only full-length fragments (i.e., mutant or WT ROI sequences that evaded Bsu36I digestion) carry both the primary and the secondary barcodes and can be amplified by PCR for high-throughput sequencing. At the sequence analysis step, sequencing reads representing the PCR products of the linearly amplified copies are grouped together into families (see boxes), where in each family, reads share the same primary barcode sequence. Sporadic sequencing errors or DNA-polymerase errors generated during linear or subsequent amplification steps are unlikely to be repeated in multiple copies and are removed. De novo mutations, such as the HbS mutation, are easily identified by their appearance in multiple reads from distinct linear-amplification events. For a complete description of the library preparation protocol, which includes additional steps, see Supplemental Figures S1–S3.

To substantially reduce the false positive rate resulting from PCR amplification or high-throughput sequencing errors, following extraction of the DNA from the sperm of the donors, we first remove the majority of wild-type (WT) ROI molecules from each sample. Specifically for the target sites, we use the restriction enzyme (RE) Bsu36I, which cleaves the WT sequence CCTGAGG at positions 16–22 of *HBB* and the paralogous positions of *HBD* while leaving the HbS mutant and other mutants in these positions intact. Besides substantially reducing the false positive rate, this WT depletion has the additional benefit of reducing the sequencing costs by the same factor, because it removes the majority of fragments whose sequences are known to be WT (Fig. 1; Supplemental Text; Supplemental Figs. S1–S4).

Importantly for the mutation rate calculation, we keep track of the number of WT molecules removed by accurately calculating the protected mutants’ enrichment factor on a per sample basis. For this purpose, we generate two mixtures, each of which includes, in addition to the DNA studied, known amounts of mock DNA that is resistant to the RE digestion (Supplemental Text S2; Supplemental Fig. S2). Next, we apply the same protocol to the two mixtures, with the exception that the RE digestion step is applied to only one of them (Supplemental Text S2; Supplemental Fig. S2). The ratio of the ratios of sensitive to resistant molecules identified for the two mixtures after treatment at the sequence analysis step provides the enrichment factor of the protected mutants (Supplemental Text S2; Supplemental Fig. S2). This enrichment factor, multiplied by the number of WT molecules called, with the addition of the small number of mutants called, provides the number of cells analyzed (Supplemental Text S2; Supplemental Fig. S2). We set up the system in such manner that the calculation of the enrichment factor depends only on quantities that are precisely known, including volume measurements (Supplemental Text S2; Supplemental Fig. S2) and numbers of WT and mutant molecules called during the barcode-based sequence analysis stage as described below.

Following this mutation enrichment step, we attach unique barcodes to the DNA fragments to reduce error by consensus sequencing of copies originating from the same original fragment. For this purpose, we build on and improve the maximum depth sequencing method (MDS) (Jee et al. 2016), which allows one to focus on a narrow region of interest (ROI) and whose key idea is to attach the barcodes directly to a cleaved end of one of the two strands of each original target DNA fragment via a DNA polymerase–assisted extension reaction, as opposed to including the barcode only in the first copy of the DNA by extending the target-specific primer that carries it. In this manner, errors that occur during the first critical copying step are also detected via consensus sequencing of reads sharing the same barcode (Fig. 1; Supplemental Text; Supplemental Fig. S1; Jee et al. 2016). To all of the above, we add multiple innovations that increase sequencing accuracy, handle the large amounts of genomic DNA required, and enable accurate measurement of the Bsu36I enrichment factor per sample as needed for the mutation rate calculation (Supplemental Figs. S1–S5). We refer to this whole method as mutation enrichment followed by upscaled maximum depth sequencing (MEMDS) (for a complete protocol, see Supplemental Text S1–S9 and Methods).

**Figure 2.**
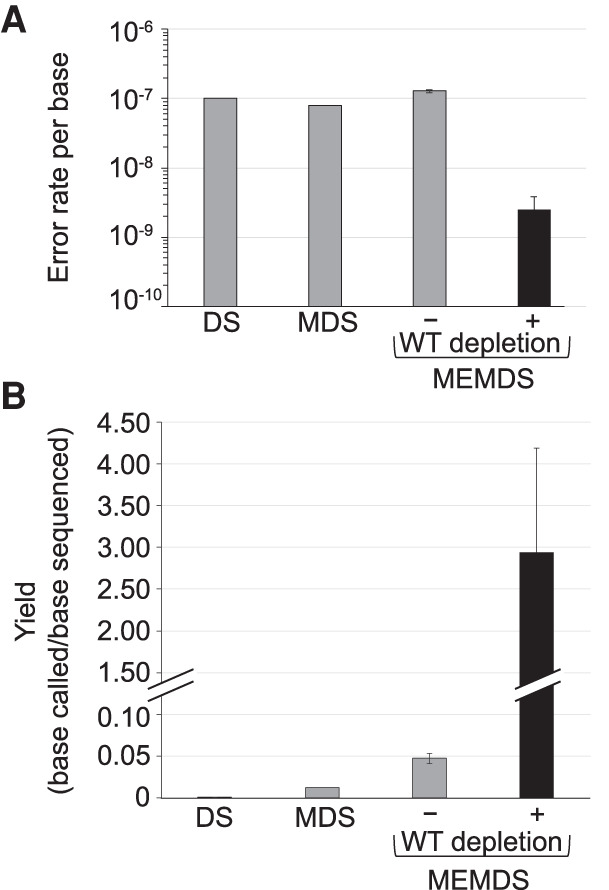
Accuracy and yield of MEMDS compared with current cutting-edge methods for studying target regions. (*A*) Under a highly conservative estimate, MEMDS increases accuracy by at least 40-fold compared to duplex sequencing (DS) (Kennedy et al. 2014) and maximum depth sequencing (MDS) (Jee et al. 2016). (*B*) MEMDS also increases yield per sequenced base (i.e., the number of MEMDS confirmed bases divided by the number of paired-end sequenced bases) by orders of magnitude over both DS and MDS (Kennedy et al. 2014; Jee et al. 2016). Notice that in MEMDS, the yield can be higher than 1 because the mutation enrichment factor is accurately calculated (Supplemental Text S2) and the base identity is known for the ROI sequences that were digested and removed from the final sequencing libraries (they have the restriction enzyme recognition sequence). Although the accuracy of DS has been improved in the context of sequencing large parts of the genome (Abascal et al. 2021), yield considerations and other limitations preclude applying current DS-based methods to narrow ROIs and target mutations (Kennedy et al. 2014; Supplemental Text S1) with the same efficiency as that of MEMDS.

Finally, following sequence analysis (Supplemental Figs. S6–S10; Supplemental Table S2) the number of appearances of any mutation that confers resistance to the restriction enzyme is counted and divided by the calculated number of cells analyzed, providing the evolutionarily relevant de novo origination rate for each specific mutation in males per donor and per group of donors (Supplemental Figs. S11–S13; Supplemental Table S3). Following previous literature, we ignore G→T and C→T mutations in the barcoded strand (C→A and G→A in the sequenced strand) because they are thought to reflect not lasting mutations but the experimental disruption of an ongoing in vivo process of base damage and repair as well as in vitro mutations attributed to guanine oxidation and cytosine deamination (Supplemental Text S8; Supplemental Figs. S12–S14; Arbeithuber et al. 2016; Jee et al. 2016). In addition, we exclude C→A, the complement of G→T, owing to its association with the latter and its frequent appearance in the data (Supplemental Text S8; Supplemental Fig. S12). Following normal loss of material of ∼65%, true positives of non G→T, C→T, and C→A mutations are identified with a false positive rate (error rate) <2.5 × 10^−9^ per base (Fig. 2). Overall, MEMDS surpasses recent cutting-edge methods in both accuracy (Fig. 2A) and yield (Fig. 2B; see also Supplemental Fig. S11).

With the help of this method, we examined a total of more than half a billion gene fragments individually taken from sperm of 12 donors. Because one of the samples was a mixture from two African donors with a total number of cells similar to the other African samples, we consider it here as a single sample of mixed African origins, bringing the total to 11 samples, seven from African and four from European donors (Supplemental Table S1). The numbers of cells scanned and de novo mutations observed per person are shown in Table 1.

**Table 1.**
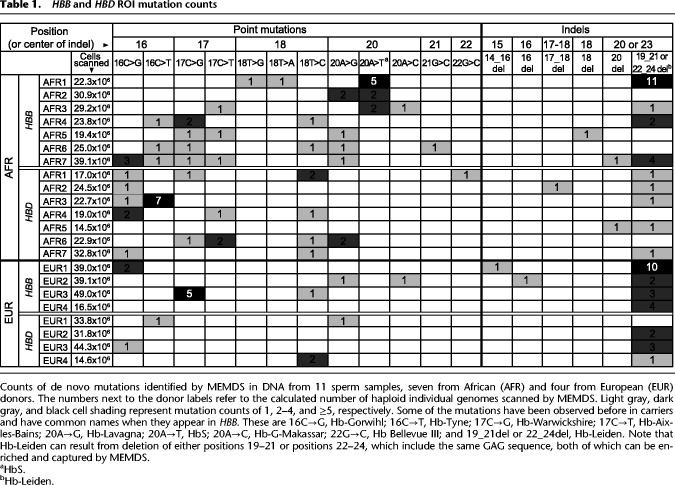
*HBB* and *HBD* ROI mutation counts

### Average per ROI mutation rates

The average per base point mutation rates in the *HBB* and *HBD* ROIs are 3.3 × 10^−8^ and 2.79 × 10^−8^, respectively, significantly higher by ∼2.6-fold (*P* < 2 × 10^−8^, 95% CI 2.4 × 10^−8^–4.4 × 10^−8^) and ∼2.2-fold (*P* < 6.7 × 10^−5^, 95% CI 1.9 × 10^−8^–4 × 10^−8^, two-sided binomial exact test) than 1.25 × 10^−8^, which we use as an estimate of the genome-wide per base per generation point mutation rate (Supplemental Text S10). The average indel rates in these ROIs were 1.1 × 10^−8^ and 4.3 × 10^−9^, respectively, significantly higher by approximately ninefold (*P* < 4.3 × 10^−25^, 95% CI 8 × 10^−9^–1.5 × 10^−8^) and ∼3.4-fold (*P* < 1.8 × 10^−4^, 95% CI 2.3 × 10^−9^–7.3 × 10^−9^; two-sided binomial exact test) than the expected 1/10 of the point mutation rate (Supplemental Text S10). The average point mutation rate of the *HBB* ROI is not significantly higher than that of the *HBD* ROI (*P* = 0.49, two-sided Fisher's exact test), and the average indel rate of the former is significantly higher by ∼2.6-fold than that of the latter (*P* = 0.0015, OR 95% CI 1.42–5.01; two-sided Fisher's exact test).

### Basic characteristics of mutation rate variation

The variance in the rates of de novo point mutations is higher than expected from the genome-wide average (GWA) rates of these mutations (e.g., Harris 2015; Harris and Pritchard 2017), and their relative rates are different than expected from the GWA rates (*P* < 10^−6^ in an omnibus multinomial test, adjusted for the excluded mutations, compared to the rates of Rahbari et al. 2016), even when adjusting the latter for the 3-mer, 5-mer, and 7-mer nucleotide contexts (*P* < 10^−5^ in all cases, compared to the rates of Carlson et al. 2018). The overall de novo rates of the six observed deletion types are highly nonuniform (*P* < 10^−6^, multisample proportion test).

### Correspondence between de novo rates and observations of alleles in carriers

The HbS and Hb-Leiden mutations both have been notably observed on multiple different genetic backgrounds in human populations, the former particularly in Africans (Flint et al. 1998; Hardison et al. 2002; Hardison and Miller 2002). Here, they are the point mutation of highest de novo rate in the African *HBB* ROI and the deletion mutation of highest de novo rate in any gene and ethnicity. Furthermore, of the 23 potential deletions of up to size 3 that are observable by our method per ROI, only five deletions (16delC, 17­_18delCT, 18_19delTG, 19_21delGAG or the equivalent 22_24delGAG—the Hb-Leiden mutation—and 20delA) have been reported to date on the HbVar database—a large collection of hemoglobin variants (Hardison et al. 2002; Hardison and Miller 2002)—all in *HBB*; and of these deletion types, a significantly higher fraction is observed here de novo compared to deletion types not reported on HbVar (Supplemental Text S11). Pooling together both the *HBB* and *HBD* ROIs given the similarity of de novo indel types observed between them, this effect is significant both with (*P* = 0.0078, OR 95% CI 2.17–818.08, two-sided Fisher's exact test) and without (*P* = 0.024, OR 95% CI 1.44–653.93, two-sided Fisher's exact test) the Hb-Leiden mutation, showing that the correspondence between de novo rates and alleles in populations extends beyond the HbS and Hb-Leiden mutations. Although the same analysis cannot be repeated for the point mutations because of the smaller number of observable mutation types and the synonymous versus nonsynonymous mutation confound, further observations are in the expected direction (Supplemental Text S11). The correspondence observed could not have been predicted from the mutations’ GWA rates, even when adjusting for the genetic context (Supplemental Text S10, S11).

### Between-population comparisons

To provide a conservative statistical test of a population-level difference that excludes individual- or sample-level variation alone as accounting for the result, we compared the per person overall point mutation rates in the *HBB* ROI between the African and European groups. Results showed that these rates were significantly higher in the African than in the European group both with (*P* = 0.0061) and without (*P* = 0.043, two-sided Wilcoxon rank-sum test) counting the HbS mutation. Next, pooling together cells from all donors within each population to estimate the overall point mutation rate in the *HBB* ROI shows it to be significantly higher by 2.57-fold in the African than in the European donors (*P* < 0.006, OR 95% CI 1.27–5.49, two-sided Fisher's exact test). Thus, there is a significant population-level difference between the continental groups in the overall point mutation rate in this narrow ROI that is not attributable to individual- or sample-level variation. In contrast, in the *HBD* ROI, the number of mutations was not high enough to establish such a difference above and beyond individual- or sample-level variation (*P* = 0.18, two-sided Wilcoxon rank-sum test). In contrast to the *HBB* overall point mutation rate, the overall indel rate did not vary significantly between these groups in either ROI (*P* = 0.35 and *P* = 1, respectively, two-sided Fisher's exact test).

### Position 20 mutation rates

Two particularly notable mutations are the HbS and Hb-Leiden mutations (details below). Considering codons 6 and 7 equivalent with respect to the latter mutation, both mutations can be said to affect position 20. Using the aforementioned conservative test to exclude sample-level variation alone as accounting for the result, the overall per person point mutation rates at position 20 specifically are significantly higher in the *HBB* than in the *HBD* ROI in Africans (*P* = 0.017, two-sided Wilcoxon rank-sum test) but not in Europeans (*P* = 1). In the former, the overall point mutation rate at position 20 pooled across individuals is ∼6.1× higher in *HBB* than in *HBD* (*P* = 0.0061, OR 95% CI 1.50–37.14, two-sided Fisher's exact test). In the case of the overall indel rates at position 20, although the pooled rates are significantly higher in *HBB* than in *HBD* for both Africans and Europeans (*P* = 0.044, OR 95% CI 1.03–6.54 and *P* = 0.027, OR 95% CI 1.11–7.02, respectively; two-sided Fisher's exact tests), sample-level variation cannot be excluded as the source of the differences (*P* = 1 and *P* = 0.69 for Africans and Europeans, respectively; two-sided Wilcoxon rank-sum tests).

### Rates of the Hb-Leiden mutation

The 3-bp in-frame deletion variant of either codon 6 or codon 7 that is called “the Hb-Leiden mutation” when it occurs in *HBB* recurs noticeably more often than other mutations (comparing its per person rates to those of all other deletions combined to exclude sample-level variation, *P* < 0.0005, two-sided Wilcoxon rank-sum test). Pooled across individuals, it appears at rates of 1.11 × 10^−7^ and 3.96 × 10^−8^ in the *HBB* and *HBD* ROIs, respectively, ∼88.86× and ∼31.66× higher than the 1.25 × 10^−9^ estimate (*P* = 4.04 × 10^−58^, 95% CI 7.82 × 10^−8^–1.53 × 10^−7^; and *P* = 1.62 × 10^−13^, 95% CI 1.98 × 10^−8^–7.08 × 10^−8^), where the *HBB* rate is significantly (∼2.81×) higher than the *HBD* rate (*P* = 0.002, OR 95% CI 1.40–5.63, two-sided Fisher's exact test).

### Rates of the HbS mutation

The 20A→T mutation called “the HbS mutation” when it appears in the *HBB* ROI appears nine times in the African *HBB* ROI and no times in the other cases combined (the European *HBB* ROI and the European and African *HBD* ROIs) (*P* = 0.023, 95% CI 1.5077–Inf; two-sided Fisher's exact test classifying each individual and gene case as having [>0] or not having [=0] de novo 20A→T in sperm and comparing the fractions of these classes between the groups). The rate of the HbS mutation in the overall group (Africans and Europeans combined)—2.7 × 10^−8^—is 19.6× higher (*P* < 2 × 10^−9^, rate 95% CI 1.24 × 10^−8^–5.13 × 10^−8^) than expected from the GWA for this mutation type (Supplemental Text S10), and its rate in the African group specifically—4.74 × 10^−8^—is ∼35× higher than expected from its GWA (*P* = 1.2 × 10^−11^, rate 95% CI 2.17 × 10^−8^–9.0 × 10^−8^; two-sided binomial exact test). In the African group, it is the mutation that deviates the most (Supplemental Table S4) from its GWA among the 12 observable point mutations, where its de novo rate varies significantly across samples (*P* = 0.0025, multisample proportion test), from 0 to 2.24 × 10^−7^ (the latter rate being ∼163× faster than expected; *P* = 2.23 × 10^−10^, 95% CI 7.27 × 10^−8^–5.23 × 10^−7^, two-sided binomial exact test). Note that the evolutionarily relevant mutation rate depends on the fraction of the mutation in sperm per se, not on whether it repeats because of independent originations or owing to an early appearance followed by duplications during spermatogenesis. The minimal number of independent originations of the HbS mutation is three, given that three individuals produced it de novo, and the corresponding minimal rate of independent occurrence of the HbS mutation in the sperm samples (a rate lower than the actual evolutionarily relevant mutation rate observed) across all individuals is 9.01 × 10^−9^. This rate is still ∼6.5× higher than the genome-wide evolutionarily relevant mutation rate for this mutation type (*P* = 0.011, 95% rate CI 1.86 × 10^−9^–2.63 × 10^−8^, two-sided binomial exact test).

## Discussion

The data expose an ultrahigh resolution correspondence between de novo mutation rates and past observations of alleles in carriers (Flint et al. 1998; Hardison et al. 2002; Hardison and Miller 2002; Supplemental Text S11; Results), suggesting that these rates contribute to the prevalence of these mutations in populations. This correspondence could not have been predicted from the GWA rates of these mutation types even when adjusting for the local genetic context (Supplemental Text S10, S11). Consideration of the deletions observed clarifies this point. Although past literature featured a single microdeletion rate decreasing with size (Gu and Li 1995; Kondrashov 2003; Lynch 2010), sized-based rate variation cannot explain the aforementioned correspondence obtained for same-sized deletions, the higher rate of the Hb-Leiden mutation compared to the smaller deletions, or the extent of rate variation observed. Thus, the aforementioned correspondence, together with the fact that in these ROIs, the rates of some mutations (e.g., those of the HbS and Hb-Leiden mutations) deviate much more than others from their corresponding GWA rates show that mutation-specific rates vary not only in the case of large rearrangement mutations (Gu et al. 2008; Zhang et al. 2009) but also in the cases of point mutations and microindels. This rate variation could not have been seen using average-based measures (Kondrashov 2003; Lynch 2010) and establishes the relevance of mutation-specific point mutation and microindel rates to the site frequency spectrum (SFS) (Harpak et al. 2016; Lek et al. 2016; Mathieson and Reich 2017).

The overall point mutation rate in the *HBB* ROI is significantly higher in the African than in the European group even under a nonparametric comparison, which shows that the difference cannot be attributed to individual- or sample-level variation alone. Thus, it represents a significant population-level difference between the groups. This difference, occurring in an extremely narrow region spanning three codons of great importance for adaptation and genetic disease, is at least two orders of magnitude larger than previously reported differences in GWA mutation rates between continental groups (Harris 2015; Harris and Pritchard 2017). The correspondence between mutation-specific de novo rates and observations of alleles in carriers as well as this large difference in the overall point mutation rate between populations in a narrow region establish the importance of measuring mutation rate variation at an ultrahigh resolution.

Potential contributions to mutation rates from gross-level biological or environmental factors, such as age or pesticides, cannot sufficiently explain the results. First, the two populations are similar in ages (Supplemental Table S1). Second, any mutation-specific effect, like the correspondence between de novo rates and observations of alleles in carriers, cannot be explained by such macrolevel factors, because the latter cannot be expected to affect the rates of equivalent mutations, such as 20A→T in *HBB* versus *HBD*, differently. Third, the overall point mutation rate difference between the populations is also unlikely to be explained by them, because if on their own such macrolevel factors had affected the ROIs, they should have affected the entire genome similarly, yet GWA differences in point mutation rates between continental groups are smaller than the ROI-specific differences observed here (Harris 2015; Harris and Pritchard 2017). Note that if macrolevel factors affect mutation rates in interaction with mutation-, locus-, individual-, and/or population-specific factors, then such specific factors must be assumed in any case. Thus, rather than suggesting involvement of macrolevel factors, the data suggest a complex picture of mutation rates involving mutation-specific influences.

In addition, although the replication of mutations during spermatogenesis (clonal dependence) may make some contribution to the data, in practice it is insufficient to account for the significant results. First, the significance of the continental difference in the overall point mutation rates in *HBB* is impervious to any sample-level variation, including clonal dependence, as shown by the nonparametric between-population comparison described in the results section. Second, the correspondence between mutation rates and observations of alleles in carriers cannot be driven by it. On the contrary, in the absence of a cellular-level mechanism that induces specific mutations in a population-specific manner in accord with the cellular generation during spermatogenesis, differences in mutation timing during spermatogenesis could only add noise to the patterns observed, and thus any presence of clonal dependence would only make it more difficult to obtain significance for such patterns and in that sense is conservative to finding a pattern. Thus, more likely, the significance of these patterns is driven by independent originations of the mutations. These independent originations are consistent with mutation-specific rates being influenced by genetic and/or epigenetic factors (Livnat 2013, 2017).

The prevalence of a mutation of heterozygote advantage in a population and of reading-frame conservation in a coding sequence are generally considered to be outcomes of selection. However, here, both the HbS mutation, which provides strong malaria protection in heterozygotes, and the Hb-Leiden mutation, which is an in-frame deletion, are frequent not because of selection but because of frequent de novo origination. Indeed, that the rate of the in-frame Hb-Leiden mutation is much higher than that of all other observed deletions, which are frameshift deletions, shows reading-frame conservation that is not caused by selection (Lek et al. 2016) but rather by mutational phenomena. This observation provides a concrete example of “mutational conservation”—evolutionary conservation caused by mutational reasons which, if it occurs more broadly, could offer an explanation for the puzzling observation of reading-frame conservation bias in pseudogenes (Zhang and Gerstein 2003).

That the genetic sequences at and adjacent to the ROIs are identical for the two populations and for the two genes yet the mutation rates vary significantly between the populations and between the genes suggests that what affects these mutation rates in the germline includes more than this local DNA sequence and in that sense is complex (Livnat 2013, 2017). These results are consistent with the observation that the variation of the mutation rates across loci is partly cryptic (not explained by the local DNA context) (Hodgkinson et al. 2009; Hodgkinson and Eyre-Walker 2011), especially in the case of A↔T transversions (Hodgkinson et al. 2009), which include the HbS mutation type (A→T). Combining the multiple insights discussed, the results suggest that mutation rates are both mutation-specific and influenced in a complex manner by the genetic and/or epigenetic background (Livnat 2013, 2017).

The *HBB* region spanning three codons is of particular importance for adaptation and genetic disease: it is the site of mutations that provide strong protection against malaria (HbS and HbC, the latter not observable by our method) and/or increase the risk for hematologic disease (Flint et al. 1998; Hardison et al. 2002; Hardison and Miller 2002). Thus, it is of interest that the overall point mutation rate in this region is significantly higher than expected, and that it is significantly higher in the African than in the European population. These results provide a clear case of a connection between mutation rates and adaptive evolution, thus moving beyond previous literature on the relevance of mutation rates to adaptive evolution and its repeatability (Crow et al. 2009; Dumas et al. 2012; Kratochwil et al. 2019; Kratochwil and Meyer 2019; Lind 2019; Xie et al. 2019).

The results underscore the importance of mapping the mutation rate variation at an ultrahigh resolution. It is beyond this fact that several observations on the HbS mutation specifically can be mentioned. First, if one assumes that the HbS rate is the same for both of the continental groups, the data show that it is significantly higher by nearly 20-fold than expected from the GWA for this mutation type, in both Africans and Europeans. Any amount of hypothetical clonal dependence does not change this estimate of the observed evolutionarily relevant mutation rate, because the latter does not depend on the cause of the recurrence of the mutation in the sperm. Even the observed minimal rate of independent HbS originations in sperm is still significantly larger by 6.5× than the evolutionarily relevant GWA rate for this mutation type. Consideration of the local genetic context does not change this conclusion (Supplemental Text S10). Thus, although the classical explanation of the HbS case relied only on selection, even under the most conservative assumptions the overall HbS mutation rate observed here is notably higher than expected.

Second, given the significant continental difference in the overall point mutation rate between the groups, it would be surprising if the HbS mutation specifically does not show a continental effect. Consistent with this, in our samples, using the methodology described, we observe no instances of it in Europeans but nine instances of it in total in Africans, amounting to a rate ∼35× higher than expected from the GWA of this mutation type in the latter. Further consistent with a continental difference in the HbS mutation rate, it fits with the broader correspondence between de novo rates and observations of alleles in populations that HbS is most frequent in Africans and in some other populations in the Asian malaria belt (Flint et al. 1998) and appears de novo in our African but not in our European samples, whereas Hb-Leiden has been observed across the globe (Hardison et al. 2002; Hardison and Miller 2002) and appears de novo in both our African and European samples.

Third, in the African *HBB* ROI, out of 12 observable point mutations, the HbS mutation has the rate that deviates the most from the corresponding GWA rate (Supplemental Table S4).

Fourth, it is striking that despite at least three independent occurrences of the HbS mutation in the *HBB* ROI, not a single case of the equivalent 20A→T mutation in the *HBD* ROI was observed in any donor, African or European. Accordingly, we note that the binary test establishing the significantly higher concentration of the 20A→T mutation in the African *HBB* ROI as opposed to all other cases (the European *HBB* ROI or the *HBD* ROIs), which is impervious to any individual- or sample-level variance including clonal dependence, suggests that the 20A→T mutation arises more frequently where it is of adaptive significance than where it is not, although data do not suffice to tell whether this effect results from a population-level difference or from a locus-based difference or from both.

Knowing that the HbS mutation is advantageous in heterozygotes under malarial pressure, how should we interpret these results? One possibility is that, for a reason unrelated to adaptation, some individuals have a genomic fragility in *HBB* that generates the HbS mutation at a high rate. Accordingly, it is merely a coincidence that HbS provides protection against malaria, even more so if that fragility applies more to Africans.

Another possibility is modifier theory (Feldman and Liberman 1986; Altenberg et al. 2017), according to which alleles affecting the mutation rate may be favored by selection under certain conditions (Leigh 1970; Moxon et al. 1994). However, because the benefit of a modifier allele that increases the mutation rate is tied to the excess beneficial mutations it helps to generate, and because mutations are rare, it is normally expected that, for selection to be effective, it must act on a modifier allele that increases the mutation rate across a long enough stretch of the genome with which it remains linked for a long enough period of time, so that many different mutations potentially induced by this allele over space and time are factored into its selective benefit (Hodgkinson and Eyre-Walker 2011; Martincorena and Luscombe 2013; Walsh and Lynch 2018). Thus, modifier theory does not predict an increase in the rate of particular DNA mutations at specific base positions, let alone in sexual, complex organisms, nor the complex genetic and/or epigenetic influences on such mutation rates suggested by the current data (cf. Leigh 1970; Moxon et al. 1994; Altenberg et al. 2017; Walsh and Lynch 2018). On the contrary, the “reduction principle”—the first-order principle in modifier theory—underscores the general difficulty of accounting for increased mutation rates (Feldman and Liberman 1986; Altenberg et al. 2017).

Finally, a recently proposed theory predicted that mutation-specific origination rates are influenced by the complex genetic and epigenetic background, that genetic relatedness in mutational tendencies exist, and that the HbS mutation arises more frequently in Africans than in Europeans (Livnat 2013, 2017). It holds that novelty in evolution arises from emergent interactions, which are then simplified through the generations by mutational mechanisms while being checked by natural selection (Livnat 2017), one hypothetical example being that A→I RNA editing can mechanistically increase the A→G mutation rate in the corresponding positions (cf. Popitsch et al. 2020). Based on these and other previous work (Livnat and Papadimitriou 2016), we hypothesize that recurring, evolved processes acting on DNA and/or RNA through epigenetic modifications (Klose and Bird 2006), RNA editing (Nishikura 2010) and other mechanisms may lead directly to their own replacement and simplification via DNA mutations that arise in the course of evolution from these processes’ molecular nature, mechanistically linking regulatory activity with structural mutational changes—although whether and by what specific mechanism this “replacement” hypothesis explains the HbS case specifically (alternative decoding of A→I editing [Licht et al. 2019] or other mechanisms) is yet to be investigated. This raises the possibility that a mutation of adaptive value such as the HbS one need not initiate the process of adaptation but can arise later in an evolutionary process where adaptations and mutation-specific rates jointly evolve (Livnat 2013, 2017), and thus studies on the fundamental nature of mutation need to test for not only a short-term response to environmental pressures (Luria and Delbrück 1943; Cairns et al. 1988) but also a long-term one.

Unlike previous methods that could explore only diffuse relationships between long-term selection pressures and the evolution of GWA mutation rates, the present method offers the refined ability needed to explore such relationships, if they exist, at the mutation-specific resolution. Because this method examines the mutation-specific resolution for the first time, it provides only initial estimates of mutation rates, which will require further investigation and refinement. Furthermore, it cannot be applied currently to all mutations, because it requires a special RE for each ROI. However, given the numerous REs available and their short recognition sequences, which imply large representation of these sequences across the genome, it likely applies across many loci and organisms. Therefore, some of the most important tasks now are to examine the high-resolution mutation rate variation across additional loci of interest and to explore the molecular mechanisms responsible.

## Methods

For the experimental design and different stages of library preparation, see Supplemental Text S1–S3 and Supplemental Figures S1–S3. All of the oligos for the sperm DNA library preparation described in Supplemental Text S14 were ordered from Integrated DNA Technologies (IDT) with standard desalting purity, unless otherwise mentioned. All enzymes were obtained from New England Biolabs (NEB). Plasmid mini-prep, PCR purification, and agarose gel extraction were performed with QIAGEN kits.

### Spike-in plasmid preparation

Four puc19-based plasmids were generated. Two (ALP13 and ALP17) were designed to carry the *HBB* genomic segment from position −203 to +223 relative to the mRNA translation start site, with the Bsu36I restriction site CCTGAGG replaced with TTATGTT and ACGAGAC, respectively; and two others (ALP16 and ALP18) were designed to carry the *HBD* genomic segment from position −59 to +220 relative to the mRNA translation start site, with the Bsu36I restriction site replaced with TTATGTT and ACGAGAC, respectively. To prepare the spike-in mixture, the four plasmids were linearized by BamHI, mixed in equal amounts, and diluted to 10 fg/µL for the AFR1, AFR3, AFR5, AFR6, AFR7, EUR3, and EUR4 samples and to 5 fg/µL for all other samples.

### Collection of sperm samples

Semen samples from Africans were collected in the Assisted Conception Unit of the Lister Hospital and Fertility Centre in Accra, Ghana, following clinical standards. Semen samples from Europeans were purchased from Fairfax, a large US cryobank, with the approvals of the Institutional Review Board of the Noguchi Memorial Institute for Medical Research (NMIMR-IRB 081/16-17) at the University of Ghana, Legon, the Rambam Health Care Center Helsinki Committee, Haifa (0312-16-RMB), and the Israel Ministry of Health (20188768). Donors with a history of cancer or infertility or with high fever in the 3 mo before donation were excluded. Informed consent was obtained from all participants, and personal identifying information was removed and replaced with codes at the source.

### DNA extraction from sperm cells

The DNA isolation protocol was modified from Weyrich (2012). A semen sample from a single donor was divided into 500-µL aliquots in multiple screw-capped tubes. The sperm aliquots were washed twice with 70% ethanol to remove seminal plasma. The remaining cells were rotated overnight at 50°C in a 700-µL lysis buffer (50 mM Tris-HCl [pH 8.0], 100 mM NaCl2, 50 mM EDTA, 1% SDS) containing 0.5% Triton X-100 (Fisher BioReagents BP151-100), 50 mM Tris(2-carboxyethyl) phosphine hydrochloride (TCEP; Sigma-Aldrich 646547), and 1.75 mg/mL Proteinase K (Fisher BioReagents BP1700-100). Lysates were centrifuged at 21,000*g* for 10 min at room temperature, and the supernatants were united in a single tube. DNA purification from the cleared lysate was performed using QIAGEN Blood and Cell Culture DNA Maxi Kit (13362). Specifically, 5 mL lysate were supplemented by 15 mL buffer G2 (800 mM guanidine hydrochloride, 30 mM Tris-HCl [pH 8.0], 30 mM EDTA [pH 8.0], 5% Tween 20, 0.5% Triton X-100), vortexed thoroughly, and allowed to gravity flow through a single Genomic-tip 500/G column pre-equilibrated by 10 mL buffer QBT (750 mM NaCl, 50 mM MOPS [pH 7.0], 15% isopropanol [v/v]). Resin was washed twice by 15 mL Buffer QC (1 M NaCl, 50 mM MOPS [pH 7.0], 15% isopropanol [v/v]), and elution was performed by 15 mL Buffer QF prewarmed to 50°C (1.25 M NaCl, 50 mM Tris-HCl [pH 7.0], 15% isopropanol [v/v]). DNA was precipitated by adding 10.5 mL room temperature isopropanol to the elute, inverting the tube 10 times, and using a sterile tip to spool and transfer the DNA to a screw-capped tube containing 500 µL buffer EB (10 mM Tris-HCl [pH 8.5]). The DNA was allowed to dissolve overnight at room temperature. For each donor, a small aliquot from the extracted DNA was PCR amplified and Sanger sequenced to verify the exact sequence of the *HBB* and *HBD* regions and to confirm that the donors were homozygous for the WT sequence for both ROIs.

### Enzymatic digestion

For the Bsu36I-treated sample (Supplemental Text S1–S3), ∼264 µg sperm DNA, equivalent to 80 million haploid cells (for AFR2, a DNA amount equivalent to 60 million cells was used), were mixed with a plasmid spike-in mixture (0.2 pg for AFR1 and 0.1 pg for other donors) and equally divided in a 96-well plate. Bsu36I digestion was performed overnight at 37°C according to the manufacturer's instructions using 5 units per well. Then, each well was supplemented by 6 units of HpyCH4III to generate the primary barcode attachment site, and digestion continued for an additional 3 h. For the Bsu36I-untreated reaction, 13.2 µg sperm DNA (and 9.9 µg for AFR2), representing 5% of the DNA amount used for the Bsu36I digest, were mixed with 6 times the volume of plasmid spike-in mixture, aliquoted to five tubes, and incubated overnight with 2 units SalI-HF per tube instead of Bsu36I to allow for similar conditions of DNA digestion without affecting the Bsu36I and HpyCH4III sites. Then, each well was supplemented by 6 units of HpyCH4III and digestion continued for an additional 3 h, followed by DNA purification.

### Primary barcode labeling and linear amplification

Direct barcode labeling and linear amplification of the digested *HBB* and *HBD* strands were performed in a single reaction in 96-well plates. Each well contained ∼1 µg of digested DNA, 0.1 µM primary barcode oligo (oligo A) (Supplemental Text S14), and 1 µM of 5′-phosphorothioate-protected primer for linear amplification (oligo B). The reaction was performed with Q5 high-fidelity polymerase according to the manufacturer's instructions, using the following thermocycler parameters: initial denaturation for 20 sec at 98°C, followed by 16 cycles for 5 sec at 98°C, for 15 sec at 68°C, and for 20 sec at 72°C. For each donor, each of the Bsu36I-treated and -untreated samples was labeled by an oligo A with a different Donor Identifier-1 (ID-1) sequence, which was also not shared by samples from other donors, providing each donor and each condition with a unique identifier sequence.

### 5′-exonuclease treatment

To eliminate non 5′-phosphorothioate-protected strands, following purification, 15 µg DNA aliquots from the post-linearly amplified product of the Bsu36I-treated sample were incubated each at 37°C in the presence of 15 units of Lambda exonuclease, 30 units of T7 exonuclease, and 90 units of RecJF exonuclease in 1× CutSmart buffer for 2.5 h. The post-linearly amplified product of the Bsu36I-untreated sample was incubated at the same conditions with 10 units of Lambda exonuclease, 20 units of T7 exonuclease, and 60 units of RecJF exonuclease.

### Secondary barcode labeling and 3′-exonuclease treatment

Following purification, the DNA was aliquoted into a 96-well plate (1 µg per well). A single primer extension reaction was performed using 0.5 µM of the secondary barcode primer (oligo C) and Q5 high-fidelity polymerase according to the manufacturer's instructions. The following thermocycler parameters were used: initial denaturation for 20 sec at 98°C, followed by a single cycle for 5 sec at 98°C, for 15 sec at 68°C, and for 40 sec at 72°C. To remove excess oligo C, immediately after the thermocycler temperature dropped to 16°C, 20 units of thermolabile Exo I were added directly to each well together with the relabeling control primer (oligo D) in a known amount equivalent to 0.66% of the secondary barcode primer. After incubation of 1 h at 37°C, the thermolabile Exo I was heat-inactivated for 1 min at 80°C and the DNA was purified. For each donor, each of the Bsu36I-treated and -untreated samples was labeled by an oligo C with a different Donor Identifier-2 sequence (ID-2), which was also not shared by samples from other donors, resulting in each donor and each condition having a unique Identifier-2 sequence.

### PCR amplification and sequencing

The first PCR reaction of the dual-barcode-labeled product was performed using oligo E and oligo F1 as primers and Q5 high-fidelity polymerase, according to the manufacturer's instructions. The following thermocycler parameters were used: initial denaturation for 30 sec at 98°C, followed by 10 cycles for 5 sec at 98°C, for 15 sec at 72°C, for 30 sec at 72°C, and a final extension for 30 sec at 72°C. Amplification products were purified, and the second PCR reaction was performed using 25% of the first PCR product as template, the amplification primers E and F2, and Q5 high-fidelity polymerase according to the manufacturer's instructions (different F2 primers were used to add a unique Illumina index sequence to each Bsu36I-treated and -untreated sample). The following thermocycler parameters were used: initial denaturation for 30 sec at 98°C, followed by 24 cycles (with the exception of the EUR4 sample that was amplified by 17 cycles) for 5 sec at 98°C, for 15 sec at 70°C, for 30 sec at 72°C, and a final extension for 1 min at 72°C. PCR products were agarose gel purified and further concentrated by a DNA clean and concentrator kit (Zymo Research). DNA libraries prepared from the Bsu36I-treated and -untreated samples of the same donor were mixed in equal amounts and paired-end sequenced with 20% PhiX by Illumina MiSeq 300 cycles kit (V2) at the Technion Genome Center (TGC). For each donor, two or three MiSeq runs were performed to reach a minimum of 10 million reads per treatment (specifically, all but AFR5 and EUR3 were sequenced two times), and the resulting FASTQ sequences were joined before the sequence analysis step.

### Sequence analysis

Illumina paired-end (PE) reads were merged via Pear (Zhang et al. 2014) using the default model for the detection of significantly aligned regions and Phred score corrections. Merged sequences were trimmed from Illumina adapters using cutadapt (Martin 2011), and quality filtered by Trimmomatic (Bolger et al. 2014) using a sliding window size of 3 and a Phred quality threshold of 30. Quality filtered sequences were trimmed to remove the 5′ edge up to position 18, a sequence which includes the 14 bases of the primary barcode and the 4 bases of ID-1, while adding this information to the read's header. Only sequences with the correct ID-1 and first three bases of *HBB* or *HBD* sequences were maintained. Similarly, sequences were trimmed from 9 bp at their 3′ edge, which include the 5 bases of the secondary barcode and the 4 bases of ID-2, while adding this information to the read's header. Only sequences with the correct ID-2 were maintained. Trimmed sequences were sorted to *HBB* or *HBD* sequence pools, based on the occupying bases at positions 33–38 of the coding sequence (CGTTAC for *HBB* and TGTCAA for *HBD*), allowing one mismatch and frameshifts of up to −3 or +3. Successfully sorted sequences were mapped to either the *HBB* or *HBD* reference sequence (obtained by Sanger sequencing aliquots from the matching donor samples) using BWA (Li 2013) (parameters -M -t), and high-quality mutations (Phred score ≥28) were noted. Reads were grouped by their primary barcodes to “families” and processed according to the workflow depicted in Supplemental Figure S9.

## Data access

All raw sequencing data generated in this study have been submitted to the NCBI database of Genotypes and Phenotypes (dbGaP; https://www.ncbi.nlm.nih.gov/gap/) under accession number phs002391.v1.p1. For final processed data see Supplemental Datasheets and Supplemental Text S15. Software is available at GitHub (https://github.com/livnat-lab/HBB_HBD) and as Supplemental Code.

## Supplementary Material

Supplemental Material
