# Supplementary material for: De novo mutation rates at the single-mutation resolution in a human HBB gene region associated with adaptation and genetic disease
Source: Genome Res. 2022 Mar;32(3):488–98. doi: 10.1101/gr.276103.121 (PMC8896469; doi:10.1101/gr.276103.121)
Supplement: Supplemental Material [file supp_32_3_488__DC1.html]

De novo mutation rates at the single-mutation resolution in a human HBB gene region associated with adaptation and genetic disease — De novo mutation rates at the single-mutation resolution in a human HBB gene region associated with adaptation and genetic disease — Supplemental Material 

# De novo mutation rates at the single-mutation resolution in a human *HBB* gene region associated with adaptation and genetic disease

## Supplemental Material

- Supplementary\_Datasheets.zip
- Supplemental\_Material.pdf
- SUPPLEMENTAL\_v1.1.zip
