# Supplementary material for: De novo mutation rates at the single-mutation resolution in a human HBB gene region associated with adaptation and genetic disease
Source: Genome Res. 2022 Mar;32(3):488–98. doi: 10.1101/gr.276103.121 (PMC8896469; doi:10.1101/gr.276103.121)
Supplement: Supplemental Material [file supp_gr.276103.121_Supplemental_Material.pdf]

Supplemental Material

De novo mutation rates at the single-mutation resolution in a human *HBB* gene-region associated with adaptation and genetic disease

Daniel Melamed, Yuval Nov, Assaf Malik Michael B. Yakass, Evgeni Bolotin, Revital Shemer, Edem K. Hiadzi, Karl L. Skorecki, Adi Livnat*

* To whom correspondence should be addressed; E-mail: alivnat@univ.haifa.ac.il

This PDF file includes the below:

Supplemental Text S1 to S15

Figs. S1 to S14

Tables S1 to S4

Supplemental References

Other supplemental materials for this manuscript include the below:

Supplemental Datasheets SD1 to SD44

1. The MEMDS method

1.1. Current high-accuracy sequencing methods

While High-Throughput Sequencing (HTS) technology has improved tremendously the cost and scale of DNA sequencing, the detection of extremely rare genetic variants remains a major challenge. This unresolved problem is due both to DNA polymerase errors that are introduced during sample preparation and to sequencing errors made by the HTS machinery. DNA polymerase error rates range from $\sim 1 \times 10^{-4}$ per base for Taq polymerase to $\sim 1 \times 10^{-6}$ per base for various high-fidelity DNA polymerases (Hestand et al., 2016; Lee et al., 2016; Potapov and Ong, 2017). Error rates of the commonly used HTS platforms range from 10^{-2} to 10^{-3} per sequenced base (Fox et al., 2014), with some computational efforts being able to enhance sequencing accuracy 10–100 fold (Ma et al., 2019; Cibulskis et al., 2013). These abilities allow for the detection of some sub-populations of sequences within a highly homogenous sample. However, given that the average per base point mutation rate across the human genome is $\sim 10^{-8}$ (see Supplemental Section 10), entailing on average $\sim 10^{8}$ wild-type copies per mutant, the rates above would lead to numerous false positives per each true base mutation. Thus, detecting a single instance of a particular *de novo* mutation in a particular gene has been practically impossible so far. Moreover, even in the absence of errors, obtaining enough reads of such a mutation by sequencing alone would have entailed exorbitant sequencing costs.

In recent years, a few experimental approaches have been developed that substantially reduce the noise generated by both DNA polymerase and HTS errors (Casbon et al., 2011; Kinde et al., 2011; Hiatt et al., 2013; Lou et al., 2013; Hong and Gresham, 2017; Gregory et al., 2016; Schmitt et al., 2012; Wang et al., 2016; Hoang et al., 2016; Jee et al., 2016). One key idea is to attach a unique molecular tag, or "barcode," to each DNA fragment at the first PCR cycle of the amplification step. After library preparation and standard high-throughput sequencing, reads that share the same barcode are recognized as having been derived from the same original

molecule. Since those reads should be identical, the differences between them are considered to be errors introduced during PCR and/or HTS and are filtered out at the computational analysis step (Kinde et al., 2011). This filtration step removes many of the DNA polymerase and HTS errors that occur after barcode attachment.

Importantly, however, the standard way by which the barcode has been added to the target DNA is by being included as a part of a target-specific primer that is extended by a single elongation reaction, generating a sequence subsequently to be amplified using an external pair of primers. A major disadvantage of this standard method is that any replication error introduced by the DNA polymerase during the critical, initial copying of the original DNA molecule is transferred to all downstream copies during the PCR reaction and cannot be filtered out by the regular barcoding-and-consensus-sequencing approach.

To overcome this problem, a few methods have been developed. In Duplex Sequencing (DS) (Schmitt et al., 2012), double-stranded barcodes are attached to both ends of a sheared DNA segment by ligation. This operation makes it possible, at the sequence analysis step, to group together the two sets of copies from the two strands of each original double-stranded DNA molecule based on their barcode complementarity. A consensus sequence is first constructed for each strand of an original double-stranded DNA molecule from its set of copies, and then the two consensus sequences are compared to each other. The identity of a base at each position is approved only if the two consensus sequences show a perfect match. This approach allows DS to capture errors that occur at any amplification and sequencing step, including the generation of the first copy of each original molecule, and to reach an error rate below $\sim 2.5 \times 10^{-6}$ when applied to the M13mp2 bacteriophage DNA (Schmitt et al., 2012) and an error rate of 1×10^{-7}–5×10^{-8} according to unpublished data (Kennedy et al., 2014). Most recently, this accuracy has been improved further in the context of sequencing large parts of the genome (Abascal et al., 2021). However, while ideal for genomic regions at the 1Mb scale, used on smaller

regions, the hybridization-based capture that it uses as a consequence of the attachment of duplex barcodes by ligation to double-stranded molecules would entail extremely low yields, which in turn would make it impossible to reduce the size of the region of interest (ROI) and increase the sequencing depth in a manner cost-effective enough as to focus on a particular mutation of interest.

An alternative method that avoids errors in the first copying step, Maximum Depth Sequencing (MDS) (Jee et al., 2016), is based on single-strand sequencing. However, instead of generating a barcoded copy of each original DNA molecule by extending a barcoded primer, it adds the barcode to the original target DNA molecule by cleavage of the target molecule near the ROI followed by a fill-in reaction that extends the target-DNA strand using a barcoding oligo as a template. Next, linear amplification is performed to obtain multiple copies of the target molecule, each generated directly from the original, now barcoded, single-stranded target DNA molecule. The preparation of the library is then completed by a standard exponential PCR.

Like DS, MDS consensus sequencing reaches all the way to the very original target DNA molecules, without an intervening, uncontrolled copying step. However, in contrast to DS, MDS can potentially minimize the ROI to a single base in the genome. Since MDS recovers the sequence information from only one of the two DNA strands, however, it cannot correct certain kinds of error due to DNA damage or base misincorporation by the cellular DNA polymerase that affect the target DNA strand, in contrast to DS. Yet, eliminating these highly frequent, known types of errors from the mutation rate calculation resulted in a tested MDS error rate of about 1×10^{-7} while using Phusion DNA polymerase, and a suggested theoretical error rate of less than 5×10^{-8} if Q5 DNA polymerase is used (Jee et al., 2016).

1.2. MEMDS boosts both mutation detection accuracy and yield

While both DS and MDS reach extraordinary levels of precision, their error rates and sequence coverage demands still pose serious difficulties in detecting particular mutations occurring at or near the human genome-wide average mutation rate. In particular, note that natural mutation variants constitute a tiny fraction of the target DNA molecules, while the vast majority of the target DNA consists of a common, non-mutated sequence, which we refer to as the "wild-type" sequence. This fact has two negative consequences. First, since each wild-type molecule is one that can be mistakenly read as a mutation, the wild type is a ubiquitous source of false-positives. Second, since our goal is to detect mutations, most of the sequencing capacity and costs are spent on sequence copies that are of little interest. Therefore, removing as many wild-type sequences as possible prior to DNA sequencing, *while measuring the extent of that removal*, would greatly improve both accuracy and sequencing efforts.

We present here a method, named MEMDS (Mutation Enrichment followed by upscaled Maximum Depth Sequencing), which uses principles of MDS for barcoding, as described above, but reaches a notably higher accuracy at a much smaller cost while focusing on detecting mutations in a very narrow ROI. MEMDS enriches the sample for mutations in the ROI prior to library preparation by removing a large fraction of non-mutated variants. In addition, it includes various steps that *a*) further enable the processing of the very large initial amounts of genomic DNA, as required for identifying *de novo* mutations in humans; *b*) enhance accuracy by using routinely and in an informed manner a dual barcoding system and other measures guarantying the authenticity of target DNA molecules; and *c*) accurately quantify the fraction of non-mutated variants removed, which is necessary in order to obtain the denominator for the calculation of *de novo* mutation rates. Using MEMDS, we achieve an error rate of at least 2.5×10^{-9} per base after removing the high-frequency G→T, C→T and C→A mutations (see Supplemental Section 8) and a recovery rate of $\sim$35% of the input target sequences due to

normal loss of material. With this recovery rate, for example, starting with 3 instances of a particular mutation in 300 million cells, 1 mutation in 100 million cells on average could be identified and reported. Thus, the recovery rate only affects the cost of sampling, and does not affect the cost or the accuracy of sequencing.

1.3. The MEMDS method outline

MEMDS involves two workflows that are run in parallel. One enriches for mutations in the ROI. In this work, enrichment is performed using restriction enzyme digestion, though alternatives like CRISPR-editing (Jinek et al., 2012) could also be used depending on the types of mutations examined (point mutations, indels) and improvements in site-recognition specificity (Tsai and Joung, 2016). The other workflow is used for computing the enrichment fold, and hence the exact number of wild-type ROI sequences that have been removed from the ROI pool. The protocol outlined below and in Fig. S1 describes the workflow for the enrichment of mutated ROIs, and is identical to the one used for computing the enrichment fold, with the exception that the restriction enzyme used for enrichment (Fig. S1, step 1) is omitted in the latter. For a detailed explanation of the complete experimental design involving the two workflows, see Supplemental Section 2.

Step 1: Enzymatic digestion of genomic DNA: The genomic DNA is digested by two restriction enzymes. The first (RE-1) digests the wild-type sequence at a certain site that is several residues long and that constitutes the region of interest (ROI). Namely, the experiment is designed by choosing an ROI and an RE-1 so that the recognition site for RE-1 matches the wild-type sequence at the ROI. As a result, sequences with no mutations are efficiently digested, while variants with mutations that hamper site recognition by RE-1 are protected from cleavage. Therefore, these mutations are enriched in the pool of uncleaved sequences (for calculating the exact number of wild-type ROIs that have been removed by RE-1, see the

complete experimental design in Supplemental Section 2 and Fig. S2). The second restriction enzyme (RE-2) is used to cleave the DNA near the ROI. The choice of a suitable RE-2 is dependent on the availability of an adequate recognition site far enough from the RE-1 site to allow for an efficient annealing of a primary barcode oligo (oligo A) between the two sites, yet short enough to meet the read-length limits of the chosen HTS platform. To satisfy these conditions, the RE-2 site may be selected to be either upstream or downstream of the ROI, a choice which will determine which of the two DNA strands will be barcoded and analyzed.

Step 2: Primary barcode attachment: Following digestion, the DNA is subjected to single-strand extension using a high-fidelity DNA polymerase and a single oligonucleotide (oligo A). Oligo A anneals with its 3' part to the sequence between the RE-2 site and the RE-1 site and acts as a template for extension of the target-DNA strand. This extension reaction introduces three sequence features directly into the target strand: *a*) a segment of four bases that serves as a sample-identifier sequence to secure the sample in the event of a rare contamination by DNA libraries from other samples; *b*) 14 randomized bases that create a primary barcode unique to each particular DNA fragment; and *c*) an Illumina P5-primer sequence. In order to prevent the oligonucleotide itself from being extended while using an already barcoded target strand as template in the subsequent linear amplification step, an inverted-dT modification is included at the 3' terminus of oligo A that blocks the DNA polymerase and prevents the extension of oligo A during the process. To account for the event that some oligo A molecules escaped the inverted-dT modification during their synthesis by the manufacturer, a single-base insertion is planted in the oligo A sequence that anneals to the genomic strand, so that undesired extensions of rare, unblocked oligos could be easily detected at the sequence analysis step for their inclusion of this single-base insertion, and be removed.

Step 3: Linear amplification of barcoded ROI products: The genomic ROI is linearly amplified by 15 cycles using a high-fidelity DNA polymerase and a single primer (oligo B) that

anneals to the Illumina P5-primer sequence. Oligo B contains the complete Illumina-adapter sequence, and carries five phosphorothioate bonds (PS) at its 5' edge. This step results in up to 15 single-stranded copies of each barcoded ROI (each copy having been generated directly from the same barcoded original DNA molecule), protected by the phosphorothioate bonds from 5'-exonuclease activity.

Step 4: Degradation by 5'-exonucleases: The linear amplification products are treated with a mixture of 5'-exonucleases, which degrade both single and double-stranded DNA with or without phosphate groups at their 5' termini, from the 5' edge to the 3' edge of each strand. The linearly-amplified ROI copies are protected from this exonuclease activity due to the multiple PS bonds at their 5' edges. This step removes the majority of the genomic DNA, including most of the ROI digestion products, and simplifies the rest of the experimental workflow by allowing the next reactions to be carried out in a small number of tubes rather than in 96-wells plates, as well as by eliminating sequences that could potentially promote the generation of unwanted byproducts in the subsequent amplification steps.

Step 5: Secondary barcode attachment: The DNA from the 5'-exonuclease reaction is subjected to a single primer-extension reaction, using a secondary barcode primer (oligo C) that anneals 3' to the ROI site and is extended by a single cycle using a high-fidelity polymerase. The secondary barcode primer also has three features: *a*) a segment of four bases that serves as a sample-identifier sequence; *b*) five randomized bases that create a secondary barcode generally unique to each member within a family of copies (copies sharing the same original DNA molecule); and *c*) an Illumina P7-primer sequence. This step produces a complementary strand for each of the 15 copies (or less) generated per target DNA molecule during the linear amplification step. Each of these complementary strands carries the same primary barcode sequence and a unique secondary barcode sequence.

Step 6: Degradation by a 3'-exonuclease: To prevent recurrent labeling by secondary

barcode primers in subsequent amplification reactions, a 3'-exonuclease that degrades single-stranded DNA from the 3' edge to the 5' edge of the molecule is added immediately after the secondary barcode attachment to eliminate free, unbound primers. The double-stranded molecules that just completed the secondary barcode extension reaction are protected from this degradation. The 3'-exonuclease is added together with a known amount of relabeling-control primer (oligo D). This control primer is identical in sequence to the secondary barcode primers except for the sample-identifier and the secondary barcode features that are replaced by a known sequence. Therefore, in the event of incomplete degradation by the 3'-exonuclease, the amount of HTS reads with an oligo D sequence signature serves as a proxy for the frequency of relabeling by the secondary barcode primer.

Step 7: Amplicon generation by PCR for next generation sequencing: PCR amplification of the purified DNA is carried out using primers E and F, which add Illumina index and adapter sequences to the 3' edge of the amplicon (as described in the methods section, we break this step into two PCR reactions to preserve some of the first PCR product as a backup). Importantly, RE-1 digestion products that have not been eliminated until this step will not be amplified, as only complete segments that have not been digested by RE-1 have the two primer annealing sites.

Step 8: Analysis of sequenced data: HTS reads are grouped into families based on their primary barcode sequences. Thus, each family is made of a collection of sequences originated from linearly amplified copies of a single target DNA strand, belonging to a single gene. Each read in a family is aligned against a reference sequence specific to the donor, and mutations with a high-quality sequencing score are noted. Three criteria are then used in combination to select for true mutations: *a*) the number of reads in the family (i.e., family size); *b*) the number of secondary barcodes associated with a particular mutation (i.e., BC2 count); and *c*), the fraction of the particular mutation in the family (i.e., mutation frequency). Mutation candidates that

pass the combined cutoff criteria are designated as true, *de novo* mutations. The total number of target wild-type sequences screened, which consist of *a*) target wild-type sequences that have been digested by RE-1 and removed from the final DNA libraries, and *b*) target wild-type sequences that evaded RE-1 digestion and were included in the sequenced DNA libraries, is calculated from the sequencing outputs of the RE-1–treated and the RE-1–untreated samples (see Supplemental Section 2 and Fig. S2 for a detailed description of this calculation). Finally, from the mutation count and the total number of cells scanned, we calculate the per locus, per mutation *de novo* mutation rate for mutations of interest in the ROI.

Note that, like other barcode-based systems, MEMDS is not sensitive to the usual stochastic skewing of allelic balance during PCR, because the barcoding enables collapsing all reads originating from a single target molecule into one.

2. Experimental design

2.1 Calculating the number of RE-1 digested sequences

The mutations studied here are not expected to affect sperm viability and fertility. Under the assumption that a given mutation is not associated with enhanced or reduced sperm viability or fertility, dividing the number of cells carrying that mutation by the total number of cells scanned gives the probability of the male transmitting this mutation to the next generation and is therefore the evolutionarily relevant *de novo* mutation rate for this mutation in males.

The number of target sequences scanned by the MEMDS procedure, each originating from a unique cell, includes two sets of molecules: *a*) target sequences identified at the sequence analysis step; and *b*) target sequences removed by the enrichment step described in section 1.3 (Fig. S1, step 1). Therefore, to calculate the mutation rate, one must be able to determine how many target wild-type sequences have been removed by RE-1 digestion as opposed to having been removed by general loss of genetic material during the MEMDS procedure. The fold-

reduction in target wild-type sequences, also referred to here as the RE-1 enrichment fold for RE-1–resistant mutations, multiplied by the number of target wild-type sequences identified at the sequence analysis step, yields essentially the number of target sequences scanned by MEMDS. This number, in turn, serves as the denominator in the mutation rate calculation.

Toward this end, we have established an experimental design that uses the HTS output to obtain a precise measurement of the RE-1 enrichment fold. This experimental design avoids errors due to impreciseness of input DNA concentration measurements as well as variation in DNA loss and in performance of the MEMDS steps across samples.

We start with two tubes: a genomic- and a mock-DNA tube (Fig S2). The genomic-DNA tube includes the DNA extracted from the human sperm sample. In people who are not carriers of HbS or other mutations in the ROI, this tube contains mostly wild-type target sequences, which are sensitive to digestion by RE-1, and which are denoted S. The other tube is a mock-DNA tube containing copies of an artificial sequence, denoted R, which are resistant to RE-1 digestion and are easily distinguishable from natural mutants at the sequence analysis step. From the genomic-DNA tube, we transfer an amount of material into an "RE-1–treated" tube (Fig S2), whose material will undergo the full protocol including RE-1 digestion, and another amount into an "RE-1–untreated" tube, whose material will undergo the same steps except for digestion by RE-1. Likewise, from the mock-DNA tube, we transfer an amount of material into the RE-1–treated tube and another amount into the RE-1–untreated tube. The principle underlying this design is that the relative amounts transferred from a tube can be known through their volume measurements alone and, given these measurements, the RE-1 enrichment fold can be obtained by comparing the ratios between the numbers of sensitive and resistant DNA molecules following each treatment (as shown formally below), where these amounts are precisely known from the sequence analysis step.

Specifically, let the concentrations of S in the genomic-DNA tube and of R in the mock-

DNA tube be $[S]$ and $[R]$, respectively. From the genomic-DNA tube we move a volume V_{S_e} to the "RE-1–treated" tube and a volume V_{S_c} to the "RE-1–untreated" tube. From the mock-DNA tube we move a volume V_{R_e} to the RE-1–treated tube and a volume V_{R_c} to the RE-1–untreated tube. Let L_e represent the fold loss of material (whether sensitive or resistant) due to normal loss in the RE-1–treated condition, and L_c represent the fold loss of material due to normal loss in the RE-1–untreated condition. Finally, let E be the RE-1 enrichment factor (i.e., $1/E$ is the fold reduction in sensitive molecules in the RE-1–treated tube due to RE-1 digestion). At the final, sequence analysis step, we can precisely count the number of sensitive (i.e., wild-type) molecules called in the RE-1–treated condition, S_e^f; the number of artificial, resistant molecules called in the RE-1–treated condition, R_e^f; the number of sensitive (i.e., wild-type) molecules called in the RE-1–untreated condition, S_c^f; and the number of resistant molecules called in the RE-1–untreated condition, R_c^f. These quantities can be written as follows:

$$S_e^f = [S] \cdot V_{S_e} \cdot L_e \cdot \frac{1}{E},$$

$$R_e^f = [R] \cdot V_{R_e} \cdot L_e,$$

$$S_c^f = [S] \cdot V_{S_c} \cdot L_c,$$

and

$$R_c^f = [R] \cdot V_{R_c} \cdot L_c.$$

Therefore, we can obtain E by using the following formula:

$$E = \frac{R_e^f \cdot S_c^f \cdot V_{S_e} \cdot V_{R_c}}{S_e^f \cdot R_c^f \cdot V_{R_e} \cdot V_{S_c}}, \tag{1}$$

where all terms on the right-hand side are precisely known.

Given the enrichment factor E, the amount of wild-type molecules scanned by the procedure, W, namely molecules either removed by RE-1 (which are therefore wild-type) or identi-

fied as wild-type at the sequence analysis step, is

$$W = S_e^f \cdot E. \tag{2}$$

Thus, under the following assumptions, we do not need to know $[S]$, $[R]$ or the amount of material lost during the runs of the two parallel protocols in order to know the RE-1 enrichment fold: *a*) the solutions can be kept sufficiently homogeneous for the purposes of drawing volumes of similar concentrations from them (we ensure this by a thorough mixing before drawing); *b*) volumes, at the range used, can be measured easily and accurately (as is the case); *c*) the normal loss of material during the run of the protocol (i.e., loss that is not due to RE-1 digestion) within any one treatment of a sample (RE-1–treated or untreated) does not substantially differ between sensitive and resistant molecules (as we confirm by observation; see Supplemental Section 7).

Finally, suppose that mutations of n different types have been identified in the ROI at the sequence analysis step (mutations that confer resistance to digestion by RE-1). Let M_i be the number of instances of mutation of type $i \in \{1, 2, ..., n\}$ identified at that step. The rate of mutation i is then

$$\frac{M_i}{W + \sum_{j=1}^{n} M_j}. \tag{3}$$

Since, in the denominator, $\sum_{j=1}^{n} M_j$ is negligible compared to W, it suffices to calculate the rate of mutation i as

$$\frac{M_i}{W}. \tag{4}$$

2.2 Practical considerations of amounts used for the treated and untreated samples

If sequencing were costless and unlimited in capacity, one could have started the MEMDS processing of both the RE-1–treated and RE-1–untreated samples with the same mix of genomic-DNA and mock-DNA sequences. Yet, while small amounts of mock-sequences could be easily identified in the RE-1–treated sample due to their enrichment, using the same amounts in the

RE-1–untreated sample where no enrichment for RE-1–resistant variants is carried out would require a large sequencing effort to trace them among the vast majority of wild-type sequences. On the other hand, using large amounts of mock-DNA would improve their sequence recovery in the RE-1–untreated sample but would consume HTS capacity at the expense of sperm sample sequences in the RE-1–treated sample. Likewise, while large amounts of genomic DNA can be used in the RE-1–treated sample due to the removal of many wild-type ROIs by RE-1 from the final sequencing input, using the same amount of genomic DNA in the RE-1–untreated sample would require a massive sequencing effort.

Therefore, to match the experimental design to the HTS coverage limitations, we carry out the following routine. From a single human-sperm DNA source we transfer a volume equivalent to ∼60–80 million haploid cells to the RE-1–treated tube, and a volume equivalent to exactly 5% of the initial amount taken for the RE-1–treated tube (i.e., ∼3–4 million haploid cells, respectively) to the RE-1–untreated tube. For each ROI to be analyzed, we use a mix of two linearized plasmids as the mock-DNA sample. These plasmids carry all of the ROI-flanking sequences that are necessary for processing by the MEMDS protocol, and each is designed to carry a unique stretch of mutations at the ROI that distinguishes it from the wild type, from natural mutants, and from the other plasmid (we use multiple mutations to make it practically impossible for the plasmid to be indistinguishable from natural mutants). Using the same plasmid-mix source tube, we add a volume equivalent to 7,500 copies from each linearized plasmid to the RE-1–treated tube (thus creating a genome:plasmid ratio close to 10,000:1) and 45,000 copies from each plasmid to the RE-1–untreated tube (creating a genome:plasmid ratio close to 100:1). Importantly, and as discussed above, the relative volumes drawn from any one source tube, not the absolute amounts of genomic and plasmid DNA, are the values that matter for the RE-1 enrichment fold calculation (Eq. 1).

3. *HBB* and *HBD* sequence features utilized by the MEMDS method

Different mutations in *HBB* that protect against malaria are known to have occurred and to have spread in human populations multiple times (Flint et al., 1998; Borg et al., 2009). HbS, the most notable mutation variant associated with resistance to malaria, involves a single base substitution (20A→T) in codon 6 of the *HBB* coding sequence that causes a Glutamate to Valine change (Allison, 1964; Hill et al., 1991; Serjeant and Serjeant, 2001). Some other point mutations and short deletions near the HbS mutation site are also known to confer resistance to malaria (Hardison et al., 2002; Hardison and Miller, 2002). The hemoglobin subunit delta (*HBD*) gene is expressed in adulthood together with *HBB* (Orkin, 1990). These two paralogs exhibit a high degree of homology, showing 80% identity in coding sequence and 93% identity in amino acid sequence. However, mutations in *HBD* are considered not to be protective against malaria, probably due to its low expression levels compared to *HBB*, which accounts for less than 3% of the hemoglobin in adults (Steinberg and Adams, 1991).

The *HBB* and *HBD* gene sequences that were selected for processing by the MEMDS method encompass 114 bases from exon 1, ranging from 32 nucleotides upstream of the mRNA translation start site to 81 nucleotides into the protein coding sequence (Fig. S3). This region is highly conserved between the two genes, which differ in only eight of the 114 bases. The region of interest (ROI) is a palindromic sequence found between positions 16-22 of the coding sequence, which forms the recognition site for the restriction enzyme Bsu36I (CCTNAGG) both in *HBB* and *HBD*. Since Bsu36I can tolerate any one of the four possible nucleotides at the central position of its recognition sequence, the ROI is limited to six of the seven nucleotides of this palindromic sequence. Therefore, Bsu36I serves as RE-1, which digests non-mutated (wild type) ROI sequences and enriches for *HBB*- and *HBD*-ROI mutation variants (Fig. S1, step 1).

The second restriction enzyme, HpyCH4III, which serves as RE-2 for the primary barcode attachment, digests the *HBB* and *HBD* gene segments at its recognition site (ACNGT), 45 bases

upstream of the 5' edge of the Bsu36I restriction site. The identity of the "N" base at the center of the HpyCH4III site is of central importance, as after digestion by HpyCH4III this base is found at the 3' terminus of the antisense strand that is extended to incorporate the primary barcode via a fill-in reaction (Fig. S1, step 2). Since *HBB* and *HBD* carry a different nucleotide at this "N" position of the HpyCH4III recognition site, the primary barcode oligo (oligo A) that initiates the fill-in reaction carried a randomized base at that position, matching either one of the two complementary bases, to allow for similar efficiencies of primary barcode synthesis for the two genes.

A region of 30 bases between the Bsu36I and the HpyCH4III sites is used as the annealing site for the primary barcode oligo, and a region of 28 bases starting 60 bases downstream of the 3' edge of the Bsu36I restriction site serves as the annealing site for the secondary barcode primer (oligo C, Fig. S1, step 5). This leaves a sequence of 15 bases upstream of the ROI site and 32 bases downstream of this site that are untouched by any primer and are amplified together with the enriched ROI elements. We use the differences between the *HBB* and *HBD* ROI 3'-flanking sequences to define HTS reads as belonging to either *HBB* or *HBD* during the sequence analysis step.

An advantage of the fact that, for each donor, the *HBB* and *HBD* ROIs are processed by MEMDS simultaneously and side by side in the same reaction by the same oligos, with the consequence that the genes are only separated by their unique and small sequence differences at the computational step, is that any mutational patterns arising in one gene and not in the other cannot be assigned to methodological artifacts. Such artifacts would have been expected to manifest themselves in both genes.

4. *In vitro* analysis of the effects of restriction-site mutations on Bsu36I activity

To study the *de novo* origination rate of the HbS mutation in *HBB* and of the parallel A$\rightarrow$T mutation in *HBD*, as well as the rates of other mutations in their vicinity, we applied the MEMDS method to the *HBB* and *HBD* genes from human-sperm DNA, exploiting the fact that codon 6 in both genes comprises a part of the recognition site for the Bsu36I restriction enzyme (RE-1) (Fig. S3). Therefore, because the enrichment of any mutation within the ROI site depends on the efficient blockage of Bsu36I digestion, we tested the ability of all single-base substitutions to effectively block Bsu36I digestion. For this purpose, we applied the deep mutational scanning approach (Melamed et al., 2013; Fowler and Fields, 2014) to generate a synthetic-DNA library of *HBB* segments carrying all possible single-base substitutions in the Bsu36I site and its flanking sequences. After incubating the library for 20 hours either with or without Bsu36I, high-throughput sequencing of the full-length products that were recovered from each sample allowed us to count each mutation variant and to calculate the fold difference in its frequency between the two samples, which serves as a proxy to the degree of resistance to Bsu36I cleavage. In accordance with the known consensus sequence for the Bsu36I site (CCTNAGG), we found that while the central base can tolerate any type of substitution, any single point mutation in the remaining 6 bases of the Bsu36I site is resistant to digestion (Fig. S4). The degree of resistance is similar to that of a variant that carries substitutions in all of the seven bases that constitute the Bsu36I site (the same set of mutations found in ALP13, one of the two artificial ROIs used to determine the Bsu36I-enrichment factor). Therefore, natural single-base substitutions in Bsu36I sites are effective substrates for enrichment by Bsu36I.

5. Generating *HBB* and *HBD* sequence datasets

We applied the MEMDS protocol to 7 sperm-DNA samples from donors of African ancestry (AFR1–7) and 4 samples from donors of European ancestry (EUR1–4) (see Table S1 for detail). As described in Supplemental Section 2, from each sample we aliquoted genomic DNA in an amount equivalent to 60–80 million sperm cells into one tube (referred to as “Bsu36I-treated”) and an amount equivalent to 5% of the cells (3–4 million sperm cells, respectively) into a second tube (referred to as “Bsu36I-untreated”). Each of the two reaction tubes was supplemented by a known amount of plasmid mixture that carries artificial Bsu36I-resistant *HBB* and *HBD* sequences. The Bsu36I-treated sample was treated with Bsu36I and HpyCH4III, and the Bsu36I-untreated sample was treated with HpyCH4III only. With the exception of the digestion step, the two samples were processed identically by the complete MEMDS procedure and sequenced.

Following standard quality filtration and merging of overlapping paired-end reads, reads were validated for carrying the 14-mer primary barcode and the 5-mer secondary barcode features, as well as the unique 5’ and 3’ sample-identifier sequences.

Control-guanine insertions designed to report primary barcode indirect labeling (see Fig S1 step 2, and Supplemental Section 14 for oligo A features) were found to be present in $\sim$1/9,000 reads for the Bsu36I-treated samples and $\sim$1/28,000 reads for the Bsu36I-untreated samples (Fig. S5A), implying an efficient 3’inverted-dT blockage of the primary barcode oligo. Yet, the observed difference in the fraction of reads with control-guanine insertions between the Bsu36I-treated and untreated samples suggests that the large amount of treated DNA in the former (leading, for example, to longer preparation times for some of the MEMDS step) and/or residual effects of Bsu36I digestion products may account for the elevated frequencies of indirect-labeling in the former.

After removing sequences with the control-guanine insertions, reads were sorted into sep-

arate *HBB* and *HBD* datasets based on their match to unique sequence features of each gene (see Methods and Fig. S3 for the exact sorting parameters). Consequently, each sperm sample produced four major datasets consisting of separate *HBB* and *HBD* sequencing pools for each of the Bsu36I-treated and untreated samples. Each read was then aligned against the donor's reference sequence and the presence of mutations and their types were noted per position. Next, reads were grouped into families based on their primary barcode sequences, where within each family, reads shared the same primary barcode and represented multiple copies of the same original target-DNA molecule, and each secondary barcode represented one of the $\leq$15 linearly amplified copies of that target molecule. Only families that passed the criteria discussed in the next section were selected for mutation-detection analysis.

6. Filtering families for mutation detection analysis

Three major parameters affect the level of accuracy by which a primary barcode family is considered as being originated from either a wild-type or a mutated target DNA molecule: *a*) the number of reads belonging to a primary barcode family (i.e. family size); *b*) the fraction of reads in the family having the same nucleotide (either a wild type or a mutation) in a given position (i.e., mutation frequency); and *c*) the number of secondary barcodes in a primary barcode family associated with either a wild-type base or a particular mutation (i.e., BC2 count).

For all donors and treatments, most primary barcode families contained multiple reads (Fig. S6). Yet, as previously reported for the MDS method (Jee et al., 2016), many families were represented by single reads. It is likely that many of these single-read families represent genuine labeling events that did not accumulate enough reads during the amplification steps, and are thus excluded from analysis and are a part of the general loss of material. Additionally, we found that between 20% and more than 50% of the single-read families had a primary barcode sequence that deviated by a Hamming distance of one from one of the primary barcode sequences of

a family with multiple reads (Fig. S6), suggesting that each of these single-read families is likely the result of a single-base error in the primary barcode of one of the reads in a multiple-reads family acquired during library preparation or HTS steps. Supporting this inference, a far smaller percentage of the primary barcodes of families with multiple reads were found to be at a Hamming distance of one away from other primary barcode families.

In addition to eliminating the barcode-error artifact, increasing the family size reduces the influence that sequence errors have on the final consensus sequence. Under the most stringent assumption that all the mutations appearing in sequences from the Bsu36I-untreated samples and in the ROI-flanking sequences from the Bsu36I-treated samples are due to HTS or PCR errors, gradually increasing the family-size cutoff reduced the acceptance rate of these false-positive mutations for both samples (Fig. S7A). We selected a minimum required family size of four reads for further mutation detection analysis, as increasing the family size cutoff beyond 4 reads did not noticeably improve mutation detection accuracy but continued to reduce the number of recovered families (Fig. S7A).

Increasing the mutation frequency cutoff, i.e., the minimal fraction of reads in a family carrying a particular nucleotide in a particular position allowing us to accept that nucleotide, reduced the fraction of false positive mutant families already when using low cutoff values, suggesting that the source of these mutations are late PCR errors or HTS errors that appear in small fractions within families (Fig. S7B). We selected a mutation-frequency cutoff of 0.7 (i.e., at least 70% of the family members carried either a wild-type base or a particular mutation at a given position), which provided a good balance between the number of mutations that were filtered out and the number of recovered families.

For each family, the number of unique secondary barcodes that were added after the linear amplification step and before the PCR amplification step corresponds to the number of unique linearly amplified copies of the original DNA molecule. Therefore, requiring multiple sec-

ondary barcodes allows us to reduce the error rate by ensuring that reads from distinct linear amplification events are used in the analysis. For the families with the highest read counts, we found that usually 4–5 of the unique secondary barcodes were more frequent than the remaining secondary barcodes, suggesting that while some of the linearly amplified copies of each ROI were PCR-amplified more efficiently than others, their repertoire was diverse enough and not over-dominated by a single linearly amplified copy (Fig. S8). We found negligible amounts of families with more than 15 unique secondary barcodes, which matches the maximal number of linearly amplified copies and supports the authenticity of these barcodes. Our control for secondary barcode relabeling suggests that such an event occurs once every 250-350 reads (Fig. S5B). Since both the originally labeled and the erroneously relabeled copies need to be sampled and included in the same family for their secondary barcodes to be miscounted twice, the negative effect of this event should be even smaller. Limiting mutation calling by requiring a minimum of two secondary barcodes associated with a particular nucleotide in a particular position as a condition for that nucleotide to be accepted (whether it is a wild type or a mutation), in addition to the family size cutoff, improved accuracy with a minimal effect on the number of recovered families (Fig. S7C). Thus, besides the major contribution of mutation enrichment by restriction-enzyme digestion to mutation detection accuracy, setting up a secondary barcode count cutoff as a regular part of the MEMDS procedure adds further precision in mutation calling in comparison to the MDS method (Jee et al., 2016).

Based on the above considerations we have selected the following combined threshold criteria: primary barcode families with at least four reads, a minimal within-family mutation-frequency cutoff of 70%, and the association of at least two secondary barcodes with each base. The flowchart of the algorithm we developed for base calling is provided in Fig. S9. Importantly, we use the same criteria for calling a wild-type family and a mutant family, thus eliminating any computational bias that would have been associated with different treatments

of wild type and mutant and could have affected the calculation of *de novo* mutation rates. In those rare events where neither the wild-type nucleotide nor a particular mutation in a $\geq$4-read family meet the mutation-frequency cutoff or the secondary barcode–count cutoff conditions at a certain position, the nucleotide identity at that position is declared ambiguous and the family is rejected from further analysis.

The numbers of families that were rejected or approved by these three cutoff criteria are shown for each library in Table S2. We also removed from further analysis *HBB* and *HBD* families that shared the same primary barcode sequences, which point to *HBB*/*HBD* chimeric artifacts that were generated during library amplification (we discuss these events more thoroughly in Supplemental Section 9). Datasheets S1–S44 describe the properties of each primary barcode family that passed the combined cutoff criteria.

7. MEMDS performance measures: Enrichment factors, numbers of genomes scanned, mutation recovery rate and error rate

In order to determine the origination rate of a particular mutation at a particular site, one must divide the number of sampled target sequences carrying that mutation by the total number of sampled target sequences. For the Bsu36I-untreated samples, the total number of target sequences sampled is derived solely from the number of families that are present in the sequencing output and that have passed the combined cutoff criteria. For the Bsu36I-treated samples, however, the total number of target sequences sampled (the number of genomes scanned by MEMDS) must include also the number of target sequences that have been eliminated due to Bsu36I digestion. We derive this number using the method described in Supplemental Section 2. To recapitulate, we divide the ratio between the number of artificial Bsu36I-resistant families and the number of wild-type families that result from applying the MEMDS procedure to the input mixture of the Bsu36I-treated sample by the analogous ratio from the untreated sample, while correcting

for the different volumes drawn for practical considerations from different source tubes, to obtain the Bsu36I-enrichment factor. The number of scanned wild-type target sequences (i.e., the number of target sequences that had been removed by Bsu36I digestion plus the number of target sequences that escaped Bsu36I digestion and formed wild-type families that passed the cutoff criteria) is then calculated by multiplying the number of wild-type families that passed the cutoff criteria by the Bsu36I-enrichment factor.

On average, about 13% of the input *HBB* and *HBD* wild-type ROIs were recovered in the Bsu36I-untreated samples (Fig. S10). A similar recovery rate of 15% was observed for the artificial ROIs in these samples, suggesting that both the genomic and the plasmid variants are processed similarly by this MEMDS workflow. Notably, the relatively low recovery rate of both target molecules is likely due to the overload input DNA, as no restriction enzyme-based depletion of wild-type ROI sequences takes place in these samples. However, these recovery rates satisfy the main purpose of the Bsu36I-untreated samples, which is to set the artificial ROI/genomic ROI ratio in the absence of Bsu36I enrichment (see Supplemental Section 2).

Following Bsu36I treatment, the wild-type ROI levels dropped to an average of $\sim$0.25% of their input levels, while the artificial ROI levels were at an average of $\sim$40% of their input levels, with *HBB* recovery being slightly higher than the recovery of *HBD* (Fig. S10). Given that point mutations at the ROI block Bsu36I digestion and are enriched similarly to the artificial sequences (Fig. S4), this percent recovery of the artificial ROIs suggests that at least a third of Bsu36I-resistant mutations that were present in the input sperm DNA were recovered in the Bsu36I-treated samples by the MEMDS procedure.

For each donor, the Bsu36I enrichment factors obtained from the artificial ROI/genomic ROI ratios showed a high degree of consistency between *HBB* and *HBD*, which reflects a similar activity of Bsu36I on both genes (Table S3). However, these enrichment factors displayed some variation across donors, ranging from a 64-fold enrichment to a 340-fold enrichment,

likely associated with either differences in Bsu36I activity due to batch effects or differences in the integrity of the sperm DNA (namely, the fraction of *HBB* and *HBD* ROI segments that were in a double-stranded state). In particular, the average enrichment factor of the four European samples (278.0 ± 47.8) was about 2.6-fold higher than the average enrichment factor of the seven African samples (107.3 ± 35.0). This difference in the average enrichment factor, which represents a Bsu36I digestion of 99.6% of the European *HBB* and *HBD* ROI sequences compared to 99.1% of the African ROI sequences, could arise due to the differences in semen composition between the two groups of donors that affect the double-strand state of the genomic DNA during its extraction. We found that the enrichments of the highly frequent G$\rightarrow$T and C$\rightarrow$T substitutions in the target DNA strand (C$\rightarrow$A and G$\rightarrow$A in the sequenced strand, respectively) in the ROIs exhibit differences between the eleven donors that followed the same direction as the differences between the Bsu36I-enrichment factors, which further supports the Bsu36I-enrichment-factor calculation by our approach (see Supplemental Section 8). Thus, given an average enrichment factor of $\sim$170-fold, the enrichment step of MEMDS alone boosts both the sequence coverage in the search for the target *de novo* mutations and the accuracy of mutation-detection by more than two orders of magnitude in comparison to the mutation rate in the Bsu36I-untreated samples.

Based on the calculated enrichment factors, the total number of wild-type *HBB* and *HBD* target sequences that were screened by Bsu36I (i.e., in the Bsu36I-treated samples) reaches about 300 million for each gene (Table S3). These numbers represent an average recovery rate of slightly more than 35% for wild-type target sequences, which is highly similar to the recovery rate of the artificial ROIs, further supporting the similar processing of both the plasmid and the genomic variants by the MEMDS procedure.

To calculate the MEMDS error rate, all G$\rightarrow$T, C$\rightarrow$T and C$\rightarrow$A mutations in the target-DNA strand have been excluded from analysis due to the reasons discussed in Supplemental

Section 8. Under the most stringent assumption that all remaining mutations found at the ROI and ROI-flanking sequences in the Bsu36I-untreated samples and at the ROI-flanking sequence in the Bsu36I-treated samples arose due to PCR or HTS errors, the total per base error rates for the Bsu36I-untreated and treated samples across all genes and donors in these unenriched sequences was 1.3×10^{-7} and 3.1×10^{-7} per base, respectively (Fig. S11A) (error rate calculation for an individual gene from a single donor would be less accurate due to low counts of non G→T, C→T and C→A mutations in these sequences). This 2.4-fold difference between the two error rates could result from a secondary influence of the large amount of treated DNA in the former, residual effects of Bsu36I-digestion products, or from moderate enrichment of mutations outside the Bsu36I-recognition site that may display weak inhibitory effects on Bsu36I. Taking a conservative approach, we selected the error rate at the ROI-flanking sequences in the Bsu36I-treated samples as our base line for the calculation of the MEMDS error rate. Therefore, to determine the MEMDS error rate, namely the error rate within the 6 bases of the ROI site in the Bsu36I-treated samples, for each donor and each of the two genes, we divided the error rate obtained for the ROI-flanking sequences of *HBB* and *HBD* in the Bsu36I-treated samples (2.9×10^{-7} and 3.3×10^{-7}, respectively) by their matching enrichment factors, reaching an average per base error rate of 2.3×10^{-9} ($\pm 1.2 \times 10^{-9}$) and 2.6×10^{-9} ($\pm 1.4 \times 10^{-9}$) for *HBB* and *HBD*, respectively, and an average of 2.5×10^{-9} ($\pm 1.3 \times 10^{-9}$) for both genes (Fig. S11B). Together with MEMDS's substantial reduction in sequencing cost, this error rate enables the identification of specific *de-novo* mutations at particular bases of interest that originate at rates even lower than the whole genome average mutation rate in humans.

8. G→T, C→T and C→A mutations

With respect to the ROI-flanking sequences, both the Bsu36I-treated and the Bsu36I-untreated samples displayed similar mutation patterns (Figs. S12 and S13), with Pearson's correlation

coefficients of 0.934 for *HBB* and 0.878 for *HBD*, for the mutations depicted in Fig. S13. Inspecting this mutational spectrum revealed high frequencies of single-base substitutions of three types, two of which were C→A and G→A, with average rates of $\sim 2.4 \times 10^{-6}$ ($\pm 1.4 \times 10^{-6}$) and $\sim 4.2 \times 10^{-6}$ ($\pm 2.2 \times 10^{-6}$), respectively, across both genes, treatments and donors. Since the consensus sequences are composed of reads of *HBB* and *HBD* at the sense orientation, these mutations are the reciprocals of the G→T and C→T mutations, respectively, that were present in the target, antisense DNA strand.

A major cause of G→T mutations is DNA damage occurring both endogenously under normal metabolic conditions (Tubbs and Nussenzweig, 2017; Ohno et al., 2014) and during DNA extraction and HTS preparation procedures (Arbeithuber et al., 2016; Costello et al., 2013; Bruskov et al., 2002). Reactive oxygen species (ROS) that arise as by-products of normal aerobic metabolism or due to the high temperatures used during DNA purification and PCR amplification steps can damage the genomic DNA by oxidizing guanine to 8-oxoguanine (8-oxoG), which in turn can pair up with adenine (8-oxoG:A) and promote a G:C→T:A mutation (Cheng et al., 1992). C→T mutations occur either naturally or *in vitro* by heat-induced hydrolytic deamination of either cytosine or 5-methylcytosine (5-meC) that generate uracil or thymine, respectively (Chen et al., 2014; Wang et al., 1982). These bases can then pair up with adenine and facilitate a C:G→T:A transition (Duncan and Miller, 1980).

In the *HBB* and *HBD* target antisense strands, G→T substitutions constituted $\sim$27% and $\sim$35% of the mutations found across Bsu36I-untreated and treated samples, respectively, and C→T substitutions were $\sim$67% and $\sim$56% of the mutations across the same samples, respectively. Compared to these high rates, we found the rates of the reciprocal substitutions in the same strands to be much lower: 4% and 6% for C→A, and less than 0.5% for G→A for each treatment (Fig. S12). As in previous studies that used one of the two DNA strands to explore *de novo* mutations, we take these imbalanced frequencies to indicate that the formation of 8-oxoG

and deaminated cytosines (or 5-meC) occurs in the DNA either *in vivo* or *in vitro* before the library amplification step, while the subsequent completion to full G:C→T:A and C:G→T:A substitutions, respectively, occurs during library amplification and not before then (Arbeithuber et al., 2016; Costello et al., 2013; Schmitt et al., 2012). Such DNA damages occurring *in vitro* before the library amplification step and/or representing the snapshot image of a disrupted ongoing process of base-damage and repair *in vivo* could result in mutational reads only when they occur in the target, antisense strand and not when they occur in the sequenced, sense strand, explaining the target-strand imbalance mentioned above.

Examining the mutation distribution along *HBB* and *HBD* sequences across all donors and treatments (Fig. S13) reveals that both the G→T and C→T substitutions in the target, antisense strand were enriched at the ROI site, suggesting that both these types of DNA damages were formed at the target strand either *in vivo* or *in vitro* prior to the enzymatic digestion step (Fig. S1, step 1) and conferred Bsu36I resistance. Indeed, 8-oxoG modifications placed at restriction sites or near them have been shown to interfere with the activity of multiple restriction enzymes (Allinson et al., 2001; Turk and Weitzman, 1995; Wood et al., 1990; Le Page et al., 2000; Hoppins et al., 2016). Similarly, generation of T:G or U:G mismatches that result from the deamination of cytosine or 5-meC have also been shown to inhibit enzymatic digestion (Lu et al., 1983; Glenn et al., 1994).

Importantly, while the frequency of the C→A mutation (the reciprocal of G→T) in the target strand was much lower than that of the G→T mutation, it was still noticeably higher than those of all other point mutations besides G→T and C→T, with an average rate of $\sim 3.9 \times 10^{-7}$ (Fig. S12). This observation implies that some of the guanine-oxidative damages could have affected the DNA sense strand (the non-target strand) early enough during library amplification and thus were able to produce mutations that were approved by the combined cutoff criteria.

The high frequency of the G→T and C→T substitutions in the target, antisense strand at

the ROI site in the Bsu36I-treated and untreated samples allowed us to calculate their enrichment fold in a manner entirely independent from the enrichment-fold calculation based on the artificial sequences described in Supplemental Section 2 (albeit more limited and less accurate than the latter, as these mutations were either too infrequent in or absent from the ROI of every Bsu36I-untreated sample). We found the enrichment of these substitutions to follow the same trend as the Bsu36I-enrichment factors, i.e., samples with higher enrichment factor values calculated from the artificial sequences showed increased G$\rightarrow$T and C$\rightarrow$T enrichments at the ROI site in comparison to samples with lower enrichment factor values (Fig. S14). In absolute terms, G$\rightarrow$T and C$\rightarrow$T enrichment values were lower than their matching enrichment factors calculated from the artificial sequences, likely due to 8-oxoG damages providing only incomplete resistance to Bsu36I digestion (Hoppins et al., 2016) and/or continuous DNA damage occurring after the restriction enzyme digestion and affecting uncut segments before the linear amplification step in both the Bsu36I-treated and untreated samples.

In addition to the enrichment of G$\rightarrow$T and C$\rightarrow$T mutations at the ROI site, we found also a G$\rightarrow$T enriched mutation at position 14 of *HBB* and *HBD*, two residues away from the Bsu36I site (Fig. S13). Indeed, 8-oxoG has been shown to affect neighboring bases and to compromise enzymatic digestion when placed near a restriction site (Koizume et al., 1998; Hoppins et al., 2016). Our finding that a complete G:C$\rightarrow$T:A mutation (i.e., the mutation is fixed in both strands) at position 14 has no effect on Bsu36I digestion (Fig. S4) further supports the effect of a single-stranded change such as 8-oxoG on Bsu36I digestion.

Given their high frequencies and unbalanced distribution between the two strands that disqualify G$\rightarrow$T, C$\rightarrow$T and C$\rightarrow$A substitutions in the target DNA strand as true, *de novo* mutations occurring in sperm cells, we excluded them (i.e., their sequencing output reciprocals C$\rightarrow$A, G$\rightarrow$A and G$\rightarrow$T) from the calculation of mutation rates. By comparison, Jee et al. (2016) removed the mutations C$\rightarrow$T, C$\rightarrow$A and A$\rightarrow$G from the MDS analysis of bacterial gene seg-

ments, suggesting that their high frequencies and strand occupancy bias reflect a snapshot image of base misincorporation and repair processes in the bacterial cells (Jee et al., 2016).

9. Repeated *de novo* mutations are not due to chimeric duplication events

As shown in Table 1 of the main text, in both the *HBB* and *HBD* ROIs, certain mutations are true, *de novo* mutations. Of the overall 49 single-base substitutions that were found across both genes and all donors not counting repetitions within donor, 14 occurred repeatedly in the same donor. Of the six deletion mutations that were found, the Hb-Leiden mutation (a deletion of either codon 6 or codon 7, which results in the same sequence) occurred repeatedly in *HBB* in seven of the 11 samples and in *HBD* in two of the 11 samples. Methodological artifacts cannot explain the correspondence that we see between *de novo* origination rates and observations of alleles in populations, as described in the main text and Supplemental Section 11. That being said, we additionally confirmed independently that these repetitions are not due to duplications of mutant families by artifactual chimeras that are generated during library preparation.

Chimeric sequences arising during PCR amplification are a common source of HTS artifacts, ranging from a few percent to nearly half of the sequences in individual libraries (Haas et al., 2011; Bradley and Hillis, 1997; Holcomb et al., 2014). A chimeric sequence can be generated during PCR due to low processivity of the DNA polymerase or insufficient elongation time that produce an incomplete DNA strand. Such a strand can anneal in one of the following cycles to a full-length strand of a second allele or a paralog gene and complete its extension, thus creating an Allele1/Allele2, or a Gene1/Gene2 chimeric product in addition to the PCR products of the two alleles, or genes, respectively. Therefore, a similar mechanism involving the interaction between an incomplete strand of a mutation variant and a full-length strand of a wild-type variant could theoretically result in the duplication of the mutation variant. Specifically, if a mutation-carrying strand ends prematurely and loses its primary barcode, when serving as a

primer in the following PCR cycle it will acquire a new barcode and could potentially lead to a second family that carries the mutation, where, in the unlikely event that such a family passes the combined filtration criteria, that second family is a false positive.

Since *HBB*/*HBD* chimeras in our experiment are identifiable, as they carry both *HBB*- and *HBD*-specific markers on different sides of the chimeric breakpoint (exemplified, for instance, by the relatively high frequency of *HBB* 9C→T or *HBD* 9T→C mutations), we used the *HBB*/*HBD* chimeras to estimate the probability that two separate families (each with its own primary barcode) that carry the same mutation actually arose from one family due to a chimeric event and thus represent a double-counting of the mutation. Specifically, since *HBB* and *HBD* share a high sequence identity, *HBB*/*HBD* chimeric artifacts could be generated as explained, by extending an incomplete strand of one paralog while using the full-length strand of the other paralog as a template during library preparation. Thus the extended strand acquires the primary barcode of the template strand. As *HBB* and *HBD* reads are sorted into distinct sequence analysis pools based on their unique sequence markers, both the chimeric family and the "template" family were identified by their shared primary barcode sequences and removed from further analysis (Supplemental Section 6). For AFR1, AFR2 and AFR3, who exhibited multiple instances of the HbS mutation, about 1% or less of the Bsu36I-treated families that passed our combined filtration criteria were identified as potential *HBB*/*HBD* chimeras (Table S2). Since each chimeric event between a mutant strand and a wild-type strand can result in either a wild type or a mutant duplication, the fraction of observed mutant families that constitutes artifactual duplicates of other mutant families during PCR is at most half the fraction of chimerism, namely $< 0.5\%$. This per mutant probability of artificial duplication is unable to account for the recurrence of mutations in our data, as not a single mutation is expected in the dataset to be a false positive due to double counting. In particular, it is unable to explain the repetition of HbS and Hb-Leiden in the data.

10. Genome-wide average point mutation, indel and A→T rates

The human genome-wide point mutation rate per base per generation is generally considered to be close to 1×10^{-8} (Shendure and Akey, 2015). Most recent estimates fall within the range of 1–1.5×10^{-8} (Kong et al., 2012; Campbell and Eichler, 2013; Francioli et al., 2015; Rahbari et al., 2016; Goldmann et al., 2016). Thus, we use the midpoint of this range, 1.25×10^{-8}, as a reference point for the sake of comparisons. Studies of the whole-exome mutation rate per base per generation average a bit higher, around 1.5×10^{-8} (Ségurel et al., 2014). However, while many of these studies are based on individuals with a given disease, whole-exome mutation rates from healthy individuals or neutral sites have reported rates closer to the 1.25×10^{-8} reference point (Ségurel et al., 2014; Campbell and Eichler, 2013). Either way, whether using 1.25 or the relatively high 1.5 as a reference point, no significance assignment reported in the main text is affected. Furthermore, since the human genome-wide per base per generation indel rate is more than $10\times$ smaller than the human genome-wide per base per generation point mutation rate (Kondrashov, 2003; Lynch, 2010; Turner et al., 2017), we use 1.25×10^{-9} as a slightly conservative reference point for the latter.

To obtain a per base point mutation rate across an ROI that can be compared to previous measures of the genome-wide average per base point mutation rate, we take into account the fact that 12 out of 18 of the possible point mutations across a single instance of the ROI are observable due to G→T, C→T and C→A exclusion. The effective average per base point mutation rate observed, μ^{ROI}, is then obtained as follows:

$$\mu^{\text{ROI}} = \frac{M}{\left(N \cdot \frac{12}{3}\right)}, \tag{5}$$

where M is the total number of point mutations observed across the ROI and N is the total number of families analyzed, namely the primary barcode families that have passed the combined cutoff criteria. In other words, the total number of point mutations observed is divided by the

maximal number of point mutations that could have been observed, where the latter is divided by 3 to obtain a per base rate that can be compared to previous measures of the genome-wide point mutation rate per base, since 3 mutations are possible per base. This simple calculation is suitable for the purpose of testing whether the average point mutation rates observed across the ROIs are higher than previously measured genome-wide point mutation rates, because here the ROI rate is inferred from 9 out of 12 possible point mutation types, where the average rate of the excluded mutation types is expected *a priori* to be no lower than the average rate of the included ones based on previous knowledge of mutation rates per type (Rahbari et al., 2016; Carlson et al., 2018).

The advantage of this method of comparing the ROI average to the genome-wide average per base point mutation rate is that it takes into account the particular sequence at the ROI. Alternatively, taking the ROI per base point mutation rate that is due to the 9 observable point mutation types only and comparing it to its genome-wide equivalent would require a complex adjustment of the genome-wide measure, whereas the goal here is merely to provide a general-sense comparison to a well known figure.

To obtain a per base indel rate across an ROI, we divide the total number of indel events (in our samples, only deletions have been observed) by the total number of bases examined across primary barcode families by MEMDS. Here, a complication arises from the fact that we can observe not only indels that are entirely contained within the ROI but also indels that partly overlap with the ROI, as those too are captured and enriched by MEMDS. A simple way of addressing this fact is to expand the number of base positions examined to include all positions between the farthest upstream and downstream breakpoints observed in the dataset, namely between position 14 and 24 (a stretch of 11 positions). The denominator of the indel rate calculation is then the total number of families observed multiplied by 11. For testing whether the observed indel rate is higher than expected from the genome-wide average, this

simple method is slightly conservative, because indels that overlap with the region between positions 14 and 24 but not with the ROI (e.g., a 12_14 deletion) are potentially possible but not observable and do not contribute toward the indel count.

To explain the calculation of the genome-wide average rates for the observable point-mutation types, take the A→T transversion for example. Based on a subset of *de novo* mutations with phasing information, the A:T→T:A transversion accounts for ∼6.5% of the total of point mutations across the human genome in males (Rahbari et al., 2016), while the A:T content across the human genome is ∼59% (Lander et al., 2001). Therefore, the average A→T mutation rate per adenine base per generation in males can be estimated as follows:

$$1.25 \cdot 10^{-8} \cdot \frac{6.5\%}{59\%} = 1.377 \cdot 10^{-9} < 1.4 \cdot 10^{-9} \tag{6}$$

Using a similar calculation with data on the relative frequencies of Extremely Rare Variants (ERVs) (Carlson et al., 2018) allows us to obtain the A→T mutation rates in the 3-mer, 5-mer and 7-mer contexts relevant to the *HBB* and *HBD* ROIs, namely the GAG, TGAGG and CTGAGGA contexts, respectively (using the supporting materials of Carlson et al., 2018), which are approximately 1.3, 1.2 and 0.9 $\times 10^{-9}$, respectively. In the same way, we obtain the genome-wide average rates, with or without the extended contexts, for the other observable point-mutations (Table S4). Thus, the local sequence up to the 7-mer context does not explain the high *de novo* origination rate of the A→T mutation in the *HBB* ROI in the African samples, consistent with the implication of complex genetic influences on the mutation-specific mutation origination rate (see main text). The same is true for the other point mutations that deviate from their expected genome-wide average rates (Table S4).

11. The correspondence between *de novo* rates and observations of alleles in populations

12 point mutation types in each ROI are observable by our method, all of which have been observed to occur *de novo* in at least one ethnicity and ROI. In addition, 23 deletions of up to size 3 are observable in each ROI (taking into account that size 2 and 3 observable deletions reach beyond the boundaries of the ROI, and that size 1 deletions at position 19 are not observable).

The expected rate of indels decreases with indel size (Lynch, 2010). Thus, because of the rarity of indels size >3, capping the analysis at this size provides for conservative P values, as including more indels would have only increased the fraction of indels that both have not been reported on HbVar and that are not observed *de novo*. (Indeed, the next deletion reported on HbVar that could have been observed by our method, 20_45del, is of size 26). Insertions are also relatively rare and neither were reported before in the ROIs in HbVar nor are observed here *de novo*, and thus their exclusion is also conservative for our statistical tests.

Of the point mutations, 8 have been reported on HbVar (16→CG, 16C→T, 17C→G, 17C→T, 20A→G, 20A→T, 20A→C, and 22G→C), and of the 23 observable indels of up to size 3, 5 have been reported on HbVar: 16delC, 17_18delCT, 18_19delTG, 19_21delGAG or the equivalent 22_24delGAG (the Hb-Leiden mutation), and 20delA, all in *HBB*. HbVar is an online database that gathers reports of all human hemoglobin variants from the literature and is arguably the largest source of information on this topic (Hardison et al., 2002; Hardison and Miller, 2002).

The *de novo* rates of deletion types reported on HbVar are significantly higher than the rates of deletion types not reported on HbVar, both combining the two ROIs ($P = 0.0033$, two-sided permutation test) and for the *HBB* ($P = 0.029$) and *HBD* ROIs separately ($P = 0.0056$). However, because the Hb-Leiden mutation has an exceptionally high *de novo* rate among the deletions, in order to find out whether this mirroring between deletion *de novo* rates and reports

of these mutations on HbVar is driven by the Hb-Leiden mutation alone or extends also to other mutations, we compare the fraction of deletion types reported on HbVar that have been observed *de novo* at least once in our data to the same fraction among deletion types not reported on HbVar. The former fraction is significantly larger than the latter both when combining the two ROIs ($P = 0.0078$, two-sided Fisher's exact test) and for each ROI separately (***HBB***: $P = 0.048$, ***HBD***: $P = 0.0056$, two-sided Fisher's exact test). While these tests already show that the results are not driven only by the high Hb-Leiden *de novo* rate, when combining the two ROIs due to the similarity of indel types observed between them, the results remain significant also when excluding the Hb-Leiden mutation completely ($P = 0.026$ and $P = 0.024$, two-sided Fisher's exact test), further demonstrating that the mirroring between these mutation's *de novo* rates and observations of them in populations extends beyond the Hb-Leiden mutation. Indeed, note that of the 6 indel types observed by us *de novo* in any ROI and ethnicity, 4 are included in the 5 deletions reported on HbVar (16delC, 17_18delCT, 19_21delGAG or 22_24delGAG, and 20delA), and of the deletions reported on HbVar, only one has not been observed *de novo* (18_19delTG). In accord with this observation, most of the deletion mutations we observe can already be seen to be taking part in the mirroring effect mentioned above.

For the point mutations, a much smaller list of observable types is available, all of which have been observed *de novo* at least once. In addition, 3 out of the 4 point mutations not reported on HbVar are synonymous (18T→G, 18T→A and 18T→C), for which reason one could not have expected them to be reported on HbVar to begin with. Therefore, it is not possible to apply the mirroring analysis above to the point mutations.

Examining instead the frequencies of alleles in the ROIs reported in population genetic data from gnomAD exome sequencing (Karczewski et al., 2020) provided by Ensembl (release 102) (Yates et al., 2020), where only sparse data is available, we find that only 3 point mutations in total were reported at non-zero frequencies, all in *HBB*. These are the HbS mutation, 16C→G

and 16C→T. Two are of high and one is of medium *de novo* rate in our data. We also find that the Hb-Leiden mutation, notably the most frequent *de novo* mutation also in the *HBD* ROI, is the only variant of non-zero frequency reported on gnomAD in the *HBD* ROI.

We conclude that the correspondence between *de novo* rates and observations of alleles in populations applies to both types of mutation and extends beyond the HbS and Hb-Leiden mutations. Further adding to this analysis, while HbS is common mostly in Africa and in some populations in the Asian malaria belt (Flint et al., 1998), it appears *de novo* in our African but not in our European donors, and while the Hb-Leiden mutation has been reported across the globe (Hardison et al., 2002; Hardison and Miller, 2002), it appears *de novo* in both our African and European donors.

The correspondence above-mentioned could not have been predicted from the genome-wide average (GWA) rates of the mutations involved. In particular, different indel GWA rates are not assigned to different indel types as defined here but to different indel sizes (the smaller the more frequent; Lynch, 2010), a minor effect which only further contrasts with the fact that the Hb-Leiden deletion (our largest) has the highest *de novo* rate. In addition, the HbS mutation is an A→T transversion, the least frequent point mutation type on average.

12. The possibility of gene conversion explaining the high 20A→T rate in the African *HBB* gene

The only paralog or pseudogene with sufficient homology (Borg et al., 2009) to *HBB* as to allow for gene conversion and thus a single-base mutation in *HBB* without leaving other sequence differences that would have been detected is *HBD*. Yet, neither our African nor our European donors are carriers of the 20A→T *HBD* mutation. Thus, this mutation could not have been transferred from *HBD* to *HBB* via gene conversion. We further verified by a BLAST search for the focal sequence that no substantial homology exists to other parts of the human genome. We

cannot exclude the possibility of a transfer of this mutation via gene conversion based on very short sequences originating from elsewhere in the genome.

13. Supporting materials and methods: Bsu36I single-base substitution sensitivity assay

Nineteen oligos (BSU 1-19) carrying the first 37 bases of *HBB*, each with a randomized nucleotide at a single position within the seven bases of the Bsu36I recognition site or at one of the six bases that flank this site from either side were mixed with a similar oligo with all of the seven bases of the Bsu36I recognition site replaced by the sequence TTATGTT (Bsu36I^{R}). This oligos mixture was PCR-amplified for 25 cycles using Q5 DNA polymerase and primers that match the Illumina adapter sequences flanking the *HBB* region in each oligo (Primers BSU F1 and BSU R1). 150 ng of this PCR product were subjected to 20 hours incubation at 37°C with or without 5 units Bsu36I. Digestion products were purified, re-amplified (Primers BSU F2 and BSU R2) and paired-end sequenced by Illumina MiSeq. To calculate Bsu36I sensitivity, mutation variant reads were counted and the frequency of each variant in the Bsu36I-treated sample was divided by its frequency in the Bsu36I-untreated sample. All ratios were normalized to the ratio of the Bsu36I^{R} variant that was considered to be 100% resistant to Bsu36I.

14. DNA oligos

14.1. Oligos for Sperm DNA library preparation

NAME	Oligo A
DESCRIPTION	Direct attachment of primary barcode
SEQUENCE	P-*CTCTTTCCCTACACGACGCTCTTCCGATCT*(14N)XXXX WGTGTTCACTAGCAAgCCTCAAACAGACACC-InvdT
SEQUENCE FEATURES	P – 5' phosphate, to improve subsequent degradation by 5' exonucleases. In italics – part of Illumina TruSeq Universal Adapter P5 that carries the sequence for read-1 sequencing primer. (14N) – Primary Barcode, unique to each labeled molecule. XXXX – Donor identifier (ID)-1, a sequence of four bases unique to each donor. Underlined sequence – complementary to *HBB* and *HBD* antisense strands; covers 30 bases between HpyCH4III digestion site and the -1 position relative to the mRNA translation start site; W (either A or T) was designed to equally prefer the single base difference between the 3' terminus of *HBB* (3'T) and *HBD* (3'A) antisense strands produced by HpyCH4III digestion. Lowercased "g" –a base insertion designed to identify events of erroneous extension and amplification promoted by unblocked (3' InvdT missing) Oligo A, if any. InvdT – 3' inverted dT modification, designed to block extension by Q5 DNA polymerase.

NAME	Oligo B
DESCRIPTION	Linear amplification of barcoded strand
SEQUENCE	*ApsApsTpsGpsApsTACGGCGACCACCGAGATCTACA*CTCT TTCCCTACACGACGCTC
SEQUENCE FEATURES	Underlined sequence – region complementary to the fill-in product (barcode-labeled strand) by Oligo A. In italics – completes the 5' part of Illumina TruSeq Universal Adapter P5. *ps* – phosphorothioate; protect linearly amplified sequences from 5' exonuclease degradation.

NAME	Oligo C
DESCRIPTION	Secondary barcode
SEQUENCE	*GTGACTGGAGTTCAGACGTGTGCTCTTCCGATCT*(5N)XXXXATCCACGTTCACCTTGCaCCACAGGGCAGTAAC
SEQUENCE FEATURES	In italics – Part of Illumina TruSeq Universal Adapter P7 that carries the sequence for read-2 and the index sequencing primers. (5N) – Secondary Barcode, unique to each linearly amplified sequence. XXXX – Donor identifier (ID)-2, four bases unique to each donor; Underlined sequence – region complementary to *HBB* and *HBD* sequence. Lowercase "a" – perfect match for *HBB*, a mismatch for *HBD*.

NAME	Oligo D
DESCRIPTION	Relabeling-control primer
SEQUENCE	GTGACTGGAGTTCAGACGTGTGCTCTTCCGATCTAGTGTAAAAATCCACGTTCACCTTGCaCCACAGGGCAGTAAC
SEQUENCE FEATURES	Identical to Oligo C, but with the sequence AGTGT replacing the Secondary barcode sequence (5N), and AAAA replacing ID-2 sequence XXXX (underlined)

NAME	Oligo E
DESCRIPTION	Forward amplification primer for first and second PCR reactions
SEQUENCE	AATGATACGGCGACCACCGAGATCTAC
SEQUENCE FEATURES	Matches the 5' edge generated by Oligo B

NAME	Oligo F1
DESCRIPTION	Reverse amplification primer for first PCR reaction
SEQUENCE	GTGACTGGAGTTCAGACGTGTGCTC
SEQUENCE FEATURES	Matches the 3' edge generated by Oligo C

NAME	Oligo F2
DESCRIPTION	Reverse amplification primer for second PCR reaction
SEQUENCE	CAAGCAGAAGACGGCATACGAGATXXXXXXGTGACTGGAGTTCAGACGTGTGC
SEQUENCE FEATURES	Completes the Illumina TruSeq Universal Adapter P7; XXXXXX stands for the Illumina index sequence.

14.2. Oligos for Bsu36I-mutation sensitivity test

NAME	BSU 1-19
DESCRIPTION	Template oligos for single-base mutation library generation by PCR
SEQUENCE	*TACACGACGCTCTTCCGATCT***ATGGTGCAYCTGACTcctgaggAGAAGTCTGCCGTTA***AGATCGGAAGAGCACACGTCTG*
SEQUENCE FEATURES	In Italics – part of Illumina TruSeq Universal Adapters P5 and P7. In bold – the first 37 bases of *HBB* WT sequence beginning from the mRNA translation start site (ATG). In lowercased letters – Bsu36I recognition site. Underlined sequence – 19 bases in *HBB* sequence that were subjected to randomized mutagenesis. Each oligo from BSU1 to BSU19 carries a randomized base mixture at the equivalent position (N, N, N, B, D, V, D, D, V, H, B, H, H, B, H, B, N, N, N, respectively).

NAME	BSU 20 (Bsu36I^R)
DESCRIPTION	Bsu36I resistance variant
SEQUENCE	*TACACGACGCTCTTCCGATCT***ATGGTGCAYCTGACTttatgttAGAAGTCTGCCGTTA***AGATCGGAAGAGCACACGTCTG*
SEQUENCE FEATURES	Identical to the Oligo described for BSU 1-19, but with the Bsu36I recognition sequence replaced from cctgagg to ttatgtt.

NAME	BSU F1
DESCRIPTION	Forward primer for generating double-stranded BSU library (dsBSU) by PCR together with BSU R1 primer and BSU1-20 oligos' mixture as a template
SEQUENCE	*ACACTCTTTCCCTACACGACGCTCTTCCGATCT***ATGGTGCA**
SEQUENCE FEATURES	In Italics – part of Illumina TruSeq Universal Adapters P5. In bold – 5' of *HBB* segment. Undelined – sequence matching BSU 1-20 oligos.

NAME	BSU R1
DESCRIPTION	Reverse primer for generating double-stranded BSU library (dsBSU) by PCR together with BSU F1 primer and BSU1-20 oligos' mixture as a template
SEQUENCE	*GTGACTGGAGTTCAGACGTGTGCTCTTCCGATCT***TAACGGCA**
SEQUENCE FEATURES	In Italics – part of Illumina TruSeq Universal Adapters P7. In bold – 3' *HBB* segment. Undelined – sequence matching to BSU 1-20 oligos.

NAME	BSU F2
DESCRIPTION	Forward primer for generating the BSU library by PCR together with BSU R2 primer, and Bsu36I treated and Non-treated dsBSU as templates, for high-throughput sequencing.
SEQUENCE	AATGATACGGCGACCACCGAGATCTACACTCTTTCCCTACACGACGCTCTTCCGATCTATGGTGCA
SEQUENCE FEATURES	Completes the Illumina TruSeq Universal Adapters P5. Underlined - sequence matching to dsBSU library.

NAME	BSU R2
DESCRIPTION	Reverse primer for generating the BSU library by PCR together with BSU F2 primer, and Bsu36I treated and Non-treated dsBSU as templates, for high-throughput sequencing.
SEQUENCE	CAAGCAGAAGACGGCATACGAGATXXXXXXGTGACTGGAGTTCAGACGTGTGC
SEQUENCE FEATURES	Completes the Illumina TruSeq Universal Adapter P7. XXXXXX stands for the Illumina index sequence. Underlined - sequence matching to dsBSU library.

15. Supplemental datasheets

The below supplemental datasheet files accompany this manuscript:

SD1_AFR1_HBB_Bsu36I-treated.txt _
SD2_AFR1_HBB_Bsu36I-untreated.txt
SD3_AFR1_HBD_Bsu36I-treated.txt
SD4_AFR1_HBD_Bsu36I-untreated.txt
SD5_AFR2_HBB_Bsu36I-treated.txt
SD6_AFR2_HBB_Bsu36I-untreated.txt
SD7_AFR2_HBD_Bsu36I-treated.txt
SD8_AFR2_HBD_Bsu36I-untreated.txt
SD9_AFR3_HBB_Bsu36I-treated.txt
SD10_AFR3_HBB_Bsu36I-untreated.txt
SD11_AFR3_HBD_Bsu36I-treated.txt
SD12_AFR3_HBD_Bsu36I-untreated.txt
SD13_AFR4_HBB_Bsu36I-treated.txt
SD14_AFR4_HBB_Bsu36I-untreated.txt
SD15_AFR4_HBD_Bsu36I-treated.txt
SD16_AFR4_HBD_Bsu36I-untreated.txt
SD17_AFR5_HBB_Bsu36I-treated.txt
SD18_AFR5_HBB_Bsu36I-untreated.txt
SD19_AFR5_HBD_Bsu36I-treated.txt
SD20_AFR5_HBD_Bsu36I-untreated.txt
SD21_AFR6_HBB_Bsu36I-treated.txt
SD22_AFR6_HBB_Bsu36I-untreated.txt
SD23_AFR6_HBD_Bsu36I-treated.txt
SD24_AFR6_HBD_Bsu36I-untreated.txt
SD25_AFR7_HBB_Bsu36I-treated.txt
SD26_AFR7_HBB_Bsu36I-untreated.txt
SD27_AFR7_HBD_Bsu36I-treated.txt
SD28_AFR7_HBD_Bsu36I-untreated.txt
SD29_EUR1_HBB_Bsu36I-treated.txt
SD30_EUR1_HBB_Bsu36I-untreated.txt
SD31_EUR1_HBD_Bsu36I-treated.txt
SD32_EUR1_HBD_Bsu36I-untreated.txt
SD33_EUR2_HBB_Bsu36I-treated.txt
SD34_EUR2_HBB_Bsu36I-untreated.txt
SD35_EUR2_HBD_Bsu36I-treated.txt
SD36_EUR2_HBD_Bsu36I-untreated.txt
SD37_EUR3_HBB_Bsu36I-treated.txt
SD38_EUR3_HBB_Bsu36I-untreated.txt
SD39_EUR3_HBD_Bsu36I-treated.txt
SD40_EUR3_HBD_Bsu36I-untreated.txt
SD41_EUR4_HBB_Bsu36I-treated.txt
SD42_EUR4_HBB_Bsu36I-untreated.txt
SD43_EUR4_HBD_Bsu36I-treated.txt
SD44_EUR4_HBD_Bsu36I-untreated.txt

Description:

These files describe features of primary barcode families that were approved for analysis according to the following cutoff criteria:

A) Family size ≥ 4

B) Mutation frequency ≥ 0.7

C) Secondary barcode ≥ 2

For each primary barcode family, the data below are shown:

1. Primary_barcode: 14 bases of a randomized barcode sequence that tag a single gene (*HBB* or *HBD*) from a single sperm cell.

2. Read_consensus: The consensus sequence obtained after using the combined cutoff criteria shown above. "WT" stands for a wild-type sequence in the ROI and the ROI flanking sequences (i.e., no mutation passed the mutation-frequency and secondary barcode cutoff criteria in these primary barcode families). When a mutation is specified, its identity is described by its position relative to the first sequenced base in the read after the sample identifier-1 (ID-1) sequence, followed by the identity of the original base at that position and the identity of the substituting base (for example, 50AT stands for an A$\rightarrow$T substitution at position 50). Mutation identifiers with a hyphen located at the position of the original base or the substituting base describe insertion or deletion mutations respectively. When multiple mutations in a single primary barcode family are approved by the combined cutoff criteria, the mutation identifiers are separated by semicolons.

3. HBB_consensus (or HBD_consensus): The same as for the Read_consensus, but with the mutation position in the mutation identifier adjusted to the mRNA-translation start site.

4. Mutation_frequency: The fraction of reads that carry the consensus mutation in the primary barcode family. Due to the applied mutation-frequency cutoff, only mutations (or WT bases) with a frequency of at least 0.7 are shown. When multiple mutations in a single primary barcode family are approved by the combined cutoff criteria, the mutation frequencies are separated by semicolons. For the WT consensus sequences, a mutation-frequency value represents the fraction of reads in the primary barcode family with no mutations. PCR or sequencing errors may reduce the fraction of complete WT sequences in a primary barcode family, sometimes below the mutation frequency cutoff of 0.7. Yet, since the mutation-frequency cutoff is applied for a single position at a time, the frequency of the WT base in each of these mutated positions exceeded the mutation-frequency cutoff.

5. Mutation_count: The number of reads that carry the consensus mutation in a primary barcode family (the numerator for the mutation frequency calculation). When multiple mutations in a single primary barcode family are approved by the combined cutoff criteria, the mutation counts are separated by semicolons. For the WT consensus sequences, a mutation-count value represents the number of reads in the primary barcode family with no mutations.

6. Total_count: The number of reads in a primary barcode family (the denominator for the mutation frequency calculation). Due to the applied family-size cutoff, only families with more than 4 reads are shown.

7. Number of mutations: The number of mutations in a primary barcode family that were approved by the combined cutoff criteria.

8. Unique_secondary_barcodes: The number of unique secondary barcodes that are associated with the consensus mutation in a primary barcode family. Due to the applied secondary barcode number cutoff, only mutations (or WT bases) with 2 or more secondary barcodes are shown. When multiple mutations in a single primary barcode family are approved by the

combined cutoff criteria, the mutation frequencies are separated by semicolons. For the WT consensus sequences, if a mutation in the primary barcode family was disqualified by the combined cutoff criteria (i.e., PCR or sequencing errors), the number of unique secondary barcodes associated with the WT base at that position is shown (with multiple positions with disqualified mutations producing multiple unique secondary barcode values for the WT consensus sequence, separated by semicolons).

9. The *HBB* consensus sequences 9CT;16C-;17C-;19GT;21GT;22-TT and 9CT;16CA;18T-;21-A;22GC belong to the spike-in plasmids ALP13 and ALP17 carrying the artificial ROI sequences TTATGTT and ACGAGAC, respectively, instead of the Bsu36I site CCTGAGG. The *HBD* consensus sequences 16C-;17C;19GT;21GT;22-TT and 16CA;18T-;21-A;22GC belong to the spike-in plasmids ALP16 and ALP18 that carries the same artificial ROI sequences. The consensus sequence 39CT;45-ATAA;47CA;49G-;50A-;51G-;52G-, appearing twice in the AFR1_HBB_Bsu36I-treated, once in EUR2_HBB_Bsu36I_treated and once in AFR7_HBB_Bsu36I_treated datasets is an ALP21 plasmid (ATAACAT instead of the Bsu36I site) contaminant that was not used in this study.

References

Abascal F, Harvey LM, Mitchell E, Lawson AR, Lensing SV, Ellis P, Russell AJ, Alcantara RE, Baez-Ortega A, Wang Y, et al.. 2021. Somatic mutation landscapes at single-molecule resolution. *Nature* **593**: 405–410.

Allinson SL, Dianova II, and Dianov GL. 2001. DNA polymerase β is the major dRP lyase involved in repair of oxidative base lesions in DNA by mammalian cell extracts. *EMBO J* **20**: 6919–6926.

Allison AC. 1964. Polymorphisms and natural selection in human populations. *Cold Spring Harb Symp Quant Biol* **29**: 137–149.

Arbeithuber B, Makova KD, and Tiemann-Boege I. 2016. Artifactual mutations resulting from DNA lesions limit detection levels in ultrasensitive sequencing applications. *DNA Res* **23**: 547–559.

Borg J, Georgitsi M, Aleporou-Marinou V, Kollia P, and Patrinos GP. 2009. Genetic recombination as a major cause of mutagenesis in the human globin gene clusters. *Clin Biochem* **42**: 1839–1850.

Bradley RD and Hillis DM. 1997. Recombinant DNA sequences generated by PCR amplification. *Mol Biol Evol* **14**: 592–593.

Bruskov VI, Malakhova LV, Masalimov ZK, and Chernikov AV. 2002. Heat-induced formation of reactive oxygen species and 8-oxoguanine, a biomarker of damage to DNA. *Nucleic Acids Res* **30**: 1354–1363.

Campbell CD and Eichler EE. 2013. Properties and rates of germline mutations in humans. *Trends Genet* **29**: 575–584.

Carlson J, Locke AE, Flickinger M, Zawistowski M, Levy S, Myers RM, Boehnke M, Kang HM, Scott LJ, Li JZ, et al.. 2018. Extremely rare variants reveal patterns of germline mutation rate heterogeneity in humans. *Nat Commun* **9**: 3753.

Casbon JA, Osborne RJ, Brenner S, and Lichtenstein CP. 2011. A method for counting PCR template molecules with application to next-generation sequencing. *Nucleic Acids Res* **39**: e81–e81.

Chen G, Mosier S, Gocke CD, Lin MT, and Eshleman JR. 2014. Cytosine deamination is a major cause of baseline noise in next-generation sequencing. *Mol Diagn Ther* **18**: 587–593.

Cheng KC, Cahill DS, Kasai H, Nishimura S, and Loeb LA. 1992. 8-hydroxyguanine, an abundant form of oxidative DNA damage, causes G>T and A>C substitutions. *J Biol Chem* **267**: 166–172.

Cibulskis K, Lawrence MS, Carter SL, Sivachenko A, Jaffe D, Sougnez C, Gabriel S, Meyerson M, Lander ES, and Getz G. 2013. Sensitive detection of somatic point mutations in impure and heterogeneous cancer samples. *Nat Biotechnol* **31**: 213.

Costello M, Pugh TJ, Fennell TJ, Stewart C, Lichtenstein L, Meldrim JC, Fostel JL, Friedrich DC, Perrin D, Dionne D, et al.. 2013. Discovery and characterization of artifactual mutations in deep coverage targeted capture sequencing data due to oxidative DNA damage during sample preparation. *Nucleic Acids Res* **41**: e67–e67.

Duncan BK and Miller JH. 1980. Mutagenic deamination of cytosine residues in DNA. *Nature* **287**: 560–561.

Flint J, Harding RM, Boyce AJ, and Clegg JB. 1998. The population genetics of the haemoglobinopathies. *Baillière's Clin Haem* **11**: 1–51.

Fowler DM and Fields S. 2014. Deep mutational scanning: a new style of protein science. *Nat Methods* **11**: 801.

Fox EJ, Reid-Bayliss KS, Emond MJ, and Loeb LA. 2014. Accuracy of Next Generation Sequencing platforms. *Next Gener Seq Appl* **1**.

Francioli LC, Polak PP, Koren A, Menelaou A, Chun S, Renkens I, Van Duijn CM, Swertz M,

Wijmenga C, Van Ommen G, et al.. 2015. Genome-wide patterns and properties of *de novo* mutations in humans. *Nat Genet* **47**: 822.

Glenn T, Waller D, and Braun MJ. 1994. Increasing proportions of uracil in DNA substrates increases inhibition of restriction enzyme digests. *BioTechniques* **17**: 1086–1090.

Goldmann JM, Wong WS, Pinelli M, Farrah T, Bodian D, Stittrich AB, Glusman G, Vissers LE, Hoischen A, Roach JC, et al.. 2016. Parent-of-origin-specific signatures of *de novo* mutations. *Nat Genet* **48**: 935.

Gregory MT, Bertout JA, Ericson NG, Taylor SD, Mukherjee R, Robins HS, Drescher CW, and Bielas JH. 2016. Targeted single molecule mutation detection with massively parallel sequencing. *Nucleic Acids Res* **44**: e22–e22.

Haas BJ, Gevers D, Earl AM, Feldgarden M, Ward DV, Giannoukos G, Ciulla D, Tabbaa D, Highlander SK, Sodergren E, et al.. 2011. Chimeric 16S rRNA sequence formation and detection in Sanger and 454-pyrosequenced PCR amplicons. *Genome Res* **21**: 494–504.

Hardison R and Miller W. 2002. "Globin Gene Server," http://globin.cse.psu.edu/ Accessed 10/5/2019.

Hardison RC, Chui DH, Giardine B, Riemer C, Patrinos GP, Anagnou N, Miller W, and Wajcman H. 2002. HbVar: a relational database of human hemoglobin variants and thalassemia mutations at the globin gene server. *Hum Mutat* **19**: 225–233.

Hestand MS, Van Houdt J, Cristofoli F, and Vermeesch JR. 2016. Polymerase specific error rates and profiles identified by single molecule sequencing. *Mutat Res* **784**: 39–45.

Hiatt JB, Pritchard CC, Salipante SJ, O'Roak BJ, and Shendure J. 2013. Single molecule

molecular inversion probes for targeted, high-accuracy detection of low-frequency variation. *Genome Res* **23**: 843–854.

Hill AV, Allsopp CE, Kwiatkowski D, Anstey NM, Twumasi P, Rowe PA, Bennett S, Brewster D, McMichael AJ, and Greenwood BM. 1991. Common West African HLA antigens are associated with protection from severe malaria. *Nature* **352**: 595–600.

Hoang ML, Kinde I, Tomasetti C, McMahon KW, Rosenquist TA, Grollman AP, Kinzler KW, Vogelstein B, and Papadopoulos N. 2016. Genome-wide quantification of rare somatic mutations in normal human tissues using massively parallel sequencing. *P Natl Acad Sci USA* **113**: 9846–9851.

Holcomb C, Rastrou M, Williams T, Goodridge D, Lazaro A, Tilanus M, and Erlich H. 2014. Next-generation sequencing can reveal in vitro-generated PCR crossover products: some artifactual sequences correspond to HLA alleles in the IMGT/HLA database. *Tissue Antigens* **83**: 32–40.

Hong J and Gresham D. 2017. Incorporation of unique molecular identifiers in truseq adapters improves the accuracy of quantitative sequencing. *BioTechniques* **63**: 221–226.

Hoppins JJ, Gruber DR, Miears HL, Kiryutin AS, Kasymov RD, Petrova DV, Endutkin AV, Popov AV, Yurkovskaya AV, Fedechkin SO, et al.. 2016. 8-Oxoguanine affects DNA backbone conformation in the EcoRI recognition site and inhibits its cleavage by the enzyme. *PloS One* **11**.

Jee J, Rasouly A, Shamovsky I, Akivis Y, Steinman SR, Mishra B, and Nudler E. 2016. Rates and mechanisms of bacterial mutagenesis from maximum-depth sequencing. *Nature* **534**: 693.

Jinek M, Chylinski K, Fonfara I, Hauer M, Doudna JA, and Charpentier E. 2012. A programmable dual-RNA–guided DNA endonuclease in adaptive bacterial immunity. *Science* **337**: 816–821.

Karczewski K, Francioli L, MacArthur D, et al.. 2020. The mutational constraint spectrum quantified from variation in 141,456 humans. *Nature* **581**: 434–443.

Kennedy SR, Schmitt MW, Fox EJ, Kohrn BF, Salk JJ, Ahn EH, Prindle MJ, Kuong KJ, Shen JC, Risques RA, et al.. 2014. Detecting ultralow-frequency mutations by Duplex Sequencing. *Nat Protoc* **9**: 2586.

Kinde I, Wu J, Papadopoulos N, Kinzler KW, and Vogelstein B. 2011. Detection and quantification of rare mutations with massively parallel sequencing. *P Natl Acad Sci USA* **108**: 9530–9535.

Koizume S, Inoue H, Kamiya H, and Ohtsuka E. 1998. Neighboring base damage induced by permanganate oxidation of 8-oxoguanine in DNA. *Nucleic Acids Res* **26**: 3599–3607.

Kondrashov AS. 2003. Direct estimates of human per nucleotide mutation rates at 20 loci causing Mendelian diseases. *Hum Mutat* **21**: 12–27.

Kong A, Frigge ML, Masson G, Besenbacher S, Sulem P, Magnusson G, Gudjonsson SA, Sigurdsson A, Jonasdottir A, Jonasdottir A, et al.. 2012. Rate of *de novo* mutations and the importance of father's age to disease risk. *Nature* **488**: 471.

Lander E, Linton L, Birren B, et al.. 2001. Initial sequencing and analysis of the human genome. *Nature* **409**: 860–921.

Le Page F, Randrianarison V, Marot D, Cabannes J, Perricaudet M, Feunteun J, and Sarasin A.

2000. BRCA1 and BRCA2 are necessary for the transcription-coupled repair of the oxidative 8-oxoguanine lesion in human cells. *Cancer Res* **60**: 5548–5552.

Lee DF, Lu J, Chang S, Loparo JJ, and Xie XS. 2016. Mapping DNA polymerase errors by single-molecule sequencing. *Nucleic Acids Res* **44**: e118–e118.

Lou DI, Hussmann JA, McBee RM, Acevedo A, Andino R, Press WH, and Sawyer SL. 2013. High-throughput DNA sequencing errors are reduced by orders of magnitude using circle sequencing. *P Natl Acad Sci USA* **110**: 19872–19877.

Lu AL, Clark S, and Modrich P. 1983. Methyl-directed repair of DNA base-pair mismatches in vitro. *P Natl Acad Sci USA* **80**: 4639–4643.

Lynch M. 2010. Rate, molecular spectrum, and consequences of human mutation. *Proc Natl Acad Sci USA* **107**: 961–968.

Ma X, Shao Y, Tian L, Flasch DA, Mulder HL, Edmonson MN, Liu Y, Chen X, Newman S, Nakitandwe J, et al.. 2019. Analysis of error profiles in deep Next-Generation Sequencing data. *Genome Biol* **20**: 50.

Melamed D, Young DL, Gamble CE, Miller CR, and Fields S. 2013. Deep mutational scanning of an RRM domain of the *Saccharomyces cerevisiae* poly(A)-binding protein. *RNA* **19**: 1537–1551.

Ohno M, Sakumi K, Fukumura R, Furuichi M, Iwasaki Y, Hokama M, Ikemura T, Tsuzuki T, Gondo Y, and Nakabeppu Y. 2014. 8-oxoguanine causes spontaneous *de novo* germline mutations in mice. *Sci Rep* **4**: 1–9.

Orkin SH. 1990. Globin gene regulation and switching: circa 1990. *Cell* **63**: 665–672.

Potapov V and Ong JL. 2017. Examining sources of error in PCR by single-molecule sequencing. *PLoS One* **12**.

Rahbari R, Wuster A, Lindsay SJ, Hardwick RJ, Alexandrov LB, Al Turki S, Dominiczak A, Morris A, Porteous D, Smith B, et al.. 2016. Timing, rates and spectra of human germline mutation. *Nat Genet* **48**: 126.

Schmitt MW, Kennedy SR, Salk JJ, Fox EJ, Hiatt JB, and Loeb LA. 2012. Detection of ultra-rare mutations by Next-Generation Sequencing. *Proc Natl Acad Sci USA* **109**: 14508–14513.

Ségurel L, Wyman MJ, and Przeworski M. 2014. Determinants of mutation rate variation in the human germline. *Annu Rev Genomics Hum Genet* **15**: 47–70.

Serjeant G and Serjeant B. 2001. *Sickle Cell Disease*. Oxford University Press, Oxford, UK.

Shendure J and Akey JM. 2015. The origins, determinants, and consequences of human mutations. *Science* **349**: 1478–1483.

Steinberg M and Adams JI. 1991. Hemoglobin A2: origin, evolution, and aftermath. *Blood* **78**: 2165–2177.

Tsai SQ and Joung JK. 2016. Defining and improving the genome-wide specificities of CRISPR–Cas9 nucleases. *Nat Rev Genet* **17**: 300.

Tubbs A and Nussenzweig A. 2017. Endogenous DNA damage as a source of genomic instability in cancer. *Cell* **168**: 644–656.

Turk PW and Weitzman SA. 1995. Free radical DNA adduct 8-oh-deoxyguanosine affects activity of Hp a II and Msp I restriction endonucleases. *Free Radical Res* **23**: 255–258.

Turner TN, Coe BP, Dickel DE, Hoekzema K, Nelson BJ, Zody MC, Kronenberg ZN, Hormozdiari F, Raja A, Pennacchio LA, et al.. 2017. Genomic patterns of *de novo* mutation in simplex autism. *Cell* **171**: 710–722.

Wang K, Ma Q, Jiang L, Lai S, Lu X, Hou Y, Wu CI, and Ruan J. 2016. Ultra-precise detection of mutations by droplet-based amplification of circularized DNA. *BMC Genom* **17**: 214.

Wang RYH, Kuo KC, Gehrke CW, Huang LH, and Ehrlich M. 1982. Heat and alkali-induced deamination of 5-methylcytosine and cytosine residues in DNA. *BBA Gene Struct Expr* **697**: 371–377.

Wood ML, Dizdaroglu M, Gajewski E, and Essigmann JM. 1990. Mechanistic studies of ionizing radiation and oxidative mutagenesis: genetic effects of a single 8-hydroxyguanine (7-hydro-8-oxoguanine) residue inserted at a unique site in a viral genome. *Biochemistry* **29**: 7024–7032.

Yates A, Achuthan P, and Akanni W. 2020. Ensembl 2020. *Nucleic Acids Res* **48**: D682–D688.

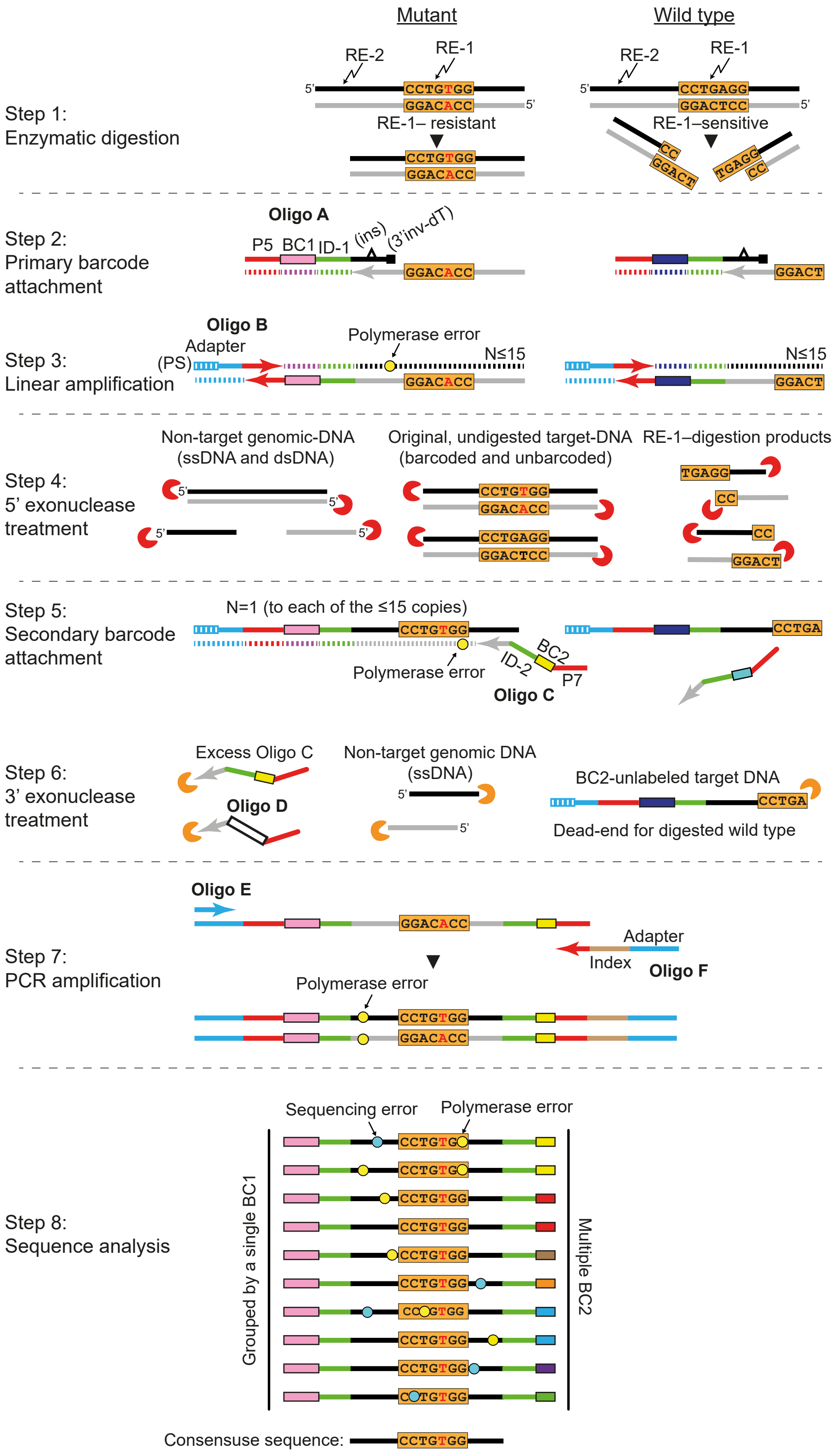
Mutant
Wild type
Step 1:
Enzymatic digestion
RE-2
RE-1
RE-1– resistant
RE-1–sensitive
Step 2:
Primary barcode attachment
Oligo A
P5
BC1
ID-1
(ins)
(3'inv-dT)
Step 3:
Linear amplification
Oligo B
Adapter
(PS)
Polymerase error
N≤15
Step 4:
5' exonuclease treatment
Non-target genomic-DNA (ssDNA and dsDNA)
Original, undigested target-DNA (barcoded and unbarcoded)
RE-1–digestion products
Step 5:
Secondary barcode attachment
N=1 (to each of the ≤15 copies)
Polymerase error
BC2
ID-2
P7
Oligo C
Step 6:
3' exonuclease treatment
Excess Oligo C
Oligo D
Non-target genomic DNA (ssDNA)
BC2-unlabeled target DNA
Dead-end for digested wild type
Step 7:
PCR amplification
Oligo E
Adapter
Index
Oligo F
Polymerase error
Step 8:
Sequence analysis
Sequencing error
Polymerase error
Grouped by a single BC1
Multiple BC2
Consensuse sequence:

Figure S1: An illustration of the MEMDS method. Step 1) Enzymatic digestion of genomic DNA. Restriction Enzyme 1 (RE-1) digests a region of interest (ROI) with a wild-type sequence and is blocked by a mutation at this site (the mutation is marked by a red letter). Restriction Enzyme 2 (RE-2) digests in close proximity to the ROI. Step 2) A fill-in reaction is promoted by oligo A, which anneals to the sequence between the RE-1 and RE-2 sites and introduces directly to the target DNA strand (gray strand) a sample-identifier sequence (ID-1) common to all labeled sequences in the sample, a primary barcode sequence (BC1) that is unique to each target DNA molecule and an Illumina-P5 sequence. Shown also are the 3' inverted-dT (3'inv-dT) that blocks oligo A extension and the control base-insertion (ins) for the identification and removal of extension products from unblocked oligo A at the sequence analysis step. Step 3) Linear amplification of the barcoded target molecules is carried out for 15 cycles using oligo B that anneals to the P5 sequence of the target DNA strand and adds an Illumina adapter sequence with 5 phosphorothioate bonds at the 5' edge (5'PS) of each barcoded-ROI copy. This linear amplification reaction results in 15 or less copies of each barcoded target molecule ($N \leq 15$). While polymerase errors (marked by yellow circles) do occur, they are unlikely to repeat themselves at the same position in multiple copies of the same target molecule. Step 4) A mixture of 5' exonucleases (red pacman) is added to degrade from 5' to 3' non-target genomic DNA including RE-1 and RE-2 digestion products. The barcoded copies are protected from this degradation step due to their 5'PS bonds. Step 5) A single-extension reaction with oligo C is carried out in order to add to each linearly amplified copy of a BC1-labeled target molecule an additional sample identifier sequence (ID-2), a unique secondary barcode sequence (BC2), and an Illumina-P7 sequence. For each target molecule, this step results in 15 or less copies that share the same primary barcode at one end, while having a unique secondary barcode at the other end. Since oligo C anneals 3' to the ROI sequence, any linearly amplified copy of an RE-1 digestion product cannot be labeled by this oligo. Step 6) A 3' exonuclease (orange pacman) is added immediately after the single-extension reaction to degrade from 3' to 5' any single-stranded DNA, including excess of oligo C, to prevent secondary barcode relabeling during the next PCR reaction. Copies labeled by secondary barcodes are protected from this degradation step due to their double-stranded state, while unlabeled copies are single-stranded and are therefore degraded. A relabeling-control primer (oligo D), carrying a unique sequence signature, is added in known amount together with the 3' exonuclease to assess, at the sequence analysis step, the number of oligo C relabelings in the event of incomplete degradation of oligo C by the 3' exonuclease. Step 7) PCR amplification completes the final sequence requirements for Illumina HTS and produces a library of barcoded ROI sequences composed of enriched mutation variants as well as wild-type sequences that escaped RE-1 digestion. Step 8) Following high-throughput sequencing, reads are grouped into families based on their primary barcode sequences, so that within each family, all members have the same primary barcode, and the consensus sequence for the family is determined using three parameters: family size, mutation frequency, and the number of secondary barcodes associated

with each base. This procedure allows us to eliminate PCR errors (yellow circles) and HTS errors (blue circles), which usually appear in low frequencies and are linked to single secondary barcodes, and to accept as true, *de novo* mutations only mutations that appear in multiples reads and are associated with multiple secondary barcodes, such as the A$\rightarrow$T substitution in the figure.

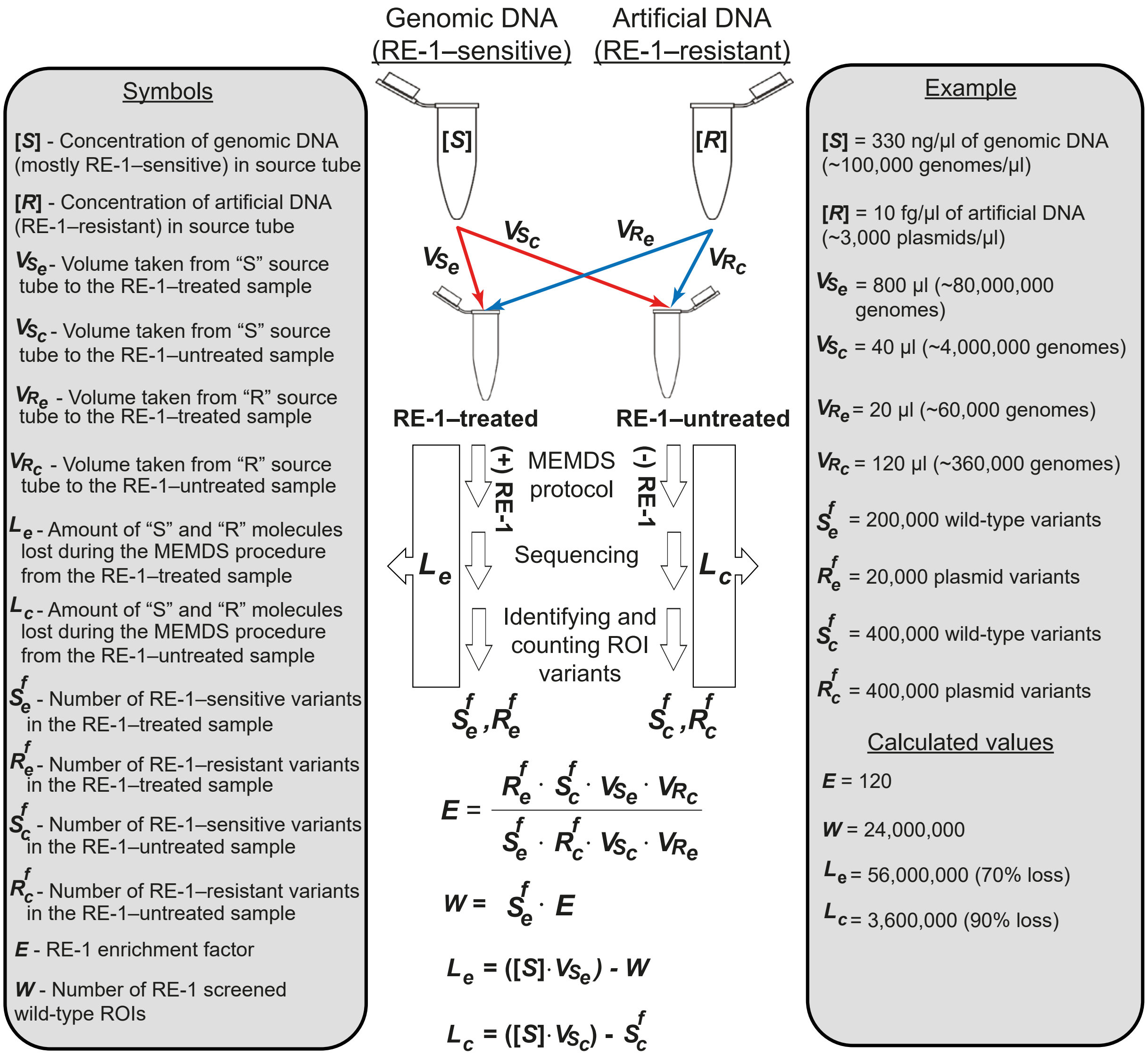

$$E = \frac{R^f_e \cdot S^f_c \cdot VS_e \cdot VR_c}{S^f_e \cdot R^f_c \cdot VS_c \cdot VR_e}$$

$$W = S^f_e \cdot E$$

$$L_e = ([S] \cdot VS_e) - W$$

$$L_c = ([S] \cdot VS_c) - S^f_c$$

Figure S2: MEMDS Experimental design to calculate the RE-1–enrichment factor and the number of target DNA molecules digested by RE-1. Two tubes, one containing genomic DNA that carries mostly RE-1–sensitive ROI sequences, denoted S, and one containing artificial-ROI sequences resistant to RE-1 digestion, denoted R, are used as source tubes from which volumes are drawn in known amounts in order to create two mixtures of the two samples, designated "RE-1–treated" and "RE-1–untreated" samples (see left panel for the abbreviations used). These two samples undergo the full MEMDS protocol, with the exception that the former is treated with and the latter without RE-1. Following high-throughput sequencing, variants are identified by the MEMDS computational pipeline and the numbers of RE-1–sensitive ROI

variants (i.e., wild-type ROIs) and artificial RE-1–resistant ROI variants are determined for each sample (S_e^f and R_e^f denote the numbers of sensitive and resistant variants identified for the RE-1 treated sample, and S_c^f and R_c^f denote the sensitive and resistant variants identified for the RE-1 untreated sample). These numbers, together with the known volumes taken from the source tubes to create the input mixtures, are used to calculate the RE-1–enrichment factor, E, and the total number, W, of wild-type sequences that were either digested by RE-1 or evaded digestion and were sequenced and identified. The right panel shows an example for the calculation of E and W based on DNA concentrations in each source tube and volumes taken for the input mixtures that are similar to the DNA concentrations and volumes used in our MEMDS experiments but are rounded for the sake of simplicity here. In this example, the RE-1–enrichment factor equals 120, meaning that *de novo* mutations in the ROI, which block RE-1 similarly to the artificial sequences, are enriched 120-fold in the RE-1–treated sample compared to the RE-1–untreated sample. Using this enrichment factor we find that the total number of unique wild-type molecules screened by the MEMDS procedure is 24,000,000, which includes the number of wild-type target molecules in the RE-1– treated sample that were digested by RE-1 and the 400,000 RE-1–sensitive variants that escaped digestion and were sequenced. Note that the calculation of E and W relies only on the number of original target molecules that were sequenced in the computational analysis step and on the volumes used to generate the input mixtures, and therefore the number of genomes and artificial sequences in the source tubes is not needed for it. Yet, by having a rough estimate of the actual amount of DNA transferred from the source tube, one can assess the number of target DNA molecules (either genomic or artificial ROI-including molecules) that were lost during the MEMDS procedure (not due to RE-1 digestion but due to general loss of material involving the efficiencies of labeling, amplifying, purifying, capturing and sequencing all target sequences).

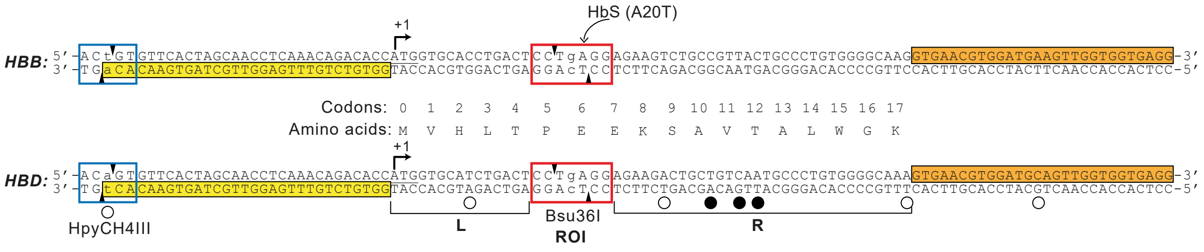

Figure S3: *HBB* and *HBD* sequence features. The double-stranded 114 bp DNA segments from the first exon of *HBB* (upper sequences) and the paralogous region of *HBD* (lower sequences) are shown. The mRNA-translation start sites (ATG) are marked by black arrows. For each gene, the upper sequence is in the sense orientation and the lower, antisense, complementary sequence served as the target DNA strand, which was barcoded and subsequently amplified by the MEMDS protocol. Positions that vary between the two genes are marked by circles below the *HBD* segment. Positions marked by filled circles were used to sort HTS reads from the same sperm-DNA sample to separate *HBB* and *HBD* datasets at the sequence analysis step, as the two genes were barcoded and amplified simultaneously by the MEMDS procedure. The Bsu36I (RE-1)-recognition sequence is marked by a red frame and its cleavage sites are marked by small black triangles. Position 20, where the HbS (20A→T) mutation occurs, is marked by a curved arrow. The base denoted by a lower-case letter in the center of the Bsu36I site can tolerate any substitution without affecting Bsu36I activity. Therefore, the region of interest (ROI) is confined to six of the seven bases in the red frame that constitute the Bsu36I site. The HpyCH4III (RE-2)-recognition sequence is marked by a blue frame and its cleavage sites are marked by small black triangles. The base denoted by a lower-case letter in the HpyCH4III site can tolerate any substitution without affecting HpyCH4III activity. The sequence in the yellow box anneals to oligo A and receives the primary barcode via a single, fill-in reaction (see Figure S1, step 2). Note that the first base that primes this extension, marked by a lower-case letter, differs between *HBB* and *HBD*. Therefore, we used a mixture of oligo A sequences that carry either one of the two complementary bases to minimize any bias due to delayed extension by the Q5 DNA polymerase. The sequence in the orange box anneals to oligo C and receives the secondary barcode via a single extension reaction (see Figure S1, step 5). The sequence between oligo A and oligo C remains untouched by any primer and therefore is suitable for mutation detection analysis. Yet, only mutations at the ROI can be enriched, while mutations in the flanking right (R) and left (L) sequences are unlikely to affect Bsu36I digestion.

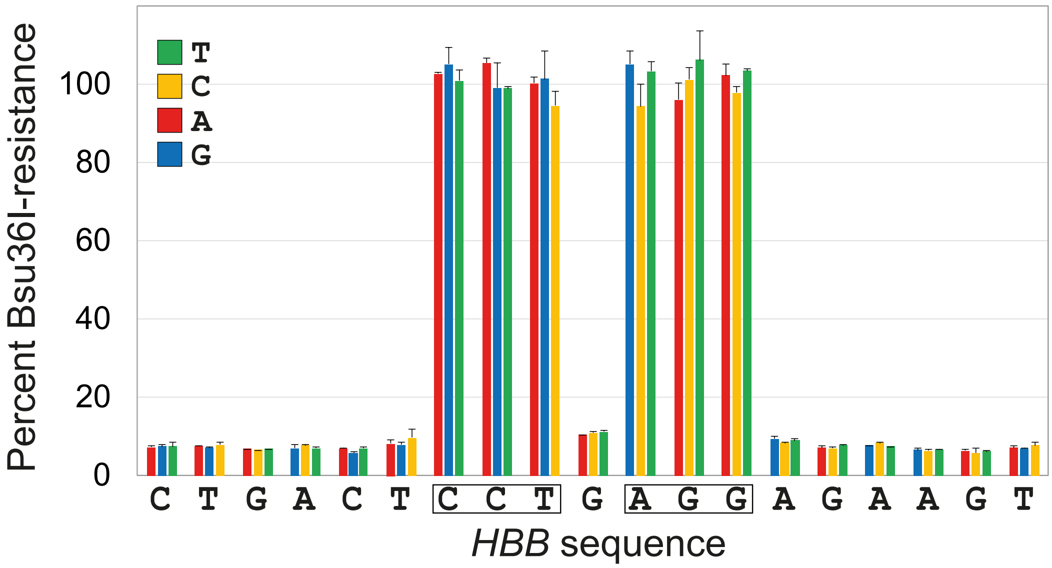

Figure S4: Single-base substitutions protect from Bsu36I digestion similarly to the MEMDS artificial ROI variant that carries multiple changes in the Bsu36I site. A synthetic dsDNA library of *HBB* gene segments containing the Bsu36I-restriction site with its flanking sequences and a single point mutation per segment was divided into two and sequenced following incubation with or without Bsu36I. As Bsu36I digestion results in the depletion of sequences that are Bsu36I-sensitive, the frequency of each full-length variant in the post-Bsu36I treatment pool compared to its frequency in the pre-Bsu36I treatment pool was used to determine its degree of Bsu36I-resistance. Changes in frequencies were normalized to the change in frequency of an artificial variant with multiple substitutions in the Bsu36I site, which was set to 100% resistance (n=2). The six bases that constitute the *HBB* ROI are shown in boxes and the identities of the substituting bases are color-coded. Note that Bsu36I-sensitive variants are not completely depleted from the post-Bsu36I treatment pool, probably due to Bsu36I-resistant heteroduplex DNA that carry a Bsu36I-sensitive sequence in one strand and a Bsu36I-resistant sequence in the second strand, formed during the PCR reaction that generated the input dsDNA library.

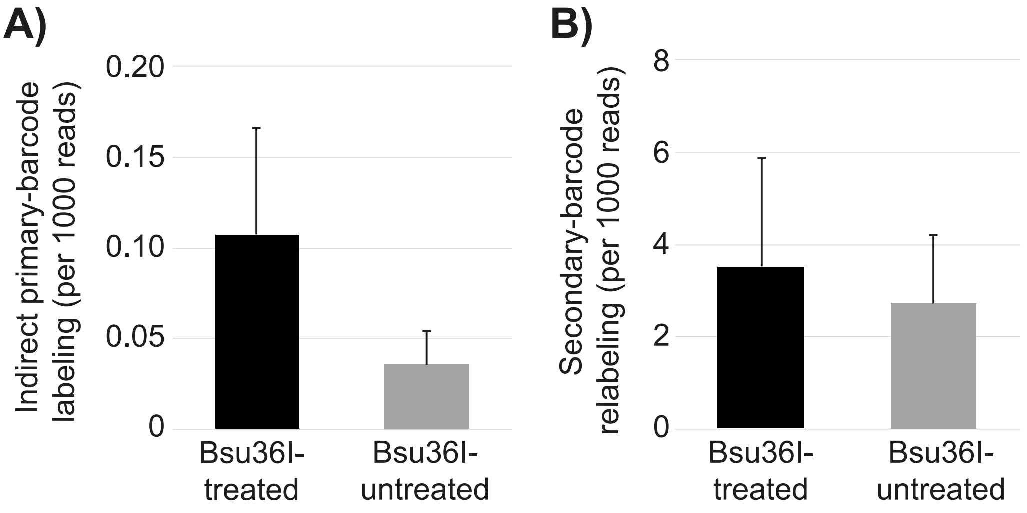

Figure S5: Frequency of erroneous barcode labelings. A) Frequency of indirect labeling by the primary barcode oligo (oligo A) as measured by the fraction of reads carrying the control-guanine insertion. B) Frequency of secondary barcode primer (oligo C) relabeling as measured by the relative frequency of reads carrying the sequence signature of the control secondary barcode relabeling primer (oligo D).

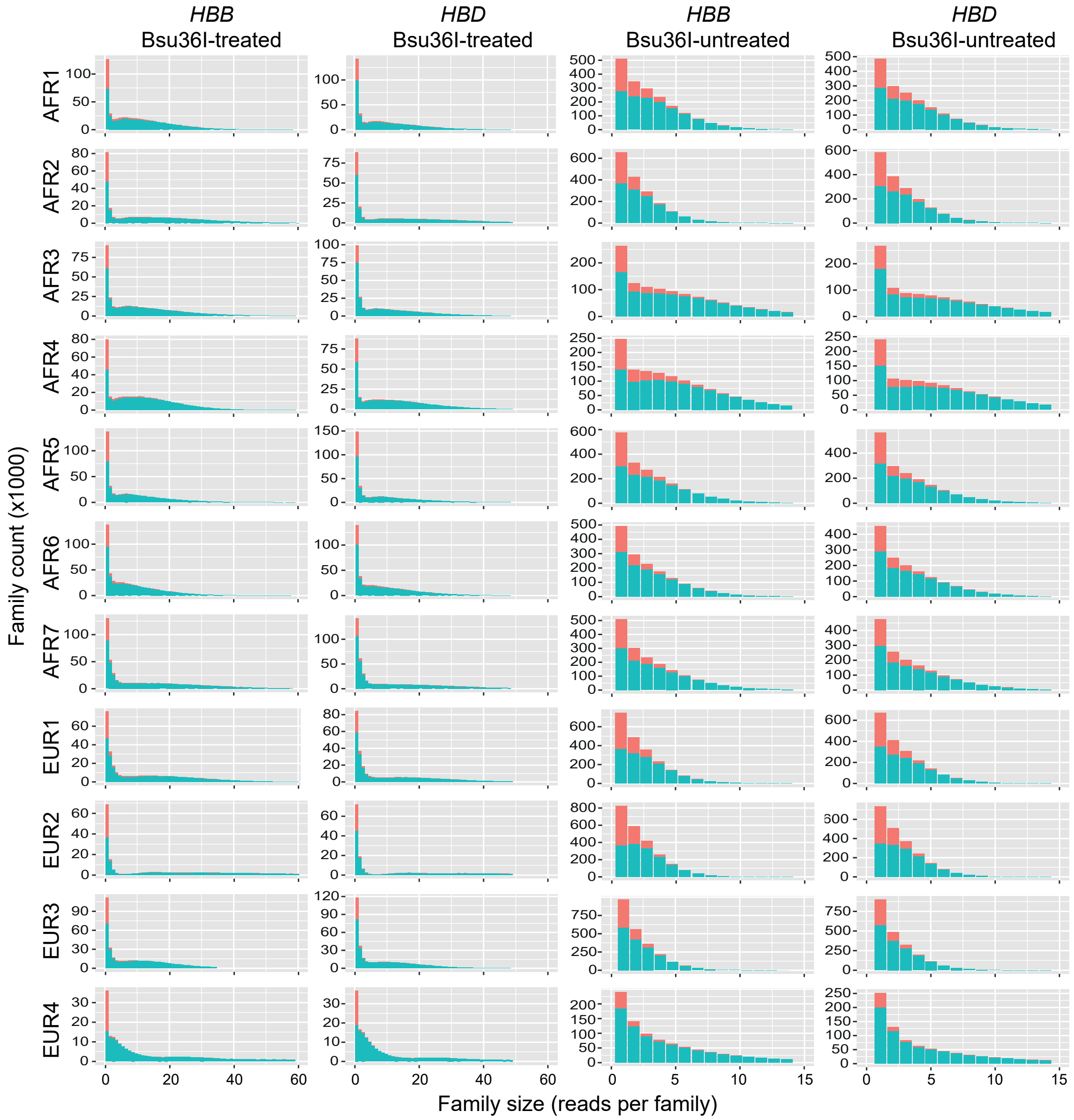

Figure S6: Family-size distributions. Distributions of primary barcode families based on the number of read in a family (family size). In red: counts of families with primary barcode sequences that deviate by a Hamming distance of one from primary barcode sequences of families with a greater number of reads. In green: counts of families with primary barcode sequences that deviate by a Hamming distance > 1 from primary barcode sequences of families with a greater number of reads. Note the different scales used for the Bsu36I-untreated and treated samples. (The differences in family size between the two treatments are merely due to the higher recovery of ROI families in the Bsu36I-untreated samples, which lack depletion of wild-type sequences.)

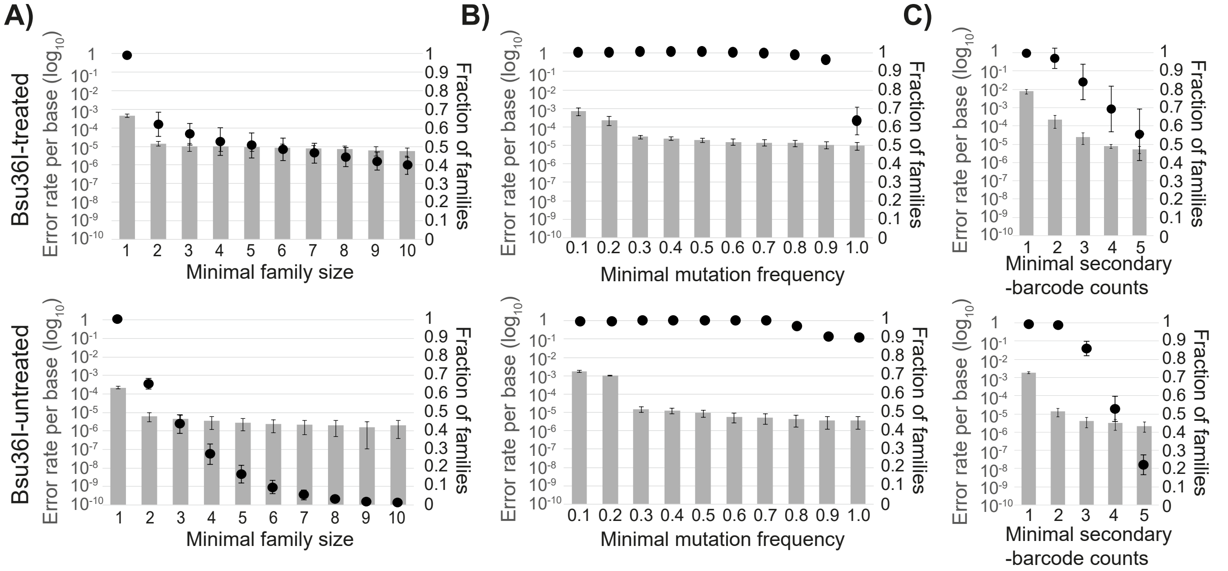

Figure S7: Effects of various cutoff criteria on mutation-calling accuracy. Upper row: average values from the Bsu36I-treated samples of AFR1, AFR2, EUR1, EUR2. Lower row: average values from the Bsu36I-untreated samples of the same donors . Bar graphs: error rate per base on a log-10 scale (left axis) while varying each cutoff criterion alone, calculated for the 47 bp that constitute the *HBB* and *HBD* ROI-flanking sequences for the Bsu36I-treated samples and for the 54 bp that constitute the ROI and the flanking sequences for the Bsu36I-untreated samples. Chimeric *HBB*/*HBD* markers (*HBB* 9C→T and *HBD* 9T→C substitutions) were not included in the mutation count (Supplemental Section 9), nor was the ROI-flanking sequence mutation 14C→A that was found to be enriched by Bsu36I digestion (Fig. S13 and Supplemental Section 8). Dot plots: Fraction of *HBB* and *HBD* families that meet a cutoff criterion (right axis). A) The effect of increasing the family-size cutoff. Mutations present at 100% of the sequences in a primary barcode family were selected for the mutation-rate calculation. B) The effect of increasing the mutation-frequency cutoff for families with at least four reads. C) The effect of increasing the secondary barcode count cutoff for families with at least four reads.

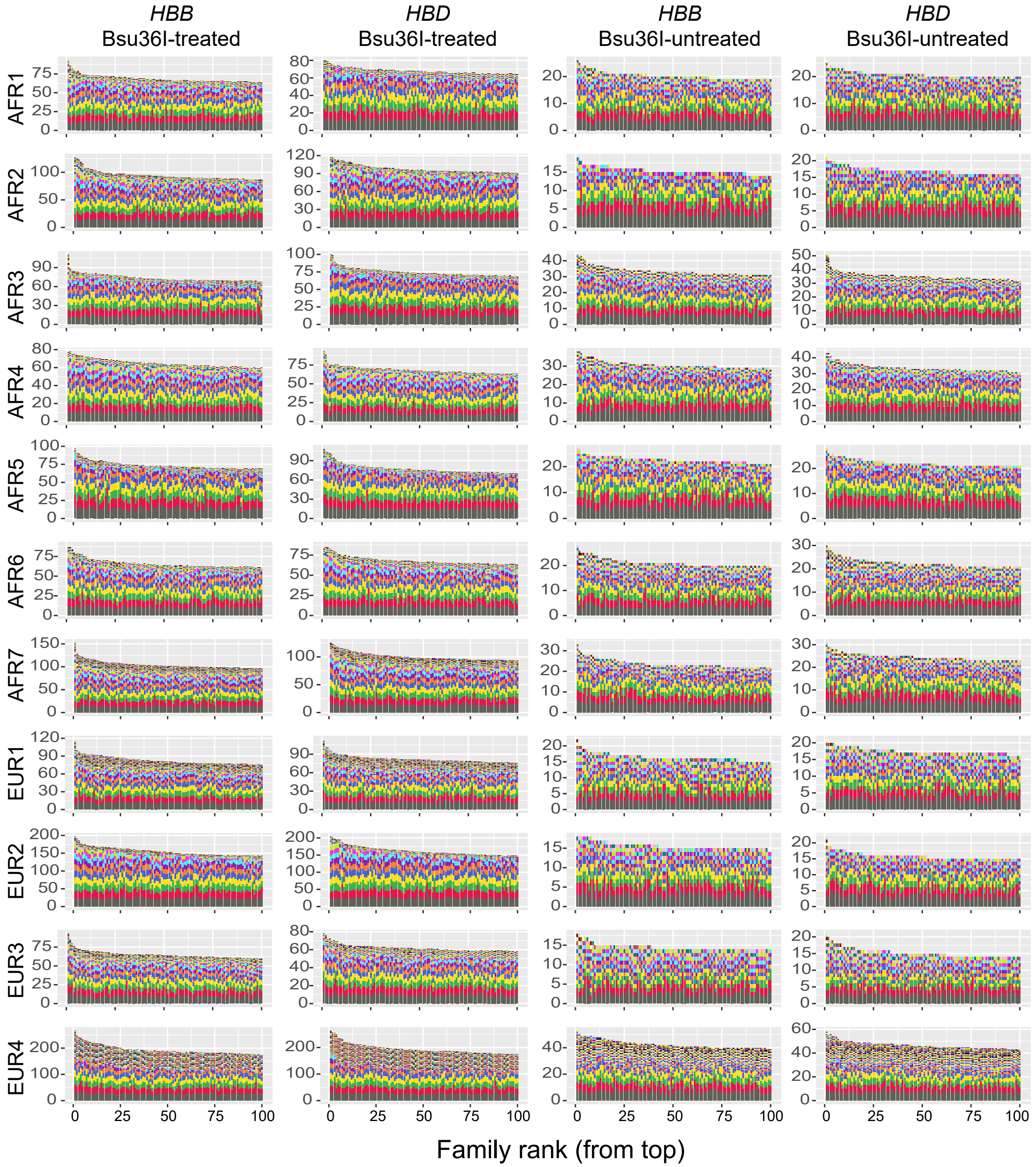

Figure S8: Secondary barcode distribution. Distribution of secondary barcode counts in the top 100 families with the highest read counts. Unique secondary barcodes are marked by different colors. The family-size axes were adjusted to the families with the highest read counts.

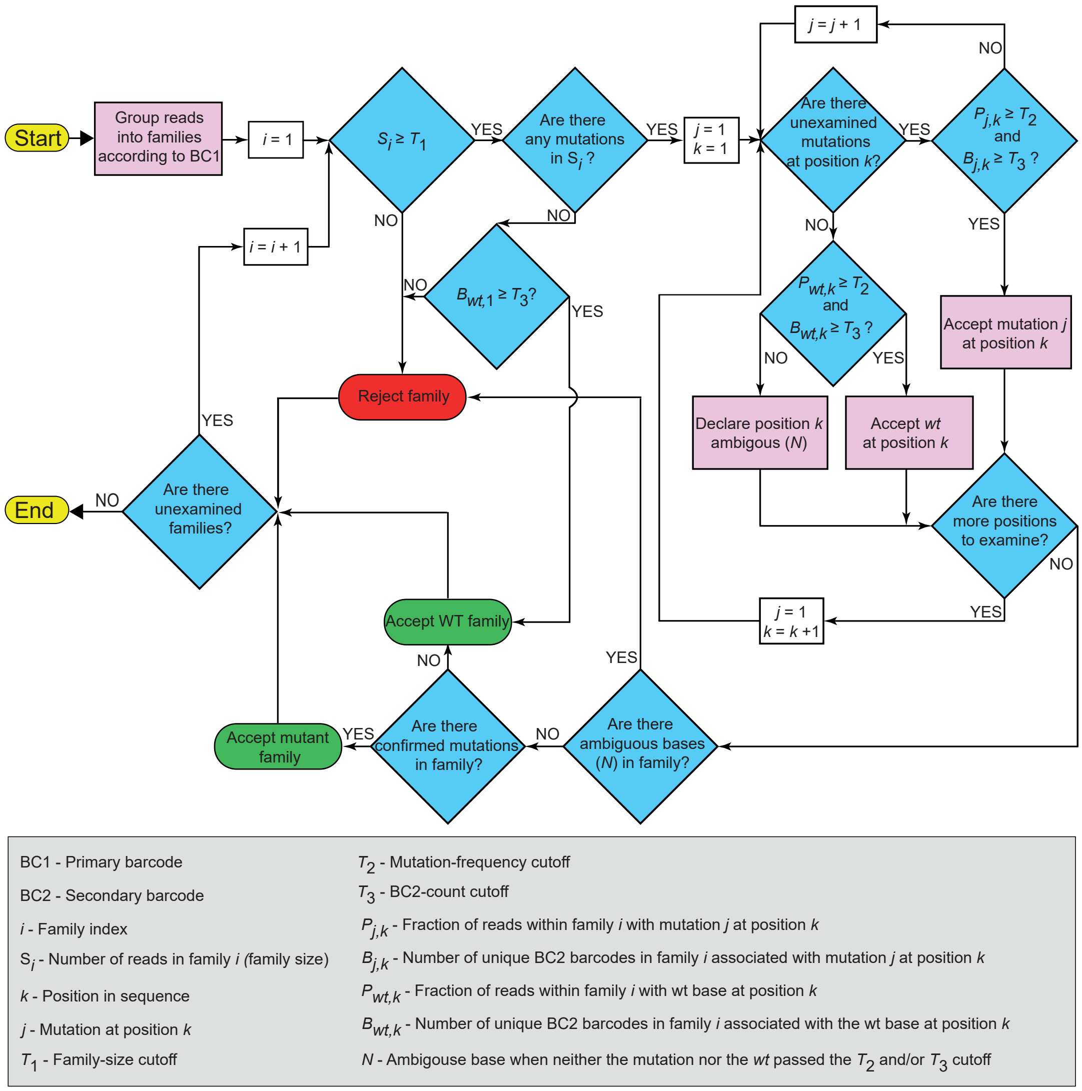

Figure S9: An illustration of the MEMDS computational pipeline. The workflow describes the computational analysis from the point of grouping reads into families by their shared primary barcodes, where each family represent a single target-DNA molecule, to the characterization of each family by its mutations that pass the combined cutoff criteria, if they exist. These criteria include a minimal family size of four reads (T_1), a mutation frequency cutoff of at least 0.7 (T_2) and the association of the particular mutation called with at least two secondary barcodes (T_3). Note that for any given mutated position, in the case of failure to pass the combined cutoff criteria, the wild-type base is tested by the same conditions to validate its authenticity in an unbiased manner. If both the mutation and the wild-type base fail to meet the cutoff criteria, the base identity at that position is declared ambiguous (N), and the family is rejected.

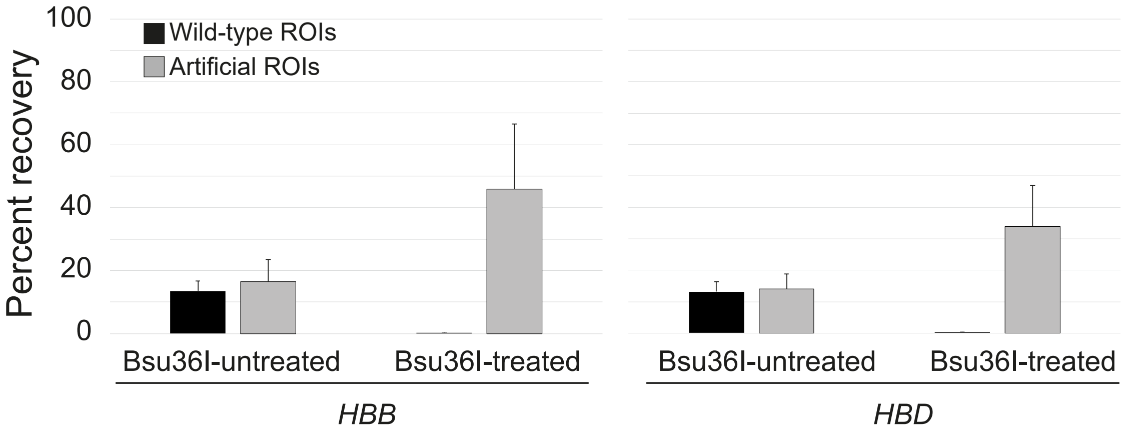

Figure S10: Percent recoveries of WT (genomic) sequences and artificial (plasmid) sequences by the MEMDS method. Percent recoveries of the WT and artificial target molecules were calculated using the ratios between the obtained number of families of each type and the estimated amounts of input families derived from their DNA concentration measurements.

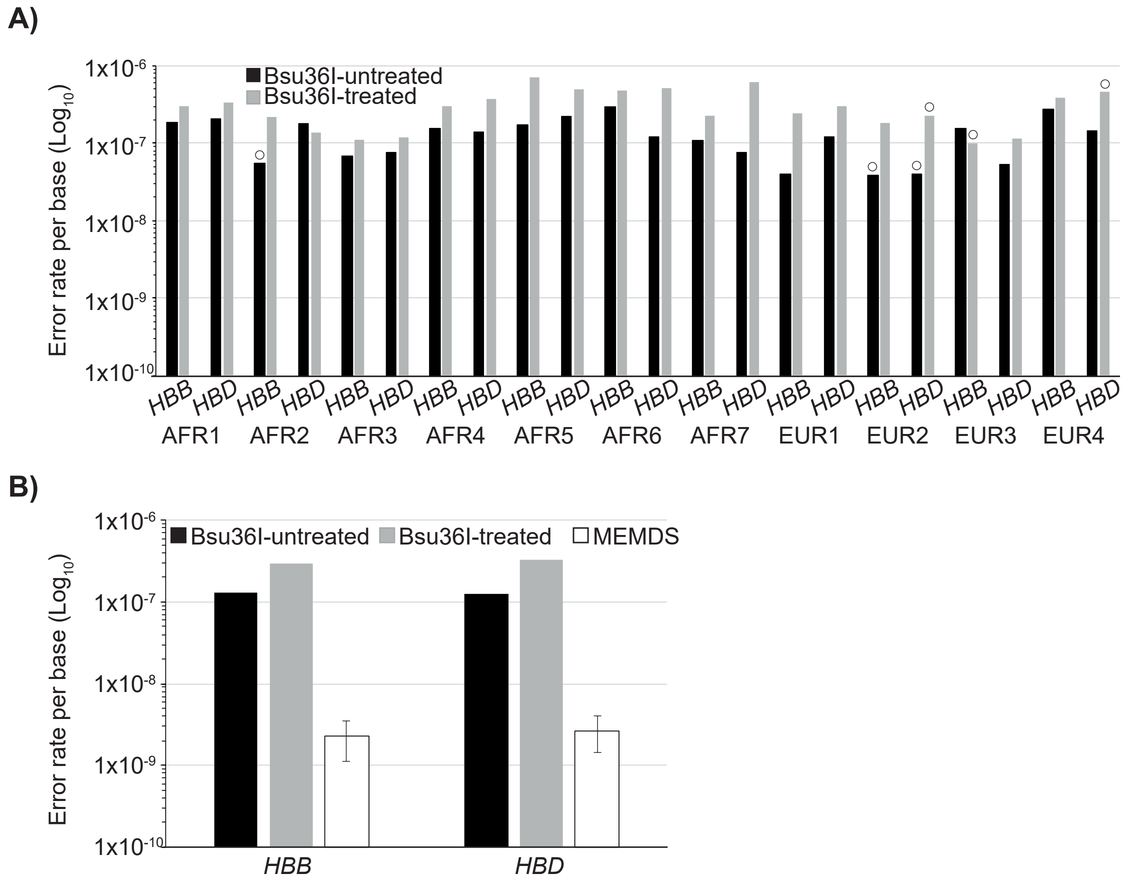

Figure S11: Calculated error rates. A) Per base error rates for non G→T, C→T and C→A mutations (in the target DNA strand) were calculated for each donor for the 47 bp that include the ROI-flanking sequences in the Bsu36I-untreated samples (black bars) and Bsu36I-treated (gray bars) samples, under the stringent assumption that all mutations observed in these unenriched sequences are errors. Open circles mark samples where no non-G→T, C→T and C→A mutations were observed and the error rate calculation for these samples used a theoretical mutation count value of 1. B) The total error rate for each gene (i.e., the sum of non-G→T, C→T and C→A mutations for that gene across all donors divided by the total number of bases) calculated for the same sequences depicted in S11A. The MEMDS error rates for the 6 bases that constitute the ROI were calculated for each donor by dividing the total error rate achieved for the ROI-flanking sequences of the Bsu36I-treated samples by the relevant Bsu36I-enrichment factor.

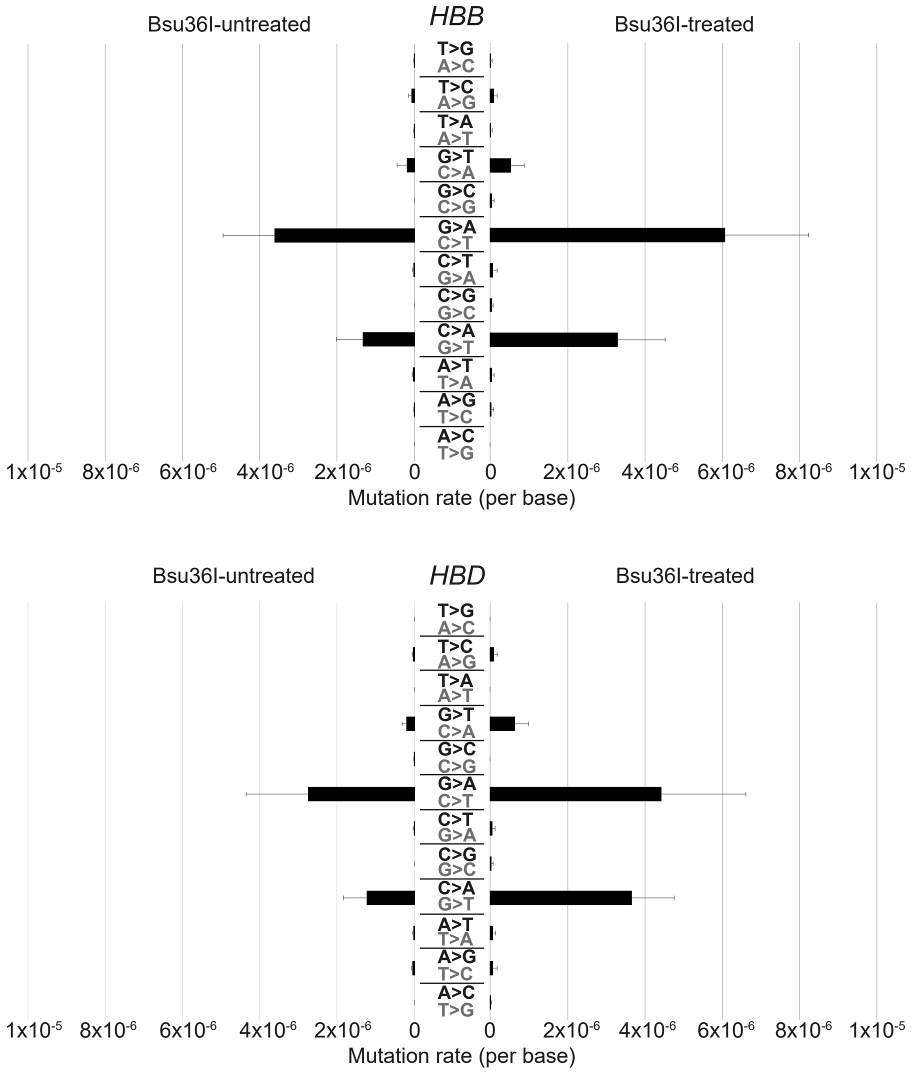

Figure S12: Per type point mutation frequencies. Average point mutation frequencies were calculated for the 47 bp that include the ROI-flanking sequences in the Bsu36I-treated and untreated samples. The chimeric *HBB*/*HBD* markers (*HBB* 9C→T and *HBD* 9T→C substitutions) were not included in the mutation count (Fig. S13 and Supplemental Section 9), nor was the ROI-flanking sequence mutation 14C→A that was found to be enriched by Bsu36I digestion (Fig. S13 and Supplemental Section 8). In gray: mutations in the target (antisense) DNA strand. In black: the complementary mutations in the sequenced (sense) strand.

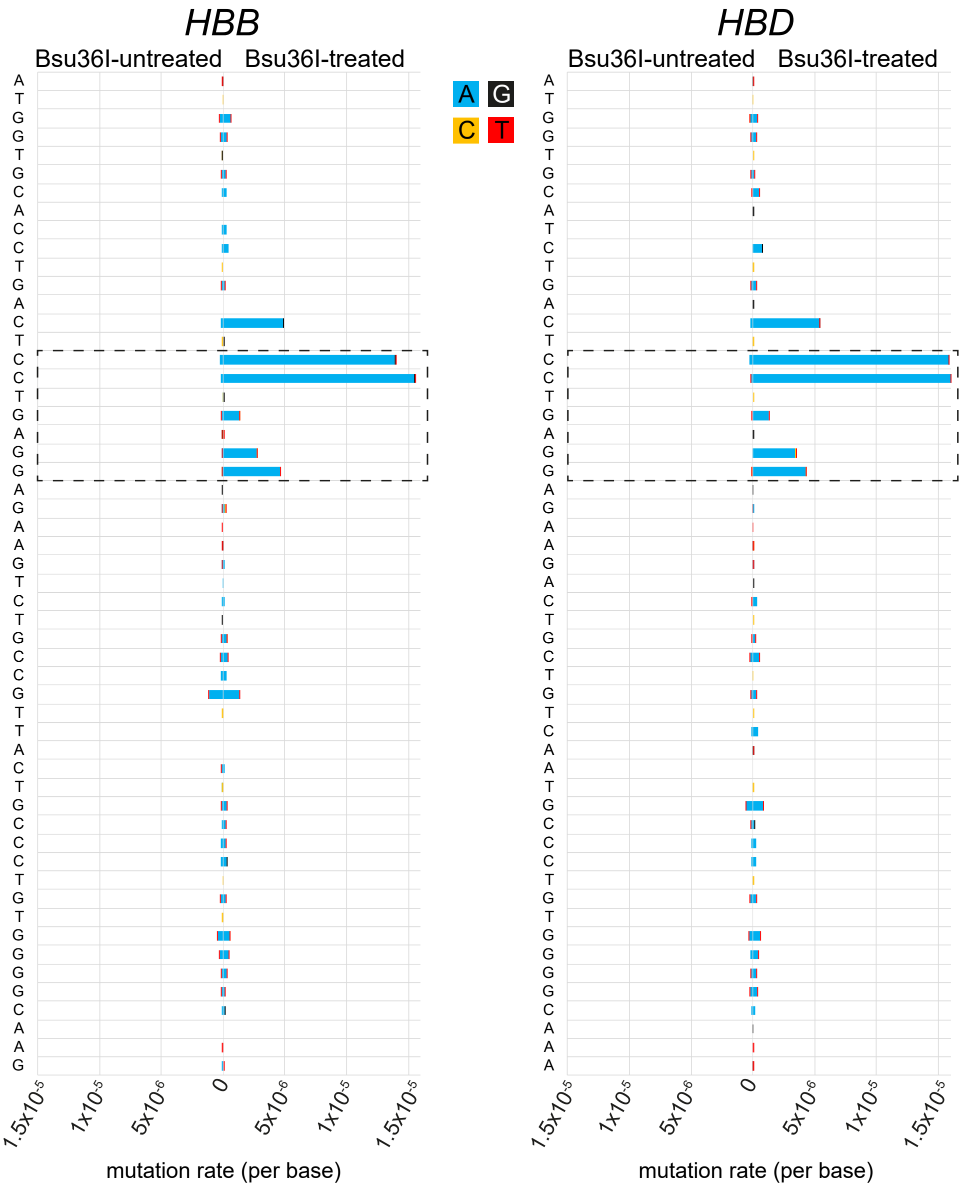

Figure S13: Mutation distribution in *HBB* and *HBD* sequences. Shown are the total mutation frequencies in *HBB* (left) and *HBD* (right) sequences of all 11 samples. Frequencies of mutations from the Bsu36I-treated and untreated samples are displayed in opposite directions. The Bsu36I restriction site, six of whose seven bases define the ROI, is boxed by dashed lines. Mutation frequencies for the 9C→T substitution in *HBB* and 9T→C in *HBD* were omitted due to their possible chimeric origin. Note that both *HBB* and *HBD* sequences are shown in the sense orientation, which corresponds to the sequencing output data. Since this MEMDS experiment targeted the antisense strand of both genes, the mutations in the target DNA molecules were the reciprocals of the mutations shown here.

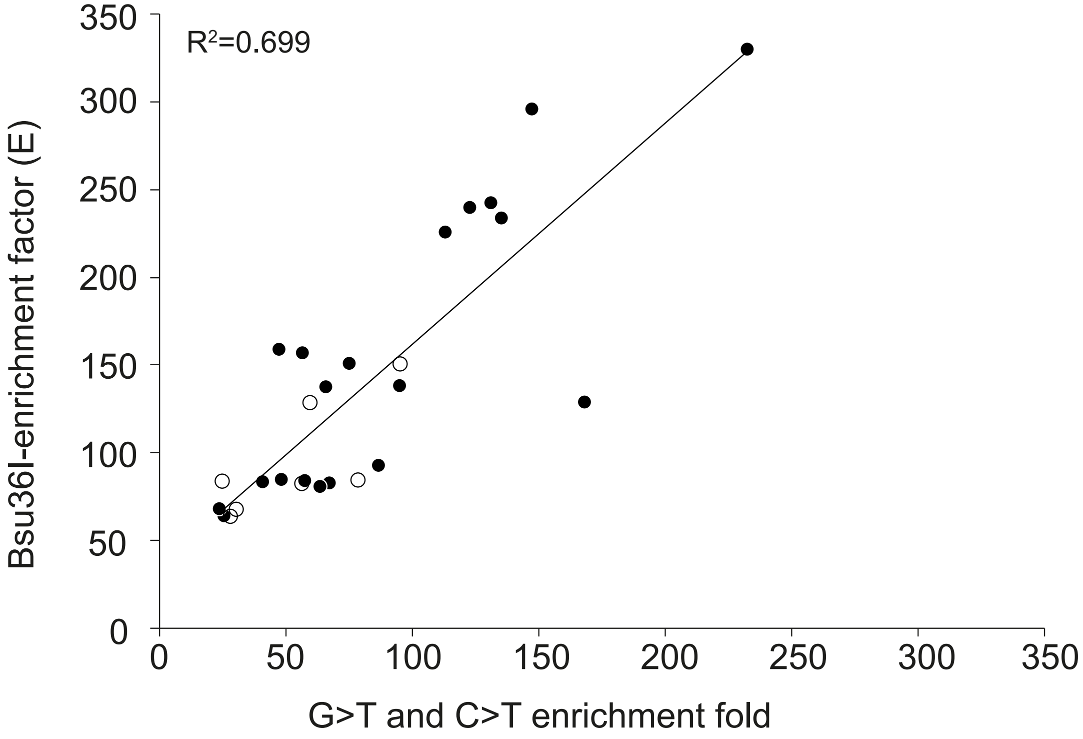

Figure S14: Correlation between the enrichments of target-strand G→T and C→T mutations and the Bsu36I-enrichment factors. The fold enrichment of the ROI G→T (filled circles) and C→T (open circles) mutations (C→A and G→A mutations in the sequence data, respectively) was determined by the ratio between the mutation frequencies in the ROI of the Bsu36I-treated and untreated samples. For each mutation type data is shown only for donors with at least 3 mutation counts in the ROI site.

Donor	Donation year	Parent-1/Parent-2 origin	Age group	Ethnic group	Sperm count (millions/mL)	% motility
AFR1	2018	Ghanaian/Ghanaian	23-28	Akan	46.1	45
AFR2	2018	Ghanaian/Ghanaian	35-39	Ewe	27.6	38
AFR3*	2018<br>2018	Ghanaian/Ghanaian<br>Ghanaian/Ghanaian	23-28<br>23-28	Ga/Ewe<br>Dagbani	24.1<br>20	43<br>40
AFR4	2018	Ghanaian/Ghanaian	18-22	Akan	30.5	46
AFR5	2019	Ghanaian/Ghanaian	23-28	Akan	51.1	42
AFR6	2019	Ghanaian/Ghanaian	29-34	Akan	42.3	50
AFR7	2019	Ghanaian/Ghanaian	23-28	Ewe	45.3	36
EUR1	1994	English/German	23-28	Caucasian	68.0	50
EUR2	2001	English/English-Scandinavian	29-34	Caucasian	68.7	40
EUR3	2005/6	German/French-Italian	23-28	Caucasian	57.2	35
EUR4	1995	English/Norwegian	29-34	Caucasian	77.8	43

* Sample contains mixed cells from two donors

Table S1: General properties of sperm samples. Semen donations were received from 8 African (all Ghanaian) and 4 Northern European donors. AFR3 is a mixture of two samples from two separate donors that were combined into one. For simplicity of analysis, we consider it as one sample of mixed Ghanaian origins.

Donor	Treatment	Gene	Total Families[1]	Rejected due to low counts[2]	Rejected due to WT-secondary barcode failure[3]	Rejected due to shared primary barcodes[4]	Rejected due to ambiguous bases (N)[5]	Approved by the combined cutoff criteria[6]
AFR1	Bsu36I-treated	*HBB*	577,003	174,577	33,474	2,865	6,985	359,102
		HBD	492,594	189,270	31,969	2,865	7,390	261,110
	Bsu36I-untreated	*HBB*	1,876,444	1,154,913	5,796	4,569	3,052	708,114
		HBD	1,698,297	1,035,754	6,657	4,569	3,222	648,095
AFR2	Bsu36I-treated	*HBB*	326,152	105,610	12,819	2,326	2,910	202,487
		HBD	295,836	115,683	11,330	2,326	2,862	163,635
	Bsu36I-untreated	*HBB*	1,784,865	1,374,897	6,232	2,583	2,136	399,017
		HBD	1,753,711	1,259,296	5,899	2,583	2,344	483,589
AFR3	Bsu36I-treated	*HBB*	386,700	126,712	16,860	3,104	8,990	231,034
		HBD	349,216	137,830	18,259	3,104	5,435	184,588
	Bsu36I-untreated	*HBB*	1,148,389	497,865	9,729	3,725	5,900	631,170
		HBD	1,051,314	464,336	10,926	3,725	3,520	568,807
AFR4	Bsu36I-treated	*HBB*	426,278	107,727	19,883	987	3,122	294,559
		HBD	372,518	113,201	9,855	987	14,838	233,637
	Bsu36I-untreated	*HBB*	1,264,724	521,564	21801	4,290	4,563	712,506
		HBD	1,120,079	450,195	11,035	4,290	22,693	631,866
AFR5	Bsu36I-treated	*HBB*	463,684	185,935	15,604	4,054	29,396	228,695
		HBD	420,559	196,822	28,311	4,054	9,221	182,151
	Bsu36I-untreated	*HBB*	1,891,157	1,174,471	7,638	4,031	19,437	685,580
		HBD	1,746,131	1,103,189	14,760	4,031	5,622	618,529
AFR6	Bsu36I-treated	*HBB*	573,258	208,356	29,491	3,613	7,612	324,186
		HBD	531,781	200,664	22,825	3,613	5,782	298,897
	Bsu36I-untreated	*HBB*	1,575,956	1,014,909	3,497	3,292	3,864	550,394
		HBD	1,481,666	903,810	3,176	3,292	3,305	568,083
AFR7	Bsu36I-treated	*HBB*	574,043	212,633	10,969	32,028	3,850	314,563
		HBD	541,285	234,389	11,208	32,028	3,758	259,902
	Bsu36I-untreated	*HBB*	1,699,015	1,043,765	10,971	4,513	5,811	633,955
		HBD	1,555,197	932,933	9,599	4,513	5,004	603,148
EUR1	Bsu36I-treated	*HBB*	331,608	126,256	3,737	19,141	1,229	181,245
		HBD	311,712	139,262	3,252	19,141	1,422	148,635
	Bsu36I-untreated	*HBB*	2,144,051	1,589,762	2,462	4,199	2,161	545,467
		HBD	1,936,890	1,388,844	2,327	4,199	2,317	539,203
EUR2	Bsu36I-treated	*HBB*	228,770	89,463	7,512	4,692	2,623	124,480
		HBD	210,577	97,700	6,413	4,692	2,734	99,038
	Bsu36I-untreated	*HBB*	2,392,244	1,827,090	3,029	4,056	1,565	556,504
		HBD	2,172,098	1,619,546	3,392	4,056	1,725	543,379
EUR3	Bsu36I-treated	*HBB*	418,458	161,339	3236	7,226	7,428	239,229
		HBD	393,302	172,746	7,036	7,226	3,384	202,910
	Bsu36I-untreated	*HBB*	2,335,295	1,888,649	422	2,698	11,427	432,099
		HBD	2,143,059	1,712,093	778	2,698	3,942	423,548
EUR4	Bsu36I-treated	*HBB*	144,833	43,533	20,359	36	13,548	67,357
		HBD	122,042	38,710	18,175	36	11,712	53,409
	Bsu36I-untreated	*HBB*	731,846	242,928	122,442	1,102	26,355	339,019
		HBD	649,808	215,711	98,264	1,102	19,597	315,134

Table S2: Numbers of rejected and approved gene families following filtration by the combined cutoff criteria. 1. Total number of families subjected to the combined cutoff criteria. 2. Families failing to meet the family size cutoff of ≥ 4. 3. Wild-type families with at least 4 reads that fail to meet a secondary barcode count cutoff of ≥ 2. 4. Families that share their primary barcode sequences between *HBB* and *HBD* genes of the same donor and treatment (chimeric artifacts). 5. Mutation-containing families that fail to meet a mutation-frequency cutoff of ≥ 0.7 or a secondary barcode count cutoff of ≥ 2 for either a mutation or a wild-type

base at least in one position in sequence. 6. The combined cutoff criteria include a family size cutoff of ≥ 4, a mutation frequency cutoff of ≥ 0.7 and a secondary barcode count cutoff of ≥ 2. Note that the numbers of rejected and approved families sum up to the total number of families.

Donor	Treatment	Donor DNA Volume taken	Plasmid DNA Volume taken	Gene	WT families passing cutoff[1]	Plasmid families passing cutoff[2]	Enrichment factor[3]	Scanned target-sequences[4]
**AFR1** 380 ng/µl	Bsu36I-treated	700 µl (V_{S_e})	20 µl (V_{R_e})	*HBB*	350,937 (S_e^f)	3,657; 3,899 (R_e^f)	63.60	22,319,593
				HBD	254,507 (S_e^f)	2,939; 3,158 (R_e^f)	67.04	17,062,149
	Bsu36I-untreated	35 µl (V_{S_c})	120 µl (V_{R_c})	*HBB*	680,429 (S_c^f)	12,275; 15,367 (R_c^f)	1.00	680,429
				HBD	621,399 (S_c^f)	12,156; 14,492 (R_c^f)	1.00	621,399
**AFR2** 591 ng/µl	Bsu36I-treated	338 µl (V_{S_e})	20 µl (V_{R_e})	*HBB*	194,704 (S_e^f)	3,483; 3,803 (R_e^f)	158.56	30,872,266
				HBD	157,003 (S_e^f)	3,057; 3,200 (R_e^f)	156.05	24,500,318
	Bsu36I-untreated	16.9 µl (V_{S_c})	120 µl (V_{R_c})	*HBB*	388,011 (S_c^f)	4,682; 6,307 (R_c^f)	1.00	388,011
				HBD	469,182 (S_c^f)	6,077; 8,302 (R_c^f)	1.00	469,182
**AFR3** 355 ng/µl	Bsu36I-treated	744 µl (V_{S_e})	20 µl (V_{R_e})	*HBB*	194,315 (S_e^f)	4,179; 3917 (R_e^f)	150.09	29,164,431
				HBD	177,422 (S_e^f)	3,463; 3,227 (R_e^f)	128.13	22,696,345
	Bsu36I-untreated	37.2 µl (V_{S_c})	120 µl (V_{R_c})	*HBB*	610,803 (S_c^f)	10,760; 9,587 (R_c^f)	1.00	610,803
				HBD	549,390 (S_c^f)	10,267; 9,135 (R_c^f)	1.00	549,390
**AFR4** 355 ng/µl	Bsu36I-treated	720 µl (V_{S_e})	20 µl (V_{R_e})	*HBB*	286,939 (S_e^f)	3,561; 3,341 (R_e^f)	82.85	23,773,196
				HBD	227,197 (S_e^f)	2,931; 2,908 (R_e^f)	83.65	19,005,979
	Bsu36I-untreated	36 µl (V_{S_c})	120 µl (V_{R_c})	*HBB*	688,478 (S_c^f)	12,538; 11,448 (R_c^f)	1.00	688,478
				HBD	609,365 (S_c^f)	11,616; 10,849 (R_c^f)	1.00	609,365
**AFR5** 445 ng/µl	Bsu36I-treated	600 µl (V_{S_e})	20 µl (V_{R_e})	*HBB*	211,556 (S_e^f)	9,274; 7,321 (R_e^f)	91.87	19,436,031
				HBD	172,778 (S_e^f)	5,007; 3,830 (R_e^f)	83.92	14,500,198
	Bsu36I-untreated	30 µl (V_{S_c})	120 µl (V_{R_c})	*HBB*	621,848 (S_c^f)	37,351; 26,363 (R_c^f)	1.00	621,848
				HBD	576,349 (S_c^f)	25,761; 16,389 (R_c^f)	1.00	576,349
**AFR6** 894 ng/µl	Bsu36I-treated	320 µl (V_{S_e})	20 µl (V_{R_e})	*HBB*	305,108 (S_e^f)	9,731; 8,726 (R_e^f)	82.10	25,048,983
				HBD	285,210 (S_e^f)	7,152; 5,863 (R_e^f)	80.18	22,867,577
	Bsu36I-untreated	16 µl (V_{S_c})	120 µl (V_{R_c})	*HBB*	505,664 (S_c^f)	25,122; 19,589 (R_c^f)	1.00	505,664
				HBD	531,747 (S_c^f)	20,863; 15,454 (R_c^f)	1.00	531,747
**AFR7** 694 ng/µl	Bsu36I-treated	360 µl (V_{S_e})	20 µl (V_{R_e})	*HBB*	284,362 (S_e^f)	15,848; 13,647 (R_e^f)	137.55	39,114,652
				HBD	239,830 (S_e^f)	10,693; 8,789 (R_e^f)	136.97	32,848,562
	Bsu36I-untreated	18 µl (V_{S_c})	120 µl (V_{R_c})	*HBB*	581,338 (S_c^f)	29,950; 22,654 (R_c^f)	1.00	581,338
				HBD	563,058 (S_c^f)	22,790; 17,283 (R_c^f)	1.00	563,058
**EUR1** 446 ng/µl	Bsu36I-treated	592 µl (V_{S_e})	20 µl (V_{R_e})	*HBB*	173,119 (S_e^f)	3,696; 4,081 (R_e^f)	225.50	39,038,335
				HBD	141,078 (S_e^f)	3,460; 3,731 (R_e^f)	239.46	33,782,538
	Bsu36I-untreated	29.6 µl (V_{S_c})	120 µl (V_{R_c})	*HBB*	532,724 (S_c^f)	5,431; 7,304 (R_c^f)	1.00	532,724
				HBD	525,762 (S_c^f)	5,951; 7,479 (R_c^f)	1.00	525,762
**EUR2** 339 ng/µl	Bsu36I-treated	780 µl (V_{S_e})	20 µl (V_{R_e})	*HBB*	118,015 (S_e^f)	3,020; 3,231 (R_e^f)	329.27	38,858,799
				HBD	93,487 (S_e^f)	2,669; 2,712 (R_e^f)	340.41	31,823,910
	Bsu36I-untreated	39 µl (V_{S_c})	120 µl (V_{R_c})	*HBB*	545,961 (S_c^f)	4,499; 6,040 (R_c^f)	1.00	545,961
				HBD	532,567 (S_c^f)	4,788; 6,018 (R_c^f)	1.00	532,567
**EUR3** 633 ng/µl	Bsu36I-treated	440 µl (V_{S_e})	20 µl (V_{R_e})	*HBB*	210,009 (S_e^f)	15,676; 13,036 (R_e^f)	233.12	48,956,676
				HBD	183,054 (S_e^f)	10,589; 8,828 (R_e^f)	242.05	44,308,361
	Bsu36I-untreated	22 µl (V_{S_c})	120 µl (V_{R_c})	*HBB*	403,681 (S_c^f)	17,836; 10,574 (R_c^f)	1.00	403,681
				HBD	402,381 (S_c^f)	13,357; 7,803 (R_c^f)	1.00	402,381
**EUR4** 376 ng/µl	Bsu36I-treated	720 µl (V_{S_e})	20 µl (V_{R_e})	*HBB*	55,898 (S_e^f)	6,135; 5,215 (R_e^f)	295.65	16,526,517
				HBD	45,832 (S_e^f)	4,077; 3403 (R_e^f)	318.23	14,585,464
	Bsu36I-untreated	36 µl (V_{S_c})	120 µl (V_{R_c})	*HBB*	313,203 (S_c^f)	13,410; 12,402 (R_c^f)	1.00	313,203
				HBD	296,860 (S_c^f)	9,937; 8332 (R_c^f)	1.00	296,860

Table S3: Values for the calculation of Bsu36I-enrichment factors and numbers of scanned target DNA sequences. 1. Number of families with no mutations at the ROI that passed the

combined-cutoff criteria. 2. Number of families of plasmid DNA (two plasmids per a tested gene) that passed the combined-cutoff criteria. 3. Bsu36I enrichment factor, calculated by the volumes and family counts shown in the table using the formula shown in Supplemental Section 2. 4. For the Bsu36I-treated samples, this number includes both the sequenced and the Bsu36I-digested WT target sequences, which are computed by multiplying the number of WT families by the enrichment factor. For the Bsu36I-untreated samples, this number includes only the sequenced families.

Change description		*De novo* mutation-based		ERV-based, 3-mer		ERV-based, 5-mer		ERV-based, 7-mer	
Mutation	Name (HbVar)	GWA rate ($x10^{-8}$)	Fold change	GWA rate ($x10^{-8}$)	Fold change	GWA rate ($x10^{-8}$)	Fold change	GWA rate ($x10^{-8}$)	Fold change
16C>G	Hb Gorwihl	0.21	*7.4	0.27	5.8	0.28	5.7	0.36	4.4
16C>T	Hb Tyne	0.73	2.2	0.78	2.0	0.97	1.6	0.79	2.0
17C>G	Hb Warwickshire	0.21	***12.3	0.27	***9.7	0.17	***15.1	0.16	***16.4
17C>T	Hb Aix-les-Bains	0.73	2.2	0.84	1.9	0.66	2.4	0.60	2.6
18T>G		0.17	3.1	0.17	3.1	0.16	3.3	0.16	3.2
18T>A		0.14	3.8	0.13	4.0	0.13	4.1	0.13	3.9
18T>C		0.59	1.8	0.55	1.9	0.59	1.8	0.76	1.4
20A>G	Hb Lavagna	0.59	**4.4	0.37	**7.0	0.29	***9.1	0.28	***9.5
20A>T	HbS	0.14	****34.4	0.13	****37.6	0.12	****40.4	0.09	****50.7
20A>C	Hb G-Makassar	0.17	3.1	0.13	4.1	0.12	4.4	0.12	4.5
21G>C		0.21	2.5	0.27	1.9	0.22	2.4	0.18	3.0
22G>C	Hb Bellevue III	0.21	0	0.27	0.0	0.28	0.0	0.20	0.0

* $p = 0.0082$
** $p < 0.0060$
*** $p < 3x10^{-4}$
**** $p < 10^{-10}$

Table S4: Fold change of observed *de novo* rates from the genome-wide average (GWA) rates for point mutations in the African *HBB* ROI. Names of clinically known variants were taken from the Globin Gene Server database (Hardison et al., 2002; Hardison and Miller, 2002). GWA rates were calculated as described in section 10 based on a subset of *de novo* mutations with phasing information taken from genome-wide family sequencing studies (Rahbari et al., 2016) as well as based on relative frequencies of Extremely Rare Variants (ERVs) for the 3-mer, 5-mer and 7-mer genetic contexts (Carlson et al., 2018). The significance of the deviations of the mutation-specific origination rates observed here from the GWA rates is not affected by taking into account the local genetic context in three out of four cases, in accord with the fact that adjustments to the GWA rates based on context are minor compared to the variation in mutation-specific origination rates observed here. Out of 12 point mutation types studied here, the mutation-specific origination rate of the HbS mutation deviates by far the most from its GWA rate, even when taking into account the local genetic context. This effect is only strengthened in the larger contexts, as the origination rate of the HbS mutation deviates by $\sim 35\times$ from its GWA calculated based on *de novo* mutation studies and by $\sim 38\times$, $\sim 40\times$ and $\sim 51\times$ from its GWA calculated based on ERVs for the 3-mer, 5-mer and 7-mer contexts, respectively.
