# Supplementary material for: De novo mutation rates at the single-mutation resolution in a human HBB gene region associated with adaptation and genetic disease
Source: Genome Res. 2022 Mar;32(3):488–98. doi: 10.1101/gr.276103.121 (PMC8896469; doi:10.1101/gr.276103.121)
Supplement: Supplemental Material [file supp_gr.276103.121_SUPPLEMENTAL_v1.1.zip › documents/Parameter_file_preparation.pdf]

Introduction

The MEMDS analysis pipeline is a software package accompanying the MEMDS sequencing protocol. The pipeline analyzes deep-sequencing data produced by MEMDS and outputs summary tables of mutations found in the analyzed data relative to the reference gene(s).

Preparation of the parameter files

The pipeline relies on user-defined data in the parameter files during its run. All the files are under the "scripts" directory. **Before starting a new run of the pipeline, always remember to check that the parameters are correct and that they match your data!**

The pipeline utilizes the following parameter files during its run:

more_scripts/samples_table_0.txt:

This file defines parameters for merging partial ".fastq" files into a single data file. The file contains three columns:

1) **pair**: a serial number assigned by the user to the analyzed fastq files. For **single-end** data, **each read file** should have a unique pair number. For **paired-end** data, **each pair of files** should have a unique pair number. Forward and reverse read files belonging to the same pair should share the same pair number.

2) **sample:** A name of the sample to which the read files belong. All files sharing the same sample name would be merged, resulting in a single ".fastq" file per sample for single-end data, and a pair of files for paired-end data (forward and reverse reads).

3) **file:** A full path to the location where the read files are stored, ending with the name of the file:

e.g.: /data/home/user/experiment/Raw_data/S1_L001_R1_001.fastq.gz.

For **paired-end** data the script is designed to parse a **first** path in the pair as containing a **forward-read** file, and the second path as containing a **reverse-read** file. **Before concatenating the files, always ensure that they are listed in the correct order across all analyzed pairs!**

scripts/wildcard_adapters_1.fa:

This file contains sequence information of adapters and other contaminants that might be present in the analyzed data. Sequences specified in this file would be removed from the raw sequence data during the quality control step.

The adapter sequence file can be opened with any text editor program to check its contents and add additional sequences to it, as needed. New sequences should be added in FASTA format, as follows:

> Sequence_name

Nucleotide Sequence

design/samples_table.txt:

This file stores information regarding the location of the analyzed data files. **It has the same structure as the "samples_table_0.txt" parameter file used for merging partial ".fastq" files (see above)**.

Note: If the data files underwent merging before the analysis, remember to provide here the location of the merged files, not the original files!

design/params_1.sh:

This file defines Conda activation settings, stores paths to additional files needed for data analysis, and some analysis parameters.

The file contains the following parameter information:

1) **Conda settings:**

. /path_to_conda_install_dir/miniconda2/etc/profile.d/conda.sh

conda activate modules3

The first line invokes the "conda.sh" script, so that Conda commands can be used from the shell. This line should contain **a full path** to the "conda.sh" script (found within the Conda installation directory under "etc/profile.d/"). **Important:** The dot before the path is a part of the command, not a typo!

The second line activates the Conda environment. If the pipeline-related environment was created with a custom name (see explanation on Conda above), the default

“modules3” name in the command should be replaced by the custom name.

In case that the dependencies were installed manually and the installation directories were added to the $PATH, this part of the script can be removed. Alternatively, it can be used to export the location of the manually installed programs to the environment, instead of the Conda commands. To export the paths, use the following command:
export PATH=$PATH:/path/to/program/:/path/to/program2/

2) **params_adapters_1:** Specifies the path to the file containing the sequence of the adapters and other contaminants that might be present in the analyzed data. This file is used to identify unwanted sequences in the data during the quality control step. By default, **params_adapters_1** points to the adapter file distributed with the pipeline - “wildcard_adapters_1.fa” (see explanation above).

3) **params_dir_out_1:** Specifies the location of the output directory, to store the results produced by the pipeline.

4) **params_dir_reference:** Specifies the location of the reference file directory. The pipeline aligns analyzed data against the reference files to identify mutations. See the “Reference file preparation” section below for further information on the reference files.

5) **params_ninimum_fastq_size_1:** Defines the minimal length threshold for analyzed sequences to be included in the mutation presence analysis. Reads shorter than the threshold will be ignored.

6) **is_SE:** Defines whether the analyzed data is paired-end or single-end. Specify ‘0’ for **paired-end** data, and ‘1’ for **single-end**.

7) **TSS:** Defines the location of the Translation Start Site (TSS) on the reference sequence. The pipeline outputs position of the mutations found in the analyzed reads relative to the TSS and relative to the first position of the reference read. Mutations found before the TSS position are not reported.

design/factors_table.txt:

This file defines parameters for barcode trimming, sorting by origin, and mutation search for analyzed sequences. It is a tab-delimited file with 17 columns:

1) **sample**: Name of the analyzed sample to which parameters appearing in the next columns would be applied. Sample names listed here should be consistent with the names listed in the 'sample' field of the 'samples_table.txt' parameter file.

2) **reference_size:** Length of the reference sequences to which the analyzed reads are aligned (in bp). **The pipeline is designed to work with multiple references having the same length, hence this field accepts only a single length value for all reference sequences used.**

3) **size_f:** Size of the sequence elements that are found at the 5' edge of the forward reads that were generated by the MEMDS procedure. This sequence includes, from 5' to 3' of the forward read, a primary barcode sequence consisting of randomized nucleotides that are unique to each target molecule, followed by bases of identifier sequence (ID-1) that are common to all reads that originate from a single sample.

4) **size_r:** Size of the sequence elements located at the 3' edge of reverse reads that were generated by the MEMDS procedure. This sequence includes, from 5' to 3' of the reverse read, a secondary barcode sequence consisting of randomized nucleotides that are unique to each target molecule, followed by bases of identifier sequence (ID-2) that are common to all reads that originate from a single sample.

5) **limit_starts:** Start position of the window in which the pipeline would search for mutations in the analyzed reads. If left empty, the pipeline would search from the first position of the reference. To specify multiple search windows, use comma-separated list of values (e.g: 15,31). Positions of identifying mutations that indicate problems with barcode attachment (see explanation below for columns 7 - 9) should also be listed here.

6) **limit_ends:** End position of the window in which the pipeline would search for mutations in the analyzed reads. If left empty, the pipeline would search until the end

of the reference. To specify multiple search windows, use comma-separated list of values (e.g: 15,84). The order of the end position values in the list should match the order of the start positions in the previous column. Positions of any identifying mutations that indicate problems with barcode attachment should also be listed here.

7) **seq_pos:** During the MEMDS procedure, each analyzed DNA sequence is barcoded with a set of unique barcodes at its 5' and 3' ends. To account for the rare events when oligonucleotides used to attach the primary barcode (5') undergo extension themselves, using the barcoded DNA as a template, a single base insertion is planted in the oligo sequence. The 'seq_pos' field specifies the position of this insertion, which allows the pipeline to identify these sequences and remove them from further analyses.

8) **seq_mut:** This field specifies what allele should be found at the position specified by the 'seq_pos' field, to mark the sequence as an extended oligo and to remove it from the analysis. The allele is listed as <reference_nucleotide><query_nucleotide>, with INDELs marked by hyphen ("-") (e.g.: -G).

9) **seq_action:** This field specifies which action to take on sequences containing the alleles defined by the previous fields. **Currently, the pipeline is designed to remove such alleles, and accepts only 'exclude' as a keyword in this field.**

10) **read_seq:** The primary and the secondary barcodes attached to the analyzed DNA contain a sequence of four nucleotides that serve as a sample identifier. The 'read_seq' field lists these identifier sequences, to allow the pipeline distinguish between sample sequences and contaminants from other libraries. A comma is used to separate between primary and secondary barcode identifiers. A vertical bar ("|") is used to separate variants of the sequence in the same barcode. For the primary barcode sequence it is advisable to include also the first three or four nucleotides of the analyzed gene following the identifier, to ensure that the barcode was attached to the right DNA sequence (e.g. - ACGTTGT|ACGTAGT,CGTG).

11) **read_pos:** This field specifies the start position of the identifier sequences listed in the 'read_seq' field, so that the pipeline knows where in the read it should look for

the identifiers. As in the previous field, a comma separates primary and secondary barcode positions, and a vertical bar separates start positions of the variants in the same barcode. The secondary barcode identifier start position is determined **from the end** of the read, therefore it is expressed as a negative value (e.g. - 15|15,-6).

12) **read_action:** This field specifies which action to take if identifier sequences were found at the right positions within the analyzed reads. **The pipeline accepts the 'include' keyword to indicate that the reads should be included in further analyses, and any other string - to remove them**. The keywords should be listed as a comma separated list, with separate values for primary and secondary barcode identifiers (e.g.: include,include).

13) **sort_pos:** This column specifies the positions in the read that can be used to sort reads by their origin gene. The positions are listed as a comma separated list, with semicolons separating identifying positions of different origin genes (e.g. - 63,64,65,66,67,68;63,64,65,66,67,68). Identifying positions of different haplotypes belonging to the same gene should be separated by an ampersand ("&").

14) **sort_nucl:** This column specifies which nucleotides should be found at the positions listed in the 'sort_pos' field to consider analyzed read as originating from a specific gene. As in the 'sort_pos' field, identifying nucleotides should be in the form of a comma separated list with semicolons separating data for different genes and ampersand separating haplotypes (e.g. - C,G,T,T,A,C;T,G,T,C,A,A). The order in which the identifying nucleotides are listed should match the order of the identifying positions listed in the previous field.

15) **sort_refs:** This column specifies the names of the genes from which the reads originate. These genes serve as a reference against which the reads of matching origin are aligned for mutation search. The names of the different genes should be separated by semicolons (e.g. - HBB;HBD). The order in which the gene names appear should match the order in which the identifying nucleotide lists appear in the previous field.

16) **sort_ref:** This column specifies the name of a default reference against which all reads whose origin could not be determined are aligned. This column should contain

only a single gene name (e.g. - HBB). It can be one of the genes listed in the previous field or some other gene.

17) **sort_match:** This column specifies what fraction of nucleotides found at the positions specified by the 'sort_pos' field should match nucleotides listed in the 'sort_nucl' field, for the read to be considered as originating from a specific gene.

Preparation of the reference files:

In order to find mutations, the pipeline compares analyzed reads against a set of reference genes defined by the user. To prepare the reference data for use by the pipeline:

1) Place **all reference sequence files** in a **directory** specified by the 'params_dir_reference' parameter in the 'params_1.sh' file. The pipeline is not designed to search for reference files at any other location.

2) Match the names of the reference files to the names appearing in the "sort_refs" and "sort_ref" fields of the "factors_table.txt" file.

3) Make sure that all reference files have **".fa" extension**. The pipeline does not recognize reference files with other extensions. Remember to check that the OS is not configured to hide file extensions by default, and that ".fa" is the actual extension of the file!

4) Check that each reference file contains only **a single reference sequence** in FASTA format. The first line should include a sequence name, starting with the ">" symbol (E.g.: >PPIA). The second line should include the sequence itself.

Example: If the "sort_refs" field contains the values "HBB;HBD" and the "sort_ref" field contains an "HBB" value, then the reference sequence directory should contain **two files**: "HBB.fa" and "HBD.fa". Each file should contain **a single reference sequence** of the appropriate gene (or part of it).
