# Supplementary material for: De novo mutation rates at the single-mutation resolution in a human HBB gene region associated with adaptation and genetic disease
Source: Genome Res. 2022 Mar;32(3):488–98. doi: 10.1101/gr.276103.121 (PMC8896469; doi:10.1101/gr.276103.121)
Supplement: Supplemental Material [file supp_gr.276103.121_SUPPLEMENTAL_v1.1.zip › documents/Pipeline_guide.pdf]

Introduction

The MEMDS analysis pipeline is a software package accompanying the MEMDS sequencing protocol. The pipeline analyzes deep-sequencing data produced by MEMDS and outputs summary tables of mutations found in the analyzed data relative to the reference gene(s).

Run requirements:

- **OS:** The pipeline can run on any system with a configured bash shell and installed Python 2.7.

- **Distributed computing:** The scripts were designed to run on Haifa University cluster managed by the SLURM Workload Manager. If run on a different cluster system - job submission parameters should be adjusted accordingly.

To run the pipeline on a local machine, job submission scripts can be converted to a standalone version by substituting SLURM job submission command (“sbatch”) into a direct call to the job script (see examples below):

Example #1: calling to a python script with variables

In the submission script substitute “sbatch” submission code with a call to a python script “python script.py <script parameters>”

*****

Cluster submission:

```
sbatch --output="$f1.trimmed.out" --error="$f1.trimmed.err" -N1 -n1 --ntasks-per-node=1 \
-p hive1d,hive7d\
--wrap "python trim7.py \"$f1\" \"$f1.trimmed\" \"$size_f1\" \"$size_r1\" \"$read_seq\" \"$read_pos\" \"$read_action\""
```

Local run:

```
python trim7.py \"$f1\" \"$f1.trimmed\" \"$size_f1\" \"$size_r1\" \"$read_seq\" \"$read_pos\" \"$read_action\"
```

*****

Example #2: calling to a bash script using environmental variables

In the submission script substitute “sbatch” submission code with a call to a bash script “bash script.sh <script parameters>”. If the script requires external parameters to run, they need to be exported to the environment prior to the work script invocation using the “export” command.

*****

Cluster submission:

```
sbatch -N1 -n20 --ntasks-per-node=20 \
-p hive1d,hive7d \
-o "$fout1".out -e "$fout1".err \
    --export=ref1="$ref1",f1="$f1",fout1="$fout1",threads=20,ref1_length="$ref1_length",params_1="$params_1" \
bwa_job9.sh
```

Local run:

```
export ref1="$ref1" f1="$f1" fout1="$fout1" threads=20 \
ref1_length="$ref1_length" params_1="$params_1"
bash bwa_job9.sh
```

*****

- **External dependencies:** The easiest way to install and manage all the external programs used by the pipeline is with a Conda package manager (see below). Alternatively, the following programs and libraries need to be installed prior to running the pipeline:

- biopython
- bwa
- cutadapt
- fastqc
- pear
- perl
- picard
- pysam
- samtools
- seqtk

- trimmomatic

Note: The pipeline uses a direct call to invoke the required programs. Therefore, in case of manual installation, all program installation folders need to be added to the $PATH for the pipeline to execute them correctly.

Installation of dependencies with Conda:

1) Install Miniconda (**https://docs.conda.io/en/latest/miniconda.html**).
2) Install Bioconda channel (**https://bioconda.github.io**).
3) Use Conda to create an environment with the required dependencies:
-----
conda create -n modules3 python=2.7 bwa cutadapt fastqc pear perl picard pysam biopython samtools seqtk trimmomatic
-----
Note: The "-n" option defines the name of the new environment created by Conda. The MEMDS analysis pipeline uses the name "modules3", but it can be changed to any name.
4) Check that all the dependencies were successfully installed.

a) **Run:** *conda env list*. This will show you a list of existing Conda environments. Check that the newly created environment is on the list.

b) **Run:** *conda list -n modules3 > list_env.txt.* This will create a list of all linked packages in the Conda environment specified by the "-n" option, and output them to the **"list_env.txt"** file. Check the file to see that all programs listed in step 3 appear in the environment.

Note: If the environment was created with a custom name, use your environment name after the "-n" option, rather than the default name "modules3".

Preparation of the parameter files

The pipeline relies on user-defined data in the parameter files during its run. All the files are under the "scripts" directory. **Before starting a new run of the pipeline, always remember to check that the parameters are correct and that they match your data!**
The pipeline utilizes the following parameter files during its run:

more_scripts/samples_table_0.txt:

This file defines parameters for merging partial ".fastq" files into a single data file. The file contains three columns:

1) **pair**: a serial number assigned by the user to the analyzed fastq files. For **single-end** data, **each read file** should have a unique pair number. For **paired-end** data, **each pair of files** should have a unique pair number. Forward and reverse read files belonging to the same pair should share the same pair number.

2) **sample:** A name of the sample to which the read files belong. All files sharing the same sample name would be merged, resulting in a single ".fastq" file per sample for single-end data, and a pair of files for paired-end data (forward and reverse reads).

3) **file:** A full path to the location where the read files are stored, ending with the name of the file:
e.g.: /data/home/user/experiment/Raw_data/S1_L001_R1_001.fastq.gz.
For **paired-end** data the script is designed to parse a **first** path in the pair as containing a **forward-read** file, and the second path as containing a **reverse-read** file. **Before concatenating the files, always ensure that they are listed in the correct order across all analyzed pairs!**

scripts/wildcard_adapters_1.fa:

This file contains sequence information of adapters and other contaminants that might be present in the analyzed data. Sequences specified in this file would be removed from the raw sequence data during the quality control step.
The adapter sequence file can be opened with any text editor program to check its contents and add additional sequences to it, as needed. New sequences should be added in FASTA format, as follows:
> Sequence_name
Nucleotide Sequence

design/samples_table.txt:

This file stores information regarding the location of the analyzed data files. **It has**

the same structure as the "samples_table_0.txt" parameter file used for merging partial ".fastq" files (see above).

Note: If the data files underwent merging before the analysis, remember to provide here the location of the merged files, not the original files!

design/params_1.sh:

This file defines Conda activation settings, stores paths to additional files needed for data analysis, and some analysis parameters.

The file contains the following parameter information:

1) **Conda settings:**

. /path_to_conda_install_dir/miniconda2/etc/profile.d/conda.sh
conda activate modules3

The first line invokes the "conda.sh" script, so that Conda commands can be used from the shell. This line should contain **a full path** to the "conda.sh" script (found within the Conda installation directory under "etc/profile.d/"). **Important:** The dot before the path is a part of the command, not a typo!
The second line activates the Conda environment. If the pipeline-related environment was created with a custom name (see explanation on Conda above), the default "modules3" name in the command should be replaced by the custom name.

In case that the dependencies were installed manually and the installation directories were added to the $PATH, this part of the script can be removed. Alternatively, it can be used to export the location of the manually installed programs to the environment, instead of the Conda commands. To export the paths, use the following command:
export PATH=$PATH:/path/to/program/:/path/to/program2/

2) **params_adapters_1:** Specifies the path to the file containing the sequence of the adapters and other contaminants that might be present in the analyzed data. This file is used to identify unwanted sequences in the data during the quality control step. By default, **params_adapters_1** points to the adapter file distributed with the pipeline - "wildcard_adapters_1.fa" (see explanation above).

3) **params_dir_out_1:** Specifies the location of the output directory, to store the results produced by the pipeline.

4) **params_dir_reference:** Specifies the location of the reference file directory. The pipeline aligns analyzed data against the reference files to identify mutations. See the “Reference file preparation” section below for further information on the reference files.

5) **params_ninimum_fastq_size_1:** Defines the minimal length threshold for analyzed sequences to be included in the mutation presence analysis. Reads shorter than the threshold will be ignored.

6) **is_SE:** Defines whether the analyzed data is paired-end or single-end. Specify ‘0’ for **paired-end** data, and ‘1’ for **single-end**.

7) **TSS:** Defines the location of the Translation Start Site (TSS) on the reference sequence. The pipeline outputs position of the mutations found in the analyzed reads relative to the TSS and relative to the first position of the reference read. Mutations found before the TSS position are not reported.

design/factors_table.txt:

This file defines parameters for barcode trimming, sorting by origin, and mutation search for analyzed sequences. It is a tab-delimited file with 17 columns:

1) **sample**: Name of the analyzed sample to which parameters appearing in the next columns would be applied. Sample names listed here should be consistent with the names listed in the ‘sample’ field of the ‘samples_table.txt’ parameter file.

2) **reference_size:** Length of the reference sequences to which the analyzed reads are aligned (in bp). **The pipeline is designed to work with multiple references having the same length, hence this field accepts only a single length value for all reference sequences used.**

3) **size_f:** Size of the sequence elements that are found at the 5’ edge of the forward reads that were generated by the MEMDS procedure. This sequence includes, from 5’ to 3’ of the forward read, a primary barcode sequence consisting of randomized nucleotides that are unique to each target molecule, followed by bases of identifier sequence (ID-1) that are common to all reads that originate from a single sample.

4) **size_r:** Size of the sequence elements located at the 3’ edge of reverse reads that were generated by the MEMDS procedure. This sequence includes, from 5’ to 3’ of the reverse read, a secondary barcode sequence consisting of randomized nucleotides that are unique to each target molecule, followed by bases of identifier sequence (ID-2) that are common to all reads that originate from a single sample.

5) **limit_starts:** Start position of the window in which the pipeline would search for mutations in the analyzed reads. If left empty, the pipeline would search from the first position of the reference. To specify multiple search windows, use comma-separated list of values (e.g: 15,31). Positions of identifying mutations that indicate problems with barcode attachment (see explanation below for columns 7 - 9) should also be listed here.

6) **limit_ends:** End position of the window in which the pipeline would search for mutations in the analyzed reads. If left empty, the pipeline would search until the end of the reference. To specify multiple search windows, use comma-separated list of values (e.g: 15,84). The order of the end position values in the list should match the order of the start positions in the previous column. Positions of any identifying mutations that indicate problems with barcode attachment should also be listed here.

7) **seq_pos:** During the MEMDS procedure, each analyzed DNA sequence is barcoded with a set of unique barcodes at its 5’ and 3’ ends. To account for the rare events when oligonucleotides used to attach the primary barcode (5’) undergo extension themselves, using the barcoded DNA as a template, a single base insertion is planted in the oligo sequence. The ‘seq_pos’ field specifies the position of this insertion, which allows the pipeline to identify these sequences and remove them from further analyses.

8) **seq_mut:** This field specifies what allele should be found at the position specified by the 'seq_pos' field, to mark the sequence as an extended oligo and to remove it from the analysis. The allele is listed as <reference_nucleotide><query_nucleotide>, with INDELs marked by hyphen ("-") (e.g.: -G).

9) **seq_action:** This field specifies which action to take on sequences containing the alleles defined by the previous fields. **Currently, the pipeline is designed to remove such alleles, and accepts only 'exclude' as a keyword in this field.**

10) **read_seq:** The primary and the secondary barcodes attached to the analyzed DNA contain a sequence of four nucleotides that serve as a sample identifier. The 'read_seq' field lists these identifier sequences, to allow the pipeline distinguish between sample sequences and contaminants from other libraries. A comma is used to separate between primary and secondary barcode identifiers. A vertical bar ("|") is used to separate variants of the sequence in the same barcode. For the primary barcode sequence it is advisable to include also the first three or four nucleotides of the analyzed gene following the identifier, to ensure that the barcode was attached to the right DNA sequence (e.g. - ACGTTGT|ACGTAGT,CGTG).

11) **read_pos:** This field specifies the start position of the identifier sequences listed in the 'read_seq' field, so that the pipeline knows where in the read it should look for the identifiers. As in the previous field, a comma separates primary and secondary barcode positions, and a vertical bar separates start positions of the variants in the same barcode. The secondary barcode identifier start position is determined **from the end** of the read, therefore it is expressed as a negative value (e.g. - 15|15,-6).

12) **read_action:** This field specifies which action to take if identifier sequences were found at the right positions within the analyzed reads. **The pipeline accepts the 'include' keyword to indicate that the reads should be included in further analyses, and any other string - to remove them**. The keywords should be listed as a comma separated list, with separate values for primary and secondary barcode identifiers (e.g.: include,include).

13) **sort_pos:** This column specifies the positions in the read that can be used to sort

reads by their origin gene. The positions are listed as a comma separated list, with semicolons separating identifying positions of different origin genes (e.g. - 63,64,65,66,67,68;63,64,65,66,67,68). Identifying positions of different haplotypes belonging to the same gene should be separated by an ampersand (“&”).

14) **sort_nucl:** This column specifies which nucleotides should be found at the positions listed in the ‘sort_pos’ field to consider analyzed read as originating from a specific gene. As in the ‘sort_pos’ field, identifying nucleotides should be in the form of a comma separated list with semicolons separating data for different genes and ampersand separating haplotypes (e.g. - C,G,T,T,A,C;T,G,T,C,A,A). The order in which the identifying nucleotides are listed should match the order of the identifying positions listed in the previous field.

15) **sort_refs:** This column specifies the names of the genes from which the reads originate. These genes serve as a reference against which the reads of matching origin are aligned for mutation search. The names of the different genes should be separated by semicolons (e.g. - HBB;HBD). The order in which the gene names appear should match the order in which the identifying nucleotide lists appear in the previous field.

16) **sort_ref:** This column specifies the name of a default reference against which all reads whose origin could not be determined are aligned. This column should contain only a single gene name (e.g. - HBB). It can be one of the genes listed in the previous field or some other gene.

17) **sort_match:** This column specifies what fraction of nucleotides found at the positions specified by the ‘sort_pos’ field should match nucleotides listed in the ‘sort_nucl’ field, for the read to be considered as originating from a specific gene.

Preparation of the reference files:

In order to find mutations, the pipeline compares analyzed reads against a set of reference genes defined by the user. To prepare the reference data for use by the pipeline:

1) Place **all reference sequence files** in a **directory** specified by the

'params_dir_reference' parameter in the 'params_1.sh' file. The pipeline is not designed to search for reference files at any other location.

2) Match the names of the reference files to the names appearing in the "sort_refs" and "sort_ref" fields of the "factors_table.txt" file.

3) Make sure that all reference files have **".fa" extension**. The pipeline does not recognize reference files with other extensions. Remember to check that the OS is not configured to hide file extensions by default, and that ".fa" is the actual extension of the file!

4) Check that each reference file contains only **a single reference sequence** in FASTA format. The first line should include a sequence name, starting with the ">" symbol (E.g.: >PPIA). The second line should include the sequence itself.

Example: If the "sort_refs" field contains the values "HBB;HBD" and the "sort_ref" field contains an "HBB" value, then the reference sequence directory should contain **two files**: "HBB.fa" and "HBD.fa". Each file should contain **a single reference sequence** of the appropriate gene (or part of it).

Quick start guide

This section provides basic information needed to run the pipeline. For a more detailed information regarding each analysis step and its associated output, refer to the next section.

Note 1: The **"$i"** symbol is used to indicate that a numerical option should be provided after the command name, with available options listed in parentheses.
For example: *bash my_script.sh $i (1-3)* means that **my_script.sh** accepts numerical options 1 to 3. To complete the step, **my_script.sh** should be run with all the options, sequentially, according to the order defined in parentheses.

Note 2: When running on a cluster, for each job submitted by the script the following message would appear: "Submitted batch job #job_serial_number".
Before moving to the next option in the script or to the next step in the pipeline,

a**lways check that all running jobs were completed successfully.** To check job status, use the following commands (for SLURM systems):
1) *squeue -u "username"| grep -c "username"* – this command displays the number of jobs in the queue for the account "username". Completed or canceled jobs would be removed from the queue.

2) *sacct -u "username"* – this command lists all the jobs that ran on the account "username" in the current session. The last column indicates for each job if it is completed, canceled, or still running. Ensure that all relevant jobs have 'Completed' status before submitting new ones!

In addition, remember to check the ".err" and ".out" log files produced during job runs on the cluster. Log files are placed in the same directory as the result files produced by the pipeline in each step or sub-step. They record warnings and error messages raised by the script or by the cluster during a job run.

Run instructions:

1) Place the directory containing the pipeline scripts and associated parameter files in the directory containing the directory with the analyzed files.

2) Navigate to the script directory from the command line (***cd /path/to/projects/scripts***) to use the pipeline. All pipeline commands should be run from within the scripts directory.

3) **(Optional) Merging raw data:**
   a) *bash concatenate_partfiles.sh*
   b) **Local run:** *bash more_scripts/samples_table_0.sh.concat.sh* **or**
   c) **Cluster run:** *srun bash more_scripts/samples_table_0.sh.concat.sh*

4) **Formatting parameter data for use by the pipeline:**
   a) **Single-end data:** *bash setting_1-SE.sh* **or**
   b) **Paired-end data:** *bash setting_1-PE.sh*

5) **Quality control and clearing of raw data + paired-end data merging**:

a) **Single-end data:** *bash filter-SE4.sh $i* (1-3) **or**

b) **Paired-end data:** *bash filter-PE4.sh $i* (1-4)

6) **Selecting reads with correct barcodes and separating between barcodes and genomic data**:

a) *bash trim7.sh 1*

7) **Sorting reads by their origin gene:**

a) *bash sort2.sh 1*

8) **Mapping reads to the reference sequences:**

a) *bash bwa9.sh $i* (1-2)

9) **Making alignment files viewable in IGV:**

a) *bash create_dummy_genome5.sh 1*

10) **Creating a mutation table that lists sequencing quality alongside each mutation:**

a) *bash sam_to_mutation-list-3.sh 1*

11) **Creating a table of mutations found in the analyzed data:**

a) *bash sam_to_mutation-table_5.0.sh 1*

12) **Detecting consensus mutations that pass a set of user-defined thresholds:**

a) *bash consensus_15.sh $i* (1-2)

Pipeline guide

This section provides detailed information regarding the various steps of the MEMDS analysis pipeline. The scripts making up the pipeline are meant to be run in the sequential order listed below, unless noted otherwise. Optional steps are applicable only to certain types of data, and can be skipped if not needed.

Before the run:

1) The pipeline should be run from the location containing the analyzed data files.

Create a copy of the pipeline 'scripts' directory inside the directory that contains the directory with the analyzed ".fastq" files.

2) Enter the 'scripts' directory you have created in the previous step. Pipeline scripts should be invoked from inside the "scripts" directory to run properly.

3) Refer to the **"Preparation of the parameter files"** section above to ensure that all pipeline parameter files contain run-relevant information and are found at the proper directories.

Running the pipeline:

1) (Optional) Merging raw data:

Step description:

This step merges data from multiple ".fastq" files into a single file. It is used in cases where sequencing of the analyzed data was done on multiple lanes.

Run instructions:

a) **Run script:** *bash concatenate_partfiles.sh*

b) This command will create three files in the "scripts/more_scripts" folder:

1) samples_table_0.sh
2) samples_table_0.sh.concat.sh
3) samples_table_0.sh.concatenated.sh

These files would provide information needed to merge the input data.

c) **Run script:**

Cluster: *srun bash more_scripts/samples_table_0.sh.concat.sh*

Local: *bash more_scripts/samples_table_0.sh.concat.sh*

d) Inspect the resulting merged ".fastq" files.

Output description:

In this step, two output files are important: "samples_table_0.sh.concat.sh" and "samples_table_0.sh.concatenated.sh".

a) **samples_table_0.sh.concat.sh** - this bash script contains information and commands needed to concatenate partial ".fastq" files together. Before running this

script, check that it does not contain any error messages and that the paths to the input files listed there are correct. Also note the names and the output location of the concatenated files.

b) **samples_table_0.sh.concatenated.sh** - this script contains the names and the output directory of the concatenated ".fastq" files. It is used mainly for diagnostic purposes, to ensure that the output names and output location are correct.

If the **"samples_table_0.sh.concat.sh"** script contains error messages or points to the wrong input files, check that the "samples_table_0.txt" file is properly formatted and contains the correct paths to the partial files. For a detailed explanation on this file, see the **"Preparation of the parameter files"** section. All changes should be done through the parameter file, and not directly in the **"samples_table_0.sh.concat.sh"** script!

2) **Formatting parameter data:**

Step description:

This step organizes the parameter data from "samples_table.txt" and "factors_table.txt" found in the "scripts/design" folder, into a single bash file named "samples_table.sh". The subsequent pipeline scripts read parameters needed for the analysis from this file.

Run instructions:

a) Check that all the parameter files found in the "scripts/design" folder include correct parameter data. For a detailed explanation on preparation of these files, see the **"Preparation of the parameter files"** section.

b) **Run the script matching the input data type:**

1) **Single-end data:** *bash setting_1-SE.sh* **or**
2) **Paired-end data:** *bash setting_1-PE.sh*

c) Inspect the output "samples_table.sh" file for error messages or incorrect parameter values.

Output description:

The "samples_table.sh" bash file contains information from the "samples_table.txt"

and “factors_table.txt” parameter files. It is located in the “scripts/design” folder, same as the text parameter files. The data in the file is organized by sample, all parameters following the “title” field which lists name of the sample relate to this sample. Each parameter is listed in a separate line. The names of the parameters match column names in the “factors_table.txt” file.

If the **“samples_table.sh”** file contains error messages or wrong parameters, check that the “samples_table.txt” and “factors_table.txt” files are properly formatted and contain the correct data. For a detailed explanation on preparation of these files, see the **“Preparation of the parameter files”** section. All changes to the parameters should be done through these text parameter files and not through the resulting bash file!

---

3) Quality control and cleaning of the raw data:

Step description:

This step is a quality control step. It checks that the raw input data is of sufficient quality and removes from it contaminants that might interfere with the quality of the subsequent data analyses.

Note: This step uses predefined parameters to trim and clean raw read data using the Cutadapt and Trimmomatic programs. These parameters can be adjusted by supplying manual parameters to the relevant program(s) in the “fastq-filter_job_3.sh” script.
Alternatively, users can perform their own cleaning of the raw data and provide the resulting files as input to the next pipeline steps. In this case, several rules need to be observed:

1) The files should be supplied in the “.fastq” format.
2) In the case of the paired-end data, forward and reverse reads need to be merged, either before or after the trimming and cleaning steps. The pipeline expects to find a single read-containing file per analyzed sample at the end of the quality control step.
3) Manually created files should be placed in the pipeline output directory (as defined by the user in the “params_1.sh” file) under the **“filtered”** directory. Their names should be as follows: **<sample_name>_idx$i.assembled.filtered.fastq** (paired-end data) or **<sample_name>_idx$i.filtered.fastq** (single-end data). The sample name and its accompanying index are defined by the “title” rows in the “samples_table.sh”

file, for example:

* title[0]="contLL087" -> contLL087_idx0.assembled.filtered.fastq;

* title[1]="expLL087" -> expLL087_idx1.assembled.filtered.fastq;

Step 3-1: Data quality check

Run instructions:

a) **Run script matching input data type:**

1) **Single-end data:** *bash filter-SE4.sh 1* **or**

2) **Paired-end data:** *bash filter-PE4.sh 1*

b) Wait for the job to end. Use the “sacct” and the “squeue” commands on the cluster to check job status.

c) Inspect the output files in the output folder under the “fastqc” directory.

Output description:

This sub-step uses FastQC program to analyze the sequencing quality of the input files. The output files are stored under the “fastqc” directory in your output directory (as defined in “params_1.sh” file). In case of the paired-end data, forward and reverse read files are processed separately.

This sub-step creates the following files:

1) **<sample_name>.err** and **<sample_name>.out** - log files produced during the run of the job on the cluster. Check that the “.err” file does not contain any error messages and that the “.out” file contains the following line: “Analysis complete for *sample_name.fastq*”.

2) **<sample_name>_fastqc.html** - a graphical summary of the FastQC program results. It can be opened by any web browser. FastQC provides assessment of sequencing quality of the input fastq files. For more information on the FastQC output, refer to the https://www.bioinformatics.babraham.ac.uk/projects/fastqc/.

3) **<sample_name>_fastqc.zip** - an archive containing raw summary files produced by FastQC from which the program creates graphical output summary.

Step 3-2: Paired-end data merging

Run instructions:

a) **Run script:** *bash filter-PE4.sh 2*

b) Wait for the job to end. Use the "sacct" and the "squeue" commands on the cluster to check job status.

c) Inspect the output files in the output folder under the "filtered" directory.

Output description:

This sub-step uses Pear to merge forward and reverse reads of paired-end data. The output files are stored under the "filtered" directory in your output directory (as defined in the "params_1.sh" file).

This sub-step creates the following files:

1) **<sample_name>.assembled.err** - error log file produced during the run of the job on the cluster. Check that the ".err" file does not contain error messages, to ensure that the job was completed successfully.

2) **<sample_name>.assembled.out, <sample_name>.assembled.info** - these files contain job summary log produced by Pear at the end of the run. Both files are identical. The ".info" file is created in addition to the ".out" file, to log Pear output, because the latter is not created during stanalone script run.
Check either log file to ensure that Pear processed all the reads. Check assembly statistics at the end of the file on the percentage of assembled reads. Reads that remain unmerged will be removed from further analyses.

3) **<sample_name>.discarded.fastq, <sample_name>.unassembled.forward.fastq, <sample_name>.unassembled.reverse.fastq** - these files contain reads that Pear failed to merge, or reads that were discarded because they did not pass the quality thresholds. Reads contained in these files are not processed further by the pipeline.

4) **<sample_name>.assembled.fastq -** this file contains merged reads that are used by the pipeline in the subsequent analyses. A separate file is created for each analyzed sample.

<u>Step 3-3: Read trimming</u>

Run instructions:

a) **Run script matching input data type:**

1) **Single-end data:** *bash filter-SE4.sh 2* **or**

2) **Paired-end data:** *bash filter-PE4.sh 3*

b) Wait for the job to end. Use the "sacct" and the "squeue" commands on the cluster to check job status.

c) Inspect the output files in the output folder under the "filtered" directory.

Output description:

This sub-step uses the Cutadapt and Trimmomatic programs to trim the analyzed reads. The aim is to remove low quality bases and contaminants, such as adapter sequences, from the analyzed reads. Trimmed reads are considered of high quality and ready for further analyses. The output files are stored under the "filtered" directory in the output directory (as defined in the "params_1.sh" file). In case of the paired-end data, this step processes merged read data generated by Pear in the previous step.

This sub-step creates the following files:

1) **<sample_name>.assemb.filtered.err** - a log file produced during the run of the job on the cluster, containing a summary of the Trimmomatic run. Check that the log does not contain error messages and indicates that the Trimmomatic run was completed successfully. Check statistics of removed sequences - if a large portion of the sequences do not pass the Trimmomatic filters, it might indicate problems with the quality of the input data.

2) **<sample_name>.assemb.filtered.out** - a log file produced during the run of the job on the cluster. It contains a summary of the Cutadapt run. Refer here for detailed explanation on the Cutadapt run summary log: https://cutadapt.readthedocs.io/en/stable/guide.html#how-to-read-the-report.

3) **<sample_name>.assembled.filtered.fastq** - a ".fastq" file containing all reads surviving the trimming and cleaning. These reads are considered high-quality and constitute the input for the next pipeline steps.

Step 3-4: Trimmed data quality check

Run instructions:

a) **Run script matching input data type:**

1) **Single-end data:** *bash filter-SE4.sh 3* **or**
2) **Paired-end data:** *bash filter-PE4.sh 4*

b) Wait for the job to end. Use the "sacct" and the "squeue" commands on the cluster to check job status.

c) Inspect the output files in the output folder under the "fastqc" directory.

Output description:

This sub-step uses the FastQC program to analyze the quality of the merged and trimmed ".fastq" files. The aim is to check that the trimming improved the quality of the reads and removed contaminants from the final data files. The output files are stored under the "fastqc" directory in your output directory (as defined in the "params_1.sh" file).

This sub-step creates the following files:

1) **<sample_name>.assembled.fastqc.out** – a log file produced during the run of the job on the cluster. Check that the file does not contain error messages, and contains the following line: "Analysis complete for *sample_name.assembled.filtered.fastq*".

2) **<sample_name>.assembled.filtered_fastqc.html** - a graphical summary of the FastQC program results. For more information on the FastQC output, refer to https://www.bioinformatics.babraham.ac.uk/projects/fastqc/.

3) **<sample_name>.assembled.filtered_fastqc.zip** - an archive containing raw summary files produced by FastQC from which the program creates the graphical output summary.

Step 3-5: (Optional) Sub-sampling ".fastq" files

Run instructions:

a) **Run script matching input data type:**

1) **Single-end data:** *bash filter-SE4.sh 4* **or**
2) **Paired-end data:** *bash filter-PE4.sh 5*

b) Wait for the job to end. Use the "sacct" and the "squeue" commands on the cluster

to check job status.

c) Inspect the output files in the output folder under the "tests" directory.

Output description:

This sub-step creates a sub-sample of the analyzed ".fastq" files with 10,000 randomly chosen reads. It sub-samples both the raw data files and the ".fastq" files after the quality control steps (merging, trimming). Due to their small size, these sub-samples can be inspected manually and the results of this inspection can be compared to the pipeline output, to ensure that the calculations done by the pipeline do not contain any unexpected errors. The output files are stored under the "tests" directory in the output directory (as defined in the "params_1.sh" file).

---

4) Barcode trimming:

Step description:

This step filters reads based on the presence of the correct sample-identifier sequences in the primary and secondary barcodes attached to the read. Additionally, it separates the genetic sequence coming from the donor and the barcode sequences attached to it, and moves the barcode sequences to the header row of the relevant read.

The script also checks the per-base sequencing quality of the barcode sequences. Positions not passing the quality threshold are marked by 'N'. The quality score is calculated using the "phred+33" score scheme. To define a different scoring scheme, or to adjust the quality threshold, modify the "barcodeQuality_phred33" function in the "trim7.py" script.

Run instructions:

a) Check that the "factors_table.txt" contains the correct values in the columns: "size_f", "size_r", "read_seq", "read_pos", and "read_action". In case of incorrect values, adjust the table and **repeat step 2** above to produce a correct bash parameter file, before starting this step.

b) **Run script:** *bash trim7.sh 1*

c) Wait for the job to end. Use the "sacct" and the "squeue" commands on the cluster to check job status.

d) Inspect the output files in the output folder under the "filtered" directory.

Output description:

This step sorts reads into two ".fastq" files - one containing reads with correct identifier sequences and one with wrong identifiers (in either the 5' or the 3' barcode). Upon step completion, the following files are added to the "filtered" directory:

1) **<sample_name>.assembled.filtered.fastq.trimmed.err,**
<sample_name>.assembled.filtered.fastq.trimmed.out - log files produced during the run of the job on the cluster. Check that the ".err" file does not contain error messages and that the ".out" file contains data from the "read_seq", "read_pos", and "read_action" columns of the "factors_table" file.

2) **<sample_name>.assembled.filtered.fastq.trimmed.fastq,**
<sample_name>.assembled.filtered.fastq.trimmed.barcodes - a ".fastq" file containing trimmed reads and its associated list of 5' and 3' barcode sequences found in these reads. Contain reads and barcode sequences (5' and 3') with correct identifiers.
The barcode list serves for diagnostic purposes. It can be used to check that the script identifies as barcodes and trims correct parts of the read. Additionally, it can be used to analyze variations of the barcode sequences to check that no barcodes appear more frequently than can be expected from the MEMDS protocol.

3) **<sample_name>.assembled.filtered.fastq.trimmed.wrongId.fastq,**
<sample_name>.assembled.filtered.fastq.trimmed.wrongId.barcodes - a ".fastq" file containing trimmed reads and its associated list of 5' and 3' barcode sequences found in these reads. Contain reads and barcode sequences (5' and 3') with identifiers that do not match the user-defined pattern and, likely, represent contaminants.
A list of barcodes with non-matching identifier sequences can be compared against identifiers of other samples to detect sample cross-contamination.

4) **<sample_name>.assembled.filtered.fastq.trimmed.log** - a summary file listing how many analyzed sequences had wrong identifiers in their barcodes. If a high number of reads with wrong identifiers is found, check that the identifier parameters listed in the "factors_table.txt" are correct. If the parameters are correct, this might

indicate an issue in the experimental procedure: cross-contamination from other samples, problems with barcode attachment, or problems with read amplification.

---

5) Trimmed read sorting:

Step description:

This step sorts reads by their origin, using conserved positions in each gene of origin as an identifier of read identity. Reads whose origin cannot be determined are put in a separate list.

Presence of each gene-identifying base is checked within the [-3, +3] window from the base location in the reference, to account for possible indels in the read. To change the size of this window, change in the script "sort2.py" the values of the following variable: *for offset in [0,-1,1,-2,2,-3,3]*.

Run instructions:

a) Check that the "factors_table.txt" file contains the correct values in the columns: "sort_pos", "sort_nucl", "sort_refs", "sort_ref", and "sort_match". In case of incorrect values, adjust the table and **repeat step 2** described above to produce a correct bash parameter file, before starting this step.

b) **Run script:** *bash sort2.sh 1*

c) Wait for the job to end. Use the "sacct" and the "squeue" commands on the cluster to check job status.

d) Inspect the output files in the output folder under the "sorted" directory.

Output description:

This script organizes reads coming from different genes in separate ".fastq" files. Upon step completion, the following files will be added to the "sorted" directory:

1) **<sample_name>.err, <sample_name>.out** - Log files produced during the run of the job on the cluster. For each sample, check that the ".err" file does not contain error messages and that the ".out" file contains the line "closing <sample_name>.<ref_name>" to indicate that all reads were analyzed and sorted.

2) **<sample_name>.<ref_name>.fastq** - fastq file containing all reads from a given sample originating in a specific gene. A separate file is created for each sample and

each reference gene against which the reads are sorted.

3) **<sample_name>.<default_ref_name>.others.fastq** - fastq file containing reads whose gene of origin could not be identified reliably. To find mutations in these reads, they will be aligned against a reference gene considered to be a "default". A "default" reference can be one of the genes used to sort the reads, or some other gene.

4) **<sample_name>.<ref_name>.withIndels.log,**
<sample_name>.<default_ref_name>.others.withIndels.log - log files listing sorted reads with shifted origin-identifying positions, indicating presence of indels upstream to them. These files can be used to analyze indel-containing reads separately from the rest. By default, they are not analyzed by the pipeline and all reads having a specific origin (both with and without indels) are put together in the relevant ".fastq" file.

---

6) Mapping reads to reference sequences:

Step description:

This step aligns the sorted and trimmed reads against their reference sequences. Reads with an undetermined origin are aligned against a default reference chosen by the user. Reads containing barcodes with low quality bases (as identified in step 4) are skipped.

Step 6-1: Reference sequence indexing

Run instructions:

a) Check that all reference files are properly formatted and present in the reference file directory, as described in the **"Preparation of the reference files"** section.

b) **Run script:** *bash bwa9.sh 1*

c) Inspect the output index files in the reference file folder.

Output description:

For each reference file, index files with the following extensions should be created: ".amb", ".ann", "bwt", ".dict", ".fai", ".pac", and ".sa". Additionally, an empty file named "<ref_name>.fa.indices.OK" should be created to indicate that the job was completed successfully.

Step 6-2: Read alignment against reference

Run instructions:

a) Check that the "factors_table.txt" file contains the correct values in the columns: "reference_size", "sort_refs", and "sort_ref". In case of incorrect values, adjust the table and **repeat step 2** described above to produce a correct bash parameter file, before starting this step. Check that all reference files are present in the reference file directory.

b) **Run script:** *bash bwa9.sh 2*

c) Wait for the job to end. Use the "sacct" and the "squeue" commands on the cluster to check job status.

d) Inspect the output files in the output folder under the "mapping" directory.

Output description:

This sub-step uses the BWA-MEM aligner for read alignment. It outputs alignment files in SAM and BAM formats. The following files are created:

1) **<sample_name>.<ref_name>.bwa.err, <sample_name>.<ref_name>.bwa.out** - log files produced during the run of the job on the cluster. Check that the ".err" file contains only summary of the BWA program run and does not contain any error messages.

2) **<sample_name>.<ref_name>.bwa.0.sam** - an alignment file of analyzed reads against the relevant reference in SAM format.

3) **<sample_name>.<ref_name>.bwa.sam** - a modified SAM alignment file. The reference name in the RNAME field is substituted by the 5' barcode sequence of a given read and tag containing 3' barcode sequence is added (XB:Z:bc3_seq) to each alignment line.

4) **<sample_name>.<ref_name>.bwa.sam.header** - a list of custom header lines created for the modified SAM file. The SN field contains 5' barcode sequences of the reads, instead of the reference name, so the header lines fit to the new RNAME column names. It is used to create a sorted BAM file from the modified alignment file.

5) **<sample_name>.<ref_name>.bwa.sorted.bam, <sample_name>.<ref_name>.bwa.sorted.bam.bai -** a sorted BAM file and its associated index created from the modified SAM file. The BAM file is sorted by the alignment position of the query to the reference.

---

7) Making alignments viewable in IGV:

Step description:

This step prepares all the necessary files in the "mapping" folder to make alignment files ready for visual inspection in IGV (Integrative Genomics Viewer). Visual inspection of the alignments is important to ensure that reads are correctly aligned to the reference.

Run instructions:

a) Check that the "factors_table.txt" file contains the correct values in the columns: "sort_refs" and "sort_ref". In case of incorrect values, adjust the table and **repeat step 2** described above to produce a correct bash parameter file, before starting this step.

b) **Run script:** *bash create_dummy_genome5.sh 1*

c) Inspect the output files in the output folder under the "mapping" directory.

Output description:

This script adds two files to the "mapping" directory:

1) **<sample_name>.<ref_name>.bwa.sam.header.barcode** - a list of all unique 5' barcode sequences belonging to the reads aligned against a given reference sequence.

2) **<sample_name>.<ref_name>.bwa.sam.header.barcode.fasta** - a FASTA file where each unique 5' barcode sequence is used as the header of the reference sequence to which the reads were aligned and is followed by the reference sequence itself. The resulting file looks as follows:

...

>5_BC_SEQ_1 Reference_name

Reference_sequence

>5_BC_SEQ_2 Reference_name

Reference_sequence

...

This creates a match between reference sequence headers and reference names in the RNAME field of the modified SAM/BAM file which is needed for IGV to visualize the reads against the correct reference sequence. Using 5' barcode sequences as read identifiers allows read grouping by families originating from the same stretch of donor DNA.

8) Listing mutations per read:

Step description:

This step checks mutation presence in each read and outputs mutated positions and their sequencing quality. Each mutation is listed in a separate row. If no mutations are found, the read is designated 'WT'.

Note:

1) The script searches for mutations in a user defined window of interest. 'WT' designation means that no mutations were found in this interval and not along the whole read.

2) The script checks the sequencing quality of the mutated positions using the "phred+33" scoring scheme. If a different score is used, the "phred" variable in the "sam_to_mutation-list-3.py" needs to be adjusted accordingly.

3) Currently, the script requires that the query and the reference align at their first position (reference start and query alignment start, as listed in the SAM file, are '0') for the alignment to be included in the analysis. Alignments with non-first-base start for either reference or query are skipped.

Run instructions:

a) Check that the "factors_table.txt" file contains the correct values in the columns: "limit_starts", "limit_ends", "sort_refs", and "sort_ref". In case of incorrect values, adjust the table and **repeat step 2** described above to produce a correct bash

parameter file, before starting this step.

b) **Run script:** *bash sam_to_mutation-list-3.sh 1*

c) Wait for the job to end. Use the "sacct" and the "squeue" commands on the cluster to check job status.

d) Inspect the output files in the output folder under the "mutations" directory.

Output description:

This step creates a list of mutations per read in the analyzed reads. It creates the following files:

1) **<sample_name>.err, <sample_name>.out** - log files produced during the run of the job on the cluster. Check that the ".err" file does not contain error messages and that the last line in the ".out" file contains the word "ok", indicating that all reads were analyzed.

2) **<sample_name>.<ref_name>.bwa.sorted.bam.mutations.log.txt** - a log file listing the total count of reads in the analyzed file, count of reads that were analyzed, and count of reads that were skipped, either because they are unmapped or because read-reference alignment does not start at the beginning of the reference and/or the read.

A large number of unmapped reads might indicate contamination or sequencing issues.

A large number of reads with alignment start issues might indicate that the reference sequence includes parts of the gene that were not analyzed.

3) **<sample_name>.<ref_name>.bwa.sorted.bam.mutations.txt** - a list of mutations found in the analyzed reads, relative to the reference. Each mutation is listed in a separate line. The list contains the following information:

1) **read_name** – the ID of the read in which a mutation was found (e.g. M00654:20:000000000-J2GB9:1:1101:12401:9036).

2) **bc5** – the sequence of the 5' barcode associated with the read in which the mutation was found.

3) **bc5_count** - the number of reads having 5' barcode listed in the 'bc5' field (read family size).

4) **bc3** - the sequence of the 3' barcode associated with the read in which the mutation

was found.

5) **quality** - the sequencing quality of the position in the read in which the mutation was found. For 'WT' reads and deletions, this field gets the value 'NA'.

6) **pos** - the position of the mutation in the reference sequence, relative to the first base of the sequence. For 'WT' reads, this field gets the value 'NA'.

7) **from** - the nucleotide found in the reference sequence at the position listed in the 'pos' field. Insertions in the read relative to the reference are marked by '-'. For 'WT' reads, this field gets the value 'NA'.

8) **to** - the nucleotide found in the query sequence (read) at the position listed in the 'pos' field. Deletions in the read relative to the reference are marked by '-'. For 'WT' reads, this field gets the value 'NA'.

9) **allele** - the full name of the mutated allele, listing the name of the SNP and its position (e.g.: 39CT). If no mutations are found, this field gets the value 'WT'.

9) Listing mutations per read family:

Step description:

This step groups analyzed reads into families based on their unique 5' barcode sequence, and examines each family for the presence of mutations. It outputs a list of mutations per each family, with each mutation listed in a separate row. Reads containing no mutations in each family are designated 'WT' and are output in a separate row.

The analysis is done on all reads together, and separately on reads with a missing 3' barcode. Lack of 3' barcode might indicate that there is some issue with these reads. Therefore it is important to check reads lacking 3' barcode separately, before continuing to analyze them with the rest of the reads.

Note:

1) The script searches for mutations in a window of interest defined by the user. 'WT' (wild type) designation means that no mutations were found only in this interval and not along the whole read.

2) The script checks the sequencing quality of the mutated positions using the "phred+33" scoring scheme. If a different score is used, the "phred" variable in the "sam_to_mutation-table_5.0.py" file needs to be adjusted accordingly.

3) Currently the script requires that the query and the reference align at their first position (reference start and query alignment start, as listed in the SAM file, are ‘0’) for the alignment to be included in the analysis. Alignments with non-first-base start for either the reference or the query are skipped.

Run instructions:

a) Check that the “factors_table.txt” file contains the correct values in the columns: “seq_pos”, “seq_mut”, “seq_action”, “limit_starts”, “limit_ends”, “sort_refs”, and “sort_ref”. In case of incorrect values, adjust the table and **repeat step 2** described above to produce a correct bash parameter file, before starting this step.

b) **Run script:** *bash sam_to_mutation-table_5.0.sh 1*

c) Wait for the job to end. Use the “sacct” and the “squeue” commands on the cluster to check job status.

d) Inspect the output files in the output folder under the “tables.BC3cutoff1” directory.

Output description:

This step outputs information regarding the mutated positions and WT reads in each read family (group of reads sharing the same 5’ barcode). It creates the following files:

1) **<sample_name>.<ref_name>.bwa.sorted.bam..err, <sample_name>.<ref_name>.bwa.sorted.bam..out** - log files produced during the run of the job on the cluster. Check that the “.err” file does not contain error messages and that the last line in the ”.out” file contains the word “ok”, indicating that all reads were analyzed. Log files for analysis of 3’ barcode missing reads include the “BC3_missing” tag before the “.err” or the “.out” extension.

2) **<sample_name>.<ref_name>.bwa.sorted.bam.mutationFrequncyPerBarcode.log.txt** - a log file listing the total number of reads in the analyzed file, the count of reads that were analyzed, and the count of reads that were skipped, either because they are unmapped or because the read and the reference do not align from their first position.

The log file for analysis of 3’ barcode missing reads includes the “tag-BC3_missing” tag after “.bam” in their file name.
A large number of unmapped reads might indicate contamination or sequencing issues. A large number of reads with alignment start issues might indicate that the reference sequence includes parts of the gene that were not analyzed.

3) **<sample_name>.<ref_name>.bwa.sorted.bam.mutationFrequncyPerBarcode.txt** - a list of mutations found in the analyzed read families, relative to the reference. Each mutation is listed in a separate line. Files listing mutations only in the 3’ barcode missing reads include the “tag-BC3_missing” tag after “.bam” in their file name.
The mutation list contains the following information:
1) **ID** - a unique 5’ barcode sequence serving as the identifier of a given read family.
2) **Mutation** - the name of the mutated allele, shown as **<mutation_position_reference><reference_nucleotide><read_nucleotide>**, e.g.: 57G-. Reads not containing mutations in the interval of interest defined by the user are considered ‘WT’. INDELS are indicated by a hyphen (‘-’).
3) **Mutation_count** - the number of reads in the 5’ barcode family containing mutation specified by the “Mutation” field.
4) **HQ_mutation_count** - the number of reads in the 5’ barcode family containing mutation specified by the “Mutation” field when the sequencing quality of the mutated position in the read is above the quality threshold (default: 28). Deletions and WT reads are considered high quality by default.
5) **Total_count** - the total number of reads in the 5’ barcode family (mutated and WT).
6) **HQ_total_count** - the total number of reads in the 5’ barcode family with high sequencing quality at the position of mutation listed in the “Mutation” field (both mutated and WT). The default quality threshold is 28 (phred+33 scheme). In case of deletions and ‘WT’, all reads in the family containing this allele are considered high quality.
7) **Mutation_freq** - the proportion of reads containing mutation at a given position out of all reads in the family, when only high-quality positions are considered. Calculated as: **HQ_mutation_count/HQ_total_count**.
8) **barcode3_IDs** - a semicolon separated list of unique 3’ barcode sequences associated with reads containing high-quality mutated position defined in the

"Mutation" field.

9) **barcode3_HQ_mut_counts** - the number of reads in each 3' barcode group listed in the "barcode3_IDs" field that contains high-quality mutated position. Deletions and WT reads are considered high quality by default.

10) **barcode3_existing** – the count of distinct 3' barcode groups associated with the high-quality mutated position. Does not include reads with the "BC3_Missing" tag.

11) **barcode3_missing** - A field indicating whether any of the reads carrying high-quality mutated position lacks a 3' barcode. Has value of '0' if no such reads exist, and '1' if one or more such reads exist.

12) **barcode3_HQ_mut_freq** - the proportion of reads in each 3' barcode group containing the mutation defined by the "Mutation" field out of all reads in the 5' barcode family, when only high-quality positions are considered. Calculated as: **barcode3_HQ_mut_counts/HQ_total_count**.

13) **barcode3_all** - A semicolon separated list of all unique 3' barcode sequences associated with reads belonging to a given 5' barcode read family (both mutated and WT).

14) **barcode3_all_counts** – the number of reads in each 3' barcode group listed in the "barcode3_all" field.

15) **barcode3_all_WTcounts** – the number of the 'WT' reads in each 3' barcode group belonging to a given 5' barcode read family. For a 'WT' allele this field gets the value 'NA'.

16) **barcode3_all_WTcount** – the count of distinct 3' barcode groups associated with the high-quality mutated position that contain also 'WT' reads at this position. Does not include reads with the "BC3_Missing" tag. For a 'WT' allele this field gets the value 'NA'.

17) **barcode3_missing_all_WTcount -** a field indicating if among the reads lacking 3' barcode that are associated with the high-quality mutation (as listed in the 'Mutation' field) some reads are 'WT' at this position. It receives value of '0' if no 'WT' reads are present and '1' if one or more 'WT' reads are present. For a 'WT' allele this field gets the value 'NA'.

---

10) Identifying consensus mutations:

Step description:

This step compares mutation lists produced in the previous step to a set of user

defined cutoffs, to decide which mutations are “true” and which might represent experimental artifacts. A detailed explanation on various cutoffs used here is given in sub-step 10-2.

<u>Step 10-1: Collating mutations per read family</u>

This sub-step prepares the mutation lists produced in step 9 for consensus analysis. In the previous step, each mutation per read family was output in a separate line. Here this information is collated together. Two lines are output per read family. The first line contains a semicolon separated list of mutations found in the read family and their associated information. The second line contains information regarding the ‘WT’ reads in a given family.

Run instructions:

a) Check that the “params_1.sh” file contains the correct value for the ‘TSS’ variable.

b) **Run script:** *bash consensus_15.sh 1*

c) Wait for the job to end. Use the “sacct” and the “squeue” commands on the cluster to check job status.

d) Inspect the output files in the output folder under the “tables_consensus.BC3cutoff1” directory.

Output description:

This sub-step produces a collated list of mutations per read family. The following files are added to the output directory:

1) **<sample_name>.<ref_name>.bwa.sorted.bam.consensus.txt.1.err, <sample_name>.<ref_name>.bwa.sorted.bam.consensus.txt.out** - log files produced during the run of the job on the cluster. Check that the “.err” file does not contain error messages. Check the “.out” file for information on read families containing more than one type of mutation at the same position, and read families that were filtered out. Read families are removed from the consensus analysis if they contain only low quality mutations (mutations at positions that do not pass sequencing quality threshold, as defined in step 9). If such a family contains both ‘WT’ and mutated alleles, the ‘WT’ alleles are included in the consensus analysis, while mutations are filtered out.

Log files for analysis of 3’ barcode missing reads include the “tag-BC3_missing” tag after the “.bam” in their file name.

2) **<sample_name>.<ref_name>.bwa.sorted.bam.consensus.txt** - a collated list of mutations found in the analyzed read families, relative to the reference. Files listing mutations only in 3’ barcode missing reads include the “tag-BC3_missing” tag after the “.bam” in their file name.

The collated mutation list contains the following information:

1) **Barcode** - a unique 5’ barcode sequence serving as an identifier of a read family.

2) **Consensus** - a semicolon separated list of all high-quality mutations in the given read family (or ‘WT’. for reads not containing any mutation).

3) **Consensus_TSS** - a semicolon separated list of all high-quality mutations in the given read family, where the position of each mutation is given relative to the translation start site defined in the “params_1.sh” file. Additionally, insertions are marked by “i” and deletions by “d” instead of “-” (e.g: 48T-;51-A -> 48Td;51iA). For ‘WT’ reads, this field gets the value ‘WT’.

4) **Mutation_freqs** - a semicolon separated list of mutation frequencies within the read family, listing frequency of each mutation appearing in the “Consensus” field , when only high quality positions are considered for frequency calculation. If only ‘WT’ reads are present in the read family, but no high-quality mutations, this field gets the value ‘0’.

5) **Mutation_counts** - For each allele in the “Consensus” column, this field lists the number of reads in the family containing this allele, when sequencing quality of the position carrying the allele is above the high-quality threshold (default = 28, phred+33). If only ‘WT’ reads are present in the read family, but no high-quality mutations, this field gets the value ‘0’.

6) **total_count** – the total number of reads in a given 5’ barcode family (both mutated and WT).

7) **Positions** - the count of distinct mutations found in the read family. If only ‘WT’ reads are present in the read family, but no high-quality mutations, this field gets the value ‘0’.

8) **WT_freqs_in_mutations** - this field lists the frequencies of the ‘WT’ allele at each position where high-quality mutation was found. Calculated as: **WT_freqs_in_mutations = 1.0 - Mutation_frequency** for each mutated position.

If only 'WT' reads are present in the read family, but no high-quality mutations, **WT_freq** is calculated as the proportion of 'WT' reads out of the total number of reads in a given read family.

9) **barcode3_existing** - this field lists how many distinct 3' barcode groups are associated with each mutated allele (or 'WT' sequence) listed in the "Consensus" field. Reads carrying the same 3' barcode sequence are counted as the same 3' barcode group.

10) **barcode3mut_above1reads** - this field lists how many distinct 3' barcode groups containing more than one read are associated with each mutated allele listed in the "Consensus" field. If only 'WT' reads are present in the read family, but no high-quality mutations, this field gets the value 'NA'.

11) **barcode3WT_above1reads** - this field lists how many distinct 3' barcode groups associated with each mutated allele listed in the "Consensus" field contain more than one read with 'WT' nucleotide at this position, in addition to the mutated reads. If only 'WT' reads are present in the read family, but no high-quality mutations, this value equal to the value listed in the "barcode3_above1reads" field (see below).

12) **barcode3_above1reads** - this field lists how many distinct 3' barcode groups within given 5' barcode read family contain more than one read (mutated or WT).

13) **barcode3_all_WTcount** - for each mutation listed in the "Consensus" field, this field lists the number of 3' barcode groups associated with it that contain 'WT' reads in addition to the mutated ones.

14) **unmutatedReads_counts** - this field lists how many reads in a given 5' barcode family are 'WT' (does not contain any mutations in the user defined region of interest, as explained in step 9).

15) **unmutatedReads_freq** - this field lists the frequency of 'WT' reads in a given 5' barcode family (proportion of 'WT' reads out of the total number of reads in the family).

Step 10-2: Detecting consensus mutations

This sub-step compares the collated mutation list, produced in sub-step 10-1, against a set of user defined thresholds. Mutations that pass the thresholds are output in the consensus list. Positions where both mutated and 'WT' alleles do not pass the thresholds are considered ambiguous and are output as 'N'. If all mutations are rejected, but their associated 'WT' positions pass the thresholds, the read is

considered ‘WT’.

Run instructions:

a) Check that the “factors_table.txt” file contains the correct value in the column: “size_r”. In case of an incorrect value, adjust the table and **repeat step 2** described above to produce a correct bash parameter file, before starting this step.

b) **Run script:** *bash consensus_15.sh 2*

c) Wait for the job to end. Use the “sacct” and the “squeue” commands on the cluster to check job status.

d) Inspect the output files in the output folder under:

“tables_consensus.BC3cutoff1/<sample_name>.<ref_name>.bwa.sorted.bam.”;

“tables_consensus.BC3cutoff1/<sample_name>.<ref_name>.bwa.sorted.bam..cutoffs-update”

To indicate output of analysis of reads lacking 3’ barcode, the “tag-BC3_missing” tag is added to the output directory name after “.bam”.

Threshold description:

In this sub-step, mutated and ‘WT’ alleles found in the analyzed sample are compared against a set of thresholds to determine if they can be considered “true” mutations. The comparison is done iteratively against increasing threshold values. This allows the user to explore how the results of the analysis change if different cutoffs are used and to choose an optimal set of cutoffs to create a list of consensus mutations for further analyses.

To change the thresholds used in this step, the user should change the relevant threshold values in the “consensus_cutoffs_15.py” script. The following thresholds are used to determine the “consensus” mutations:

1) **min_count** – the minimal 5’ barcode read family size. Read families having fewer reads than this threshold are skipped and are not checked against additional thresholds.

2) **min_freq** – the minimal frequency of mutated or WT allele in the read family. Alleles not passing this frequency threshold are not considered “consensus”.

3) **minCountBarcode** – the minimal number of distinct 3’ barcode groups associated with a given allele within the read family. Alleles not passing this threshold are not

considered “consensus”.
4) **bc3groupsWithReadsAbove1_OK** – the minimal number of distinct 3’ barcode groups associated with a given allele within the read family that have more than one read in the group. Alleles need to pass either this or the “bc3groupCountOK” threshold to count as “consensus”.
5) **bc3groupCountOK** – the minimal number of distinct 3’ barcode groups associated with a given allele. Alleles need to pass either this or the “bc3groupsWithReadsAbove1_OK” threshold to count as “consensus”.

Note: Both the “**minCountBarcode**” and the “**bc3groupCountOK**” variables are checking the number of distinct 3’ barcode groups associated with a given allele. Two variables, checking against the same condition, were created to allow selecting consensus mutations based either on the number of 3’ barcode groups associated with each mutation or the size of said groups.
The full condition for checking the 3’ barcode group information is [*minCountBarcode* ***and*** *(bc3groupCountOK* ***or*** *bc3groupsWithReadsAbove1_OK)*]. Thus, if “**minCountBarcode**” >= “**bc3groupCountOK**”, any allele passing one threshold would also pass the other one. Otherwise, the allele would need to pass the “**bc3groupsWithReadsAbove1_OK”** threshold, in addition to “**minCountBarcode**”, to be considered as “consensus” (assuming that it has passed all other thresholds).
From data perspective it means that either larger number of 3’ barcode groups of any size, or smaller number of 3’ barcode groups, but with larger number of reads in each group, is required to consider the particular allele as “consensus”. The aim is to reduce the possibility that an identified allele is actually an artifact and not a real mutation, by ensuring that each mutation counted as “consensus” is supported by a large enough number of reads from different groups.

Output description:
The mutation filtering sub-step produces four directories for each reference against which the reads are aligned within the “tables_consensus.BC3cutoff1” directory. Two directories contain results of the analysis of all reads, and two contain results of 3’ barcode lacking reads’ analysis. The latter carry the “tag-BC3_missing” tag in their folder name, and are created only if the previous steps identified high quality mutations in the group of 3’ barcode lacking reads. The difference between the

directories is the type of threshold sets used to identify “consensus” alleles:

1) **<sample_name>.<ref_name>.bwa.sorted.bam.,**
<sample_name>.<ref_name>.bwa.sorted.bam.tag-BC3_missing - here the allele needs to be associated with a certain number of distinct 3’ barcode groups to be considered as “consensus”. There are no limits on the size of the 3’ barcode groups. The following thresholds are used:
a) **min_count** - [0 - 10, 25, 50]
b) **min_freq** - [0.0 - 1.0], with 0.1 intervals.
c) **minCountBarcode** - [0 - 5]
d) **bc3groupsWithReadsAbove1_OK** - 0
e) **bc3groupCountOK** - 0

2) **<sample_name>.<ref_name>.bwa.sorted.bam..cutoffs-update,**
<sample_name>.<ref_name>.bwa.sorted.bam.tag-BC3_missing.cutoffs-update - here the allele either needs to be associated with a certain number of distinct 3’ barcode groups, or has to have more than one read in a certain number of these groups to be considered as “consensus”. The following thresholds are used:
a) **min_count** - [0 - 10, 25, 50]
b) **min_freq** - [0.0 - 1.0], with 0.1 intervals.
c) **minCountBarcode** - [0 - 5]
d) **bc3groupsWithReadsAbove1_OK** - 2
e) **bc3groupCountOK** - 3

In addition to the above, the script can also handle samples where reads were barcoded only at their 5’ end. In this case, the script would still produce different directories as described above, but in both directories it would use the same set of thresholds to determine “consensus” alleles:
a) **min_count** - [0 - 10, 25, 50]
b) **min_freq** - [0.0 - 1.0], with 0.1 intervals.
c) **minCountBarcode** - 0
d) **bc3groupsWithReadsAbove1_OK** - 0
e) **bc3groupCountOK** - 0

Within each directory, two files are created for each tested combination of threshold values:

1)

<sample_name>.<ref_name>.bwa.sorted.bam.mutFreq<W>_readCount<X>_BC3WithMut<Y>_BC3above<Z>.txt - a list of “consensus” mutations that passed the thresholds. Its structure is the same as of the collated mutation list file created in step 10-1.

A separate file is created for each combination of thresholds used to determine “consensus” mutations and threshold values are listed in its file name:

a) **mutFreq<W>** - the mutation frequency threshold.

b) **readCount<X>** - the size of the 5’ barcode family threshold (number of reads).

c) **BC3WithMut<Y>** - the threshold on the number of distinct 3’ barcode groups associated with the allele.

d) **BC3above<Z>** - the threshold on the number of distinct 3’ barcode groups associated with the allele that have more than one read in the group.

In the “consensus” list the consensus is determined between mutated and ‘WT’ alleles in the 5’ barcode family at each position of mutation.

If at any position the mutated allele is rejected, because it did not pass the thresholds, but the ‘WT’ allele is accepted, this position would be considered ‘WT’ and **would not be reported** in the consensus mutation list. If in a given read family **all** mutations are rejected, but their associated ‘WT’ alleles are **all** accepted, the family would be reported as ‘WT’ in the consensus list.

If both mutation and ‘WT’ alleles are not accepted, the position would be considered ambiguous and reported as ‘N’ in the consensus mutation list (e.g. - 39CT -> 39N). If multiple mutations at the same position are found to be ambiguous, they would be represented by a single entry and their associated information (such as mutation frequencies) would be summed up.

If the family contains only ‘WT’ reads, but no mutations, it is included in the consensus list only if it passes all the thresholds. Otherwise it is not reported.

2)

<sample_name>.<ref_name>.bwa.sorted.bam.mutFreq<W>_readCount<X>_BC

3WithMut<Y>_BC3above<Z>.cons-count.txt - the accompanying file to each list of consensus mutations that counts the number of 5' barcode families in the list having a particular mutation profile. In the left column it lists all mutation profiles found in the consensus list, and in the right column, their count. In addition, it provides information on rejected families:

a) **rejected.low_count** - families that were rejected because they did not pass the read count threshold (size of the 5' barcode family is too small).

b) **rejected.from_WThaplotype** - 'WT' read families that were rejected because they did not pass either of the 3' barcode group associated thresholds.

c) **rejected.other** - families rejected for reasons other than the ones mentioned above.

In addition, cluster log files are created under the "tables_consensus.BC3cutoff1" folder in the output directory. The log files are:

1) **<sample_name>.<ref_name>.bwa.sorted.bam.consensus.txt.2.err, <sample_name>.<ref_name>.bwa.sorted.bam.consensus.txt.2.out**

2) **<sample_name>.<ref_name>.bwa.sorted.bam.consensus.txt.cutoffs-update.2.err, <sample_name>.<ref_name>.bwa.sorted.bam.consensus.txt.cutoffs-update.2.out**

The log files for analysis of the 3' barcode missing reads include the "tag-BC3_missing" tag after the ".bam" part of their file name. Upon run completion, check that the ".err" files do not contain any error messages, to indicate that the run was completed successfully.
