# Supplementary material for: De novo mutation rates at the single-mutation resolution in a human HBB gene region associated with adaptation and genetic disease
Source: Genome Res. 2022 Mar;32(3):488–98. doi: 10.1101/gr.276103.121 (PMC8896469; doi:10.1101/gr.276103.121)
Supplement: Supplemental Material [file supp_gr.276103.121_SUPPLEMENTAL_v1.1.zip › documents/Quick_run.pdf]

Introduction

The MEMDS analysis pipeline is a software package accompanying the MEMDS sequencing protocol. The pipeline analyzes deep-sequencing data produced by MEMDS and outputs summary tables of mutations found in the analyzed data relative to the reference gene(s).

Quick run instructions

This section provides basic information needed to run the pipeline. For a more detailed information regarding each analysis step and its associated output, refer to the next section.

Note 1: The "**$i**" symbol is used to indicate that a numerical option that should be provided after the command name, with available options listed in parentheses.
For example: *bash my_script.sh $i (1-3)* means that **my_script.sh** accepts numerical options 1 to 3. To complete the step, **my_script.sh** should be run with all the options, sequentially, according to the order defined in parentheses.

Note 2: When running on a cluster, for each job submitted by the script the following message would appear: "Submitted batch job #job_serial_number".
Before moving to the next option in the script or to the next step in the pipeline, a**lways check that all running jobs were completed successfully.** To check job status, use the following commands (for SLURM systems):
1) *squeue -u "username"| grep -c "username"* – this command displays the number of jobs in the queue for the account "username". Completed or canceled jobs would be removed from the queue.

2) *sacct -u "username"* – this command lists all the jobs that ran on the account "username" in the current session. The last column indicates for each job if it is completed, canceled, or still running. Ensure that all relevant jobs have 'Completed' status before submitting new ones!

In addition, remember to check the ".err" and ".out" log files produced during job

runs on the cluster. Log files are placed in the same directory as the result files produced by the pipeline in each step or sub-step. They record warnings and error messages raised by the script or by the cluster during a job run.

<u>Run instructions:</u>

1) Place the folder containing the pipeline scripts and associated parameter files in the same directory as the folder with the analyzed files.

2) Navigate to the script directory from the command line (***cd /path/to/projects/scripts***) to use the pipeline. All pipeline commands should be run from within the script directory.

3) **(Optional) Merging raw data:**
   a) *bash concatenate_partfiles.sh*
   b) **Local run:** *bash more_scripts/samples_table_0.sh.concat.sh* **or**
   c) **Cluster run:** *srun bash more_scripts/samples_table_0.sh.concat.sh*

4) **Formatting parameter data for use by the pipeline:**
   a) **Single-end data:** *bash setting_1-SE.sh* **or**
   b) **Paired-end data:** *bash setting_1-PE.sh*

5) **Quality control and clearing of raw data + paired-end data merging**:
   a) **Single-end data:** *bash filter-SE4.sh $i* (1-3) **or**
   b) **Paired-end data:** *bash filter-PE4.sh $i* (1-4)

6) **Selecting reads with correct barcodes and separating between barcodes and genomic data**:
   a) *bash trim7.sh 1*

7) **Sorting reads by their origin gene:**
   a) *bash sort2.sh 1*

8) **Mapping reads to the reference sequences:**
   a) *bash bwa9.sh $i* (1-2)

9) **Making alignment files viewable in IGV:**

a) *bash create_dummy_genome5.sh 1*

10) **Creating a mutation table that lists sequencing quality alongside each mutation:**

a) *bash sam_to_mutation-list-3.sh 1*

11) **Creating a table of mutations found in the analyzed data:**

a) *bash sam_to_mutation-table_5.0.sh 1*

12) **Detecting consensus mutations that pass a set of user-defined thresholds:**

a) *bash consensus_15.sh $i* (1-2)
